# Generation of boryl-nitroxide radicals from a boraalkene *via* the nitroso ene reaction†

**DOI:** 10.1039/d2sc02485c

**Published:** 2022-08-18

**Authors:** Chaohuang Chen, Constantin G. Daniliuc, Sina Klabunde, Michael Ryan Hansen, Gerald Kehr, Gerhard Erker

**Affiliations:** Organisch-Chemisches Institut, Westfälische Wilhelms-Universität Münster Corrensstraße 40 48149 Münster Germany erker@uni-muenster.de; Institut für Physikalische Chemie, Westfälische Wilhelms-Universität Münster Corrensstraße 28/3040 48149 Münster Germany

## Abstract

Examples of isolated boron substituted nitroxide radicals are rare. The reaction of the reactive cyclic boraalkene 3 with nitrosobenzene yields a mixture of the [2 + 2] cycloaddition product 4a, the *B*-nitroxide radicals 5a and 6a and the azoxybenzene co-product 7a*via* a bora nitroso ene reaction pathway, the boron analogue of the nitroso ene reaction. The products were separated by flash chromatography, and the *B*-nitroxide radicals were characterized by X-ray diffraction and EPR spectroscopy. Radical 5a was shown to be a hydrogen atom abstractor. Both the *B*-nitroxide radicals are more easily oxidized compared to *e.g.* TEMPO, as shown by cyclic voltammetry.

## Abstract 2

The NHC-stabilized boraalkene reacts with nitrosobenzene to give a [2+2] cycloaddition product plus a pair of persistent borylnitroxide radicals. These are thought to be formed by means of a bora nitroso ene reaction followed by H-atom transfer.

## Graphical abstract

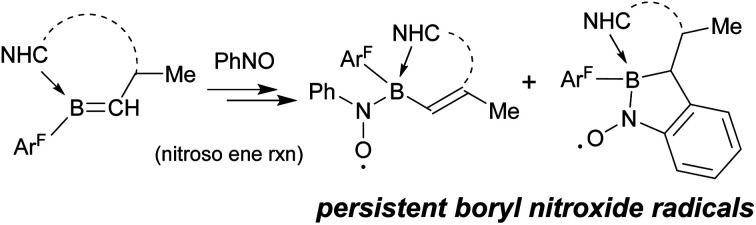

## Introduction

Nitroxide radicals are an important class of compounds. The TEMPO aminoxyl radical and its derivatives have found extensive use in organic and polymerization chemistry and beyond.^1^ The defined redox chemistry of the nitroxide radicals has been used in stoichiometric and catalytic oxidation processes,^2^ and nitroxides have been employed as ligands in transition metal coordination chemistry.^3^ While numerous substituted and functionalized aminoxyl radicals have become known, examples of boron containing nitroxide radicals are less frequently encountered.^4–9^

**Scheme 1:**
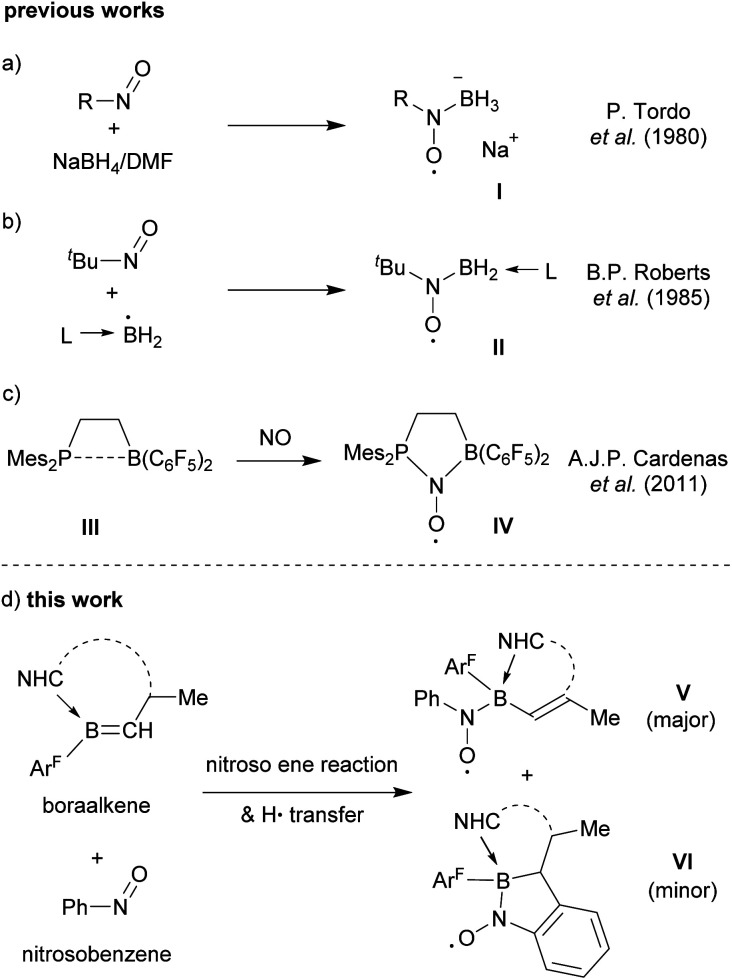
Examples of boron containing nitroxide radicals.

Tordo *et al.* reported the generation of the nitroxide radical anion I by treatment of 2-methyl-2-nitrosopropane with NaBH_4_/DMF (Scheme 1a).^5^ The *in situ* generated BH_3_^−^˙ radical anion was detected using spin traps.^6^ B. P. Roberts *et al.*^7^ used a number of nitroso alkanes and nitroso arenes as spin traps to successfully detect a variety of ligated ·BH_2_ radicals^8^ as verified by EPR spectroscopy (Scheme 1b). Our group had used the special features of frustrated Lewis pair (FLP) chemistry to prepare persistent *P*/*B*-nitroxide radicals, that were isolated and characterized by spectroscopy and by X-ray crystal structure analysis. The formation of compound IV*via* exposure of the intramolecular ethylene bridged P/B FLP III to nitric oxide under mild conditions is a typical example (Scheme 1c).^9^

We have now found that examples of boryl-nitroxide radicals can be made by treatment of a suitably substituted boraalkene with nitrosobenzene by a pathway that involves a variant of the nitroso ene reaction.^10^ The radicals of type V and VI were obtained as examples of this class of compounds. They were thoroughly characterized including EPR spectroscopy and X-ray diffraction (Scheme 1d).

## Results and discussion

**Scheme 2:**
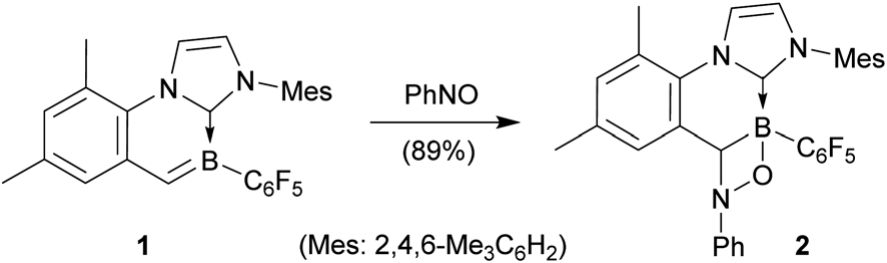
Reaction of cyclic boraalkene 1 with nitrosobenzene.

We had described that cyclic boraalkene 1 was readily prepared starting from the corresponding [(IMes)(C_6_F_5_)BH]^+^ borenium cation (with the [B(C_6_F_5_)_4_]^−^ anion)^11^ by a sequence involving thermally induced intramolecular C–H activation at an *ortho*-methyl group of an NHC mesityl substituent (with H_2_ formation) followed by deprotonation.^12^ Compound 1 was now found to rapidly react with nitrosobenzene^13^ (C_6_D_6_, r.t., 5 min) to yield the [2 + 2] cycloaddition product 2 (see Scheme 2).

The X-ray crystal structure analysis of compound 2 showed bond lengths inside the newly formed four-membered heterocycle of 1.505(4) Å (B1–O1), 1.624(4) Å (B1–C17), 1.504(3) Å (N3–C17) and 1.475(3) Å (N3–O1). In solution (C_6_D_6_), this unit showed NMR features at *δ* −4.1 (^11^B) and *δ* 69.1/4.57 (^13^C/^1^H), respectively (see the ESI† for further details and the depicted structure).

**Scheme 3:**
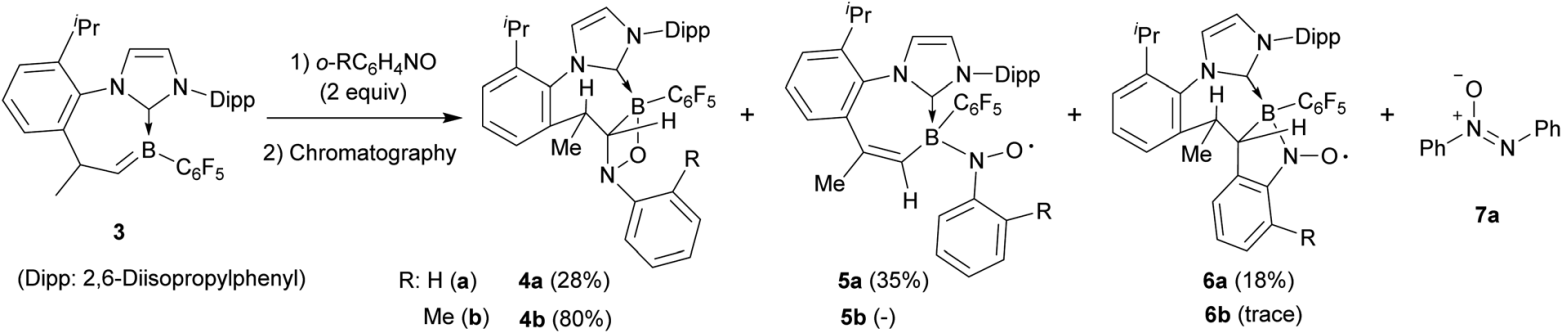
Formation of two boryl-nitroxide radicals 5a and 6a from the reaction of the cyclic NHC-stabilized boraalkene 3 with nitrosobenzene. The yields given refer to the isolated material (see the ESI† for details).

Boraalkene 3 was prepared from the corresponding borenium salt [(IPr)(C_6_F_5_)BH]^+^[B(C_6_F_5_)_4_]^−^ by a procedure analogous to the formation of 1, as previously described.^12^ Boraalkene 3 was reacted with 2 molar equiv. of nitrosobenzene. After a reaction time of 1 h (r.t. in C_6_D_6_), the components of the product mixture were separated by gradient elution flash chromatography. In this case, the [2 + 2] cycloaddition product (4a, isolated as an off-white solid in 28% yield) was not the sole product in contrast to the above described nitrosobenzene reaction with boraalkene 1. The major product isolated was the persistent nitroxide radical 5a (35% yield), and it was accompanied by the isomeric nitroxide radical 6a in a yield of 18%. Azoxybenzene 7a was obtained as a necessary co-product of the nitroxide radical formation (see Scheme 3).^14^ In a second experiment, it was shown that compounds 4a–6a could also be obtained by treating compound 3 with 1 equiv. of nitrosobenzene and subsequent exposure to air (see the ESI† for details).

**Fig. 1:**
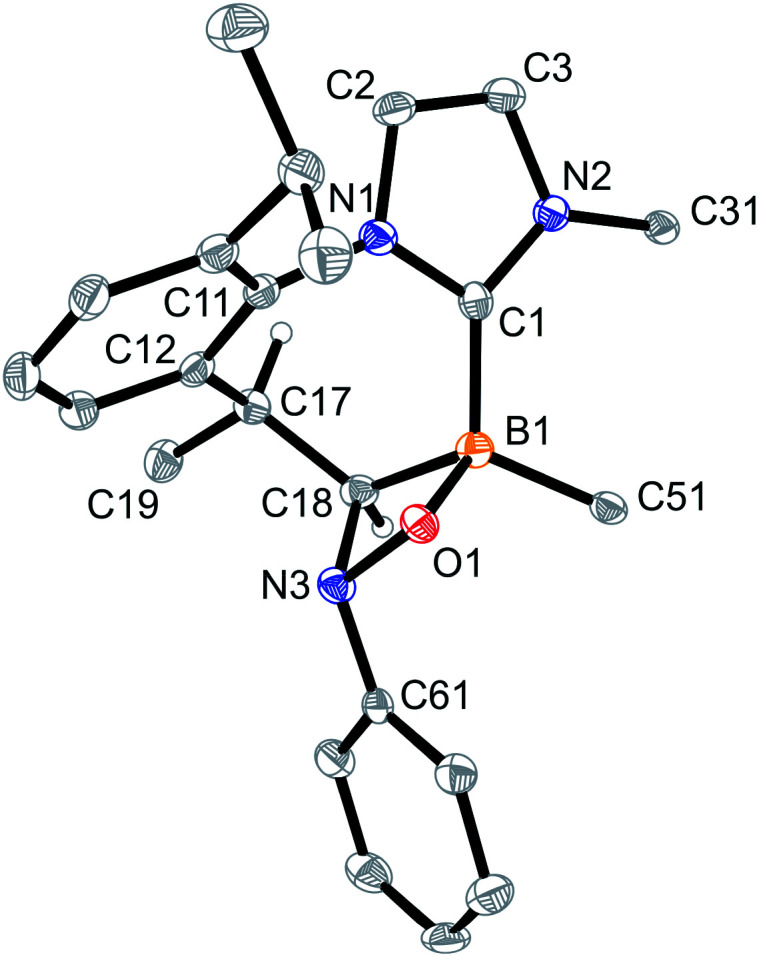
A view of the molecular structure of the boraalkene/nitrosobenzene [2 + 2] cycloaddition product 4a [thermal ellipsoids are set at 50% probability; only the *ipso*-C_6_F_5_/Dipp carbon atoms C51/C31 are shown for clarity, and H atoms are omitted (except for C17H and C18H)]. Selected bond lengths (Å) and angles (°): B1–O1 1.512(2), B1–C1 1.619(3), B1–C18 1.636(3), C17–C18 1.547(2), O1–N3 1.481(2), N3–C18 1.503(2), C18–N3–O1 93.6(1), C19–C17–C18–N3 −86.2(2), C18–N3–O1–B1 −16.5(1), and ΣN3^CCO^ 330.0.

The products 4a–6a were obtained crystalline and characterized by X-ray crystal structure analysis. The structure of the PhNO adduct 4a (see Fig. 1) shows the newly formed CNOB containing four-membered heterocyclic ring system annulated with the central seven-membered core that had been generated by the initial C–H activation process during the synthesis of the starting material 3. The seven-membered heterocycle shows a boat-shaped conformation with the methyl substituent at its saturated C(sp^3^) tip oriented equatorially.

In solution (CD_2_Cl_2_, 299 K), compound 4a shows a ^11^B NMR signal at *δ* −0.7. The NMR signals of the CH group inside the annulated four-membered heterocyclic ring system occur at 80.7 (^13^C) and 3.44 (^1^H), respectively.

**Fig. 2:**
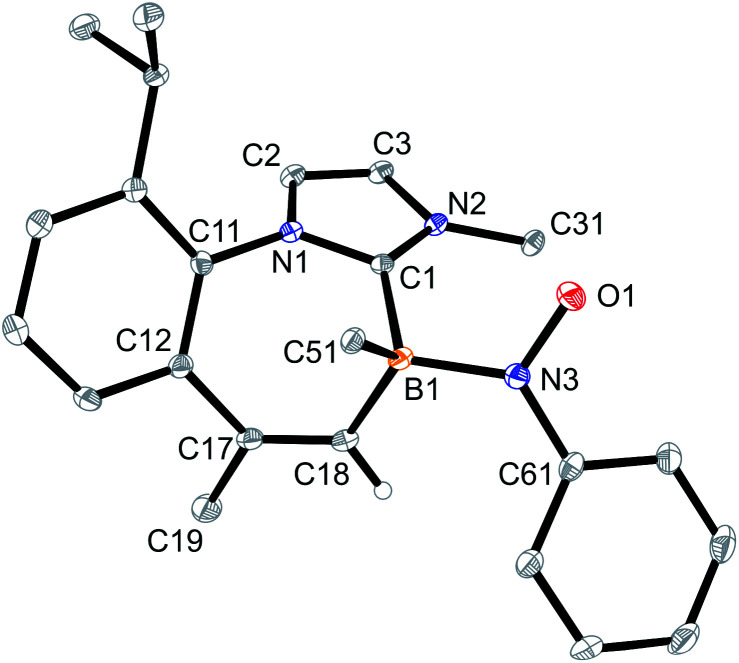
Molecular structure of the BNO nitroxide radical 5a [thermal ellipsoids are set at 30% probability; only the *ipso*-C_6_F_5_/Dipp carbon atoms C51/C31 are shown for clarity, and H atoms are omitted (except for C18H)]. Selected bond lengths (Å) and angles (°): B1–C1 1.631(2), B1–C18 1.615(2), B1–N3 1.558(2), C17–C18 1.339(2), N3–O1 1.304(2), B1–N3–O1 115.4(1), C1–B1–N3–O1 54.0(2), and ΣN3^BCO^ 359.9.

Crystallization of the obtained brown solid from CH_2_Cl_2_/pentane gave compound 5a as dark-red crystals. The X-ray crystal structure analysis of the nitroxide radical 5a (see Fig. 2) also shows a central 1,3-azaborepine derived core, but in this case, the boron atom represents the tip of the boat-shaped structure. Adjacent to it are a newly formed endo-cyclic carbon–carbon double bond and the N–C moiety of the annulated imidazolylidene unit. The Dipp derived annulated isopropylphenylene unit is oriented at the distal position to the boron tip. Boron atom B1 bears the C_6_F_5_ substituent and the newly formed –N(–O·)Ph nitroxide radical building block. The latter is found in an equatorial orientation at the central seven-membered ring. Compound 5a represents a rare example of a boron substituted persistent nitroxide radical. Its B–N–O subunit shows bond lengths of 1.558(2) Å (B1–N3) and 1.304(2) Å (N3–O1) (angle B1–N3–O1 115.4(1)°), which indicates that the boron heteroatom mainly inductively interacts with the radical moiety.

**Fig. 3:**
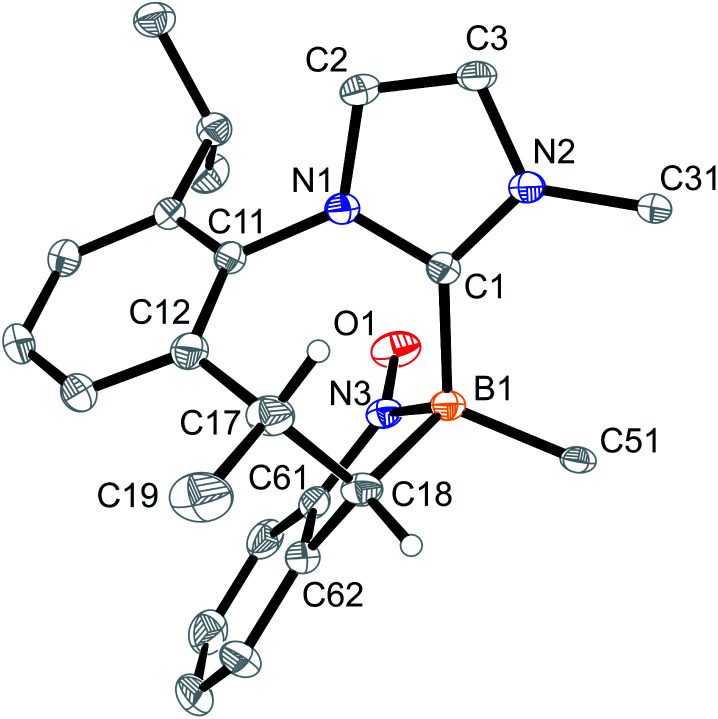
A projection of the molecular structure of the boron-containing nitroxide radical 6a [thermal ellipsoids are set at 30% probability; only the *ipso*-C_6_F_5_/Dipp carbon atoms C51/C31 are shown for clarity, and H atoms are omitted (except for C17H and C18H)]. Selected bond lengths (Å) and angles (°): B1–C1 1.633(3), B1–C18 1.649(3), B1–N3 1.563(3), C17–C18 1.570(3), N3–O1 1.289(2), N3–C61 1.386(3), B1–N3–O1 126.2(2), O1–N3–B1–C1 44.5(3), and ΣN3^BCO^ 360.0.

Crystallization of the obtained yellow solid from CH_2_Cl_2_/pentane gave the minor *B*-nitroxide radical compound 6a as orange crystals. The X-ray crystal structure analysis of compound 6a shows the seven-membered core with the CHCH_3_ moiety as the tip of the boat conformation (see Fig. 3). Adjacent to it is the newly formed annulated five-membered ring that contains the integrated NO radical functionality. This ring has an annulated phenylene moiety that originates from the nitrosobenzene reagent. We note that the B1–C1 bonds in radicals 5a and 6a are marginally longer than in compound 4a.

**Fig. 4:**
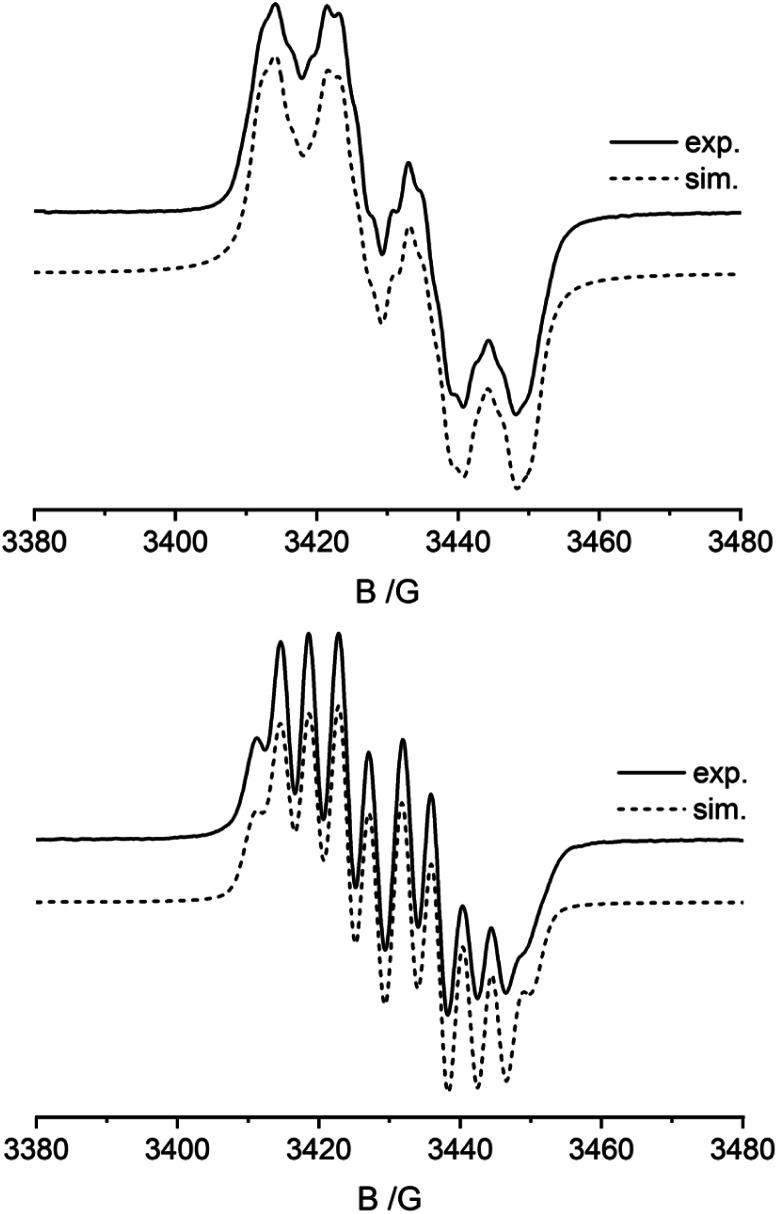
Liquid state CW-EPR spectra (CH_2_Cl_2_, r.t.) and lineshape simulations of the *B*-nitroxide radicals 5a (top) and 6a (bottom). Tables S1 and S2† summarize the EPR lineshape simulation parameters.

Radicals 5a and 6a were characterized by C,H,N-elemental analysis and by CW-EPR spectroscopy. Fig. 4 (top) shows the EPR spectrum of compound 5a. The EPR signal displays a *g*-factor of 2.0066 with a hyperfine-coupling with nitrogen [*A*(^14^N) = 26.06 MHz], boron [*A*(^11^B) = 12.50 MHz], and to the five hydrogen atoms of the phenyl substituent [*A*(^1^H) = 6.31, 2.58, and 1.42 MHz]. The EPR spectrum of compound 6a (Fig. 4, bottom) shows similar parameters (*g* = 2.00604 with hyperfine couplings: *A*(^14^N) = 25.18 MHz, *A*(^11^B) = 11.07 MHz, and *A*(^1^H) = 9.65, 0.58, and 0.43 MHz). The hyperfine coupling constants *A*(^14^N) of 5a and 6a are markedly smaller than found in the *N*-oxyl radicals TEMPO (43.5 MHz) or ^*t*^Bu_2_NO (43.3 MHz).^1,15^ The *A*(^11^B) values of the 5a/6a pair are only slightly higher than what is reported for the P/B FLP-NO radical IV (9.1 MHz, see Scheme 1c).^9^ DFT calculations show that the spin density (probability that an unpaired electron is located at the nucleus) for radicals 5a and 6a resides on oxygen, nitrogen, boron, and the adjacent phenyl (5a) or phenylene (6a) groups (see ESI, Fig. S31†). Furthermore, the probability of an unpaired electron residing at ^14^N is higher than at ^11^B in accordance with the observed EPR parameters (see the ESI for details; see Fig. S25† for the UV-vis spectra of compounds 5a and 6a).

The ratio of the radical products 5a and 6a*vs.* the [2 + 2] cycloaddition product 4a is slightly solvent dependent. The highest radical yields were obtained from the reactions in benzene or toluene, whereas reactions in more polar solvents (acetonitrile or DMSO) gave smaller amounts of these nitroxide radicals (see the ESI† for details).

Boraalkene 3 was also reacted with the bulkier 2-nitrosotoluene. From this reaction, we obtained the [2 + 2] adduct 4b in a yield of 80%. It was characterized spectroscopically and by C,H,N elemental analysis (see the ESI† for details). We could not isolate any of the respective nitroxide radicals 5b or 6b from this experiment. However, an EPR signal was recorded from the *in situ* experiment before workup. The spectrum in the ESI† indicates that radical 6b was probably formed in this reaction, although as a very minor product.

**Scheme 4:**
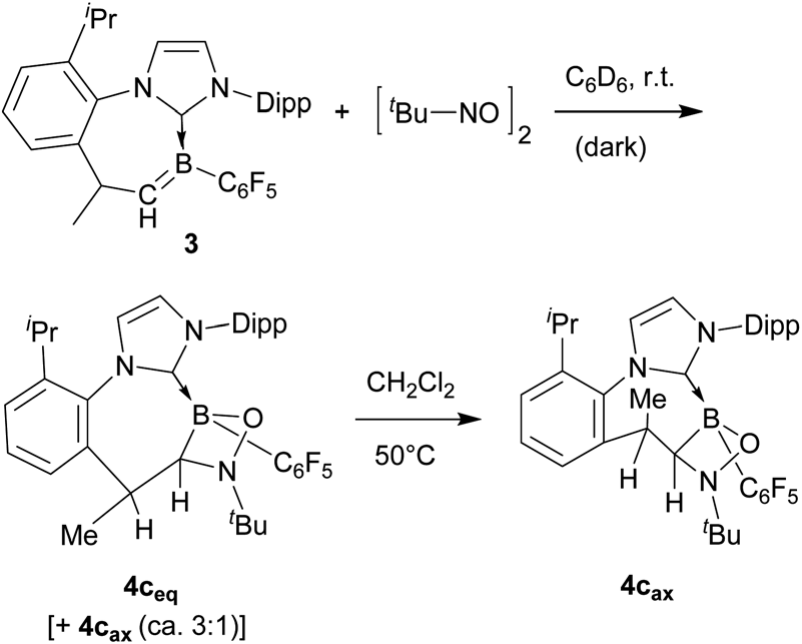
Reaction of boraalkene 3 with 2-methyl-2-nitrosopropane.

**Fig. 5:**
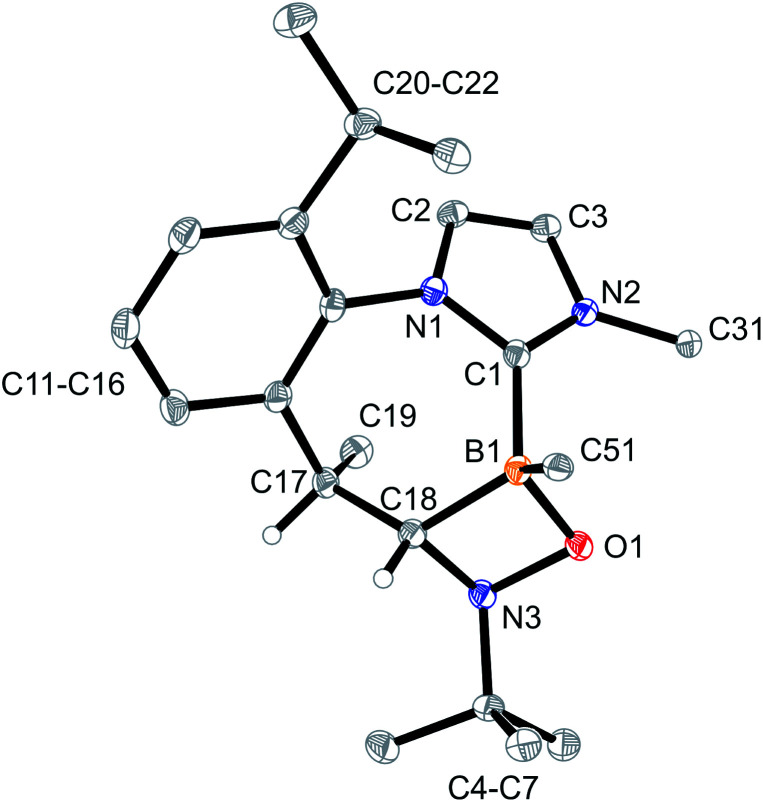
A projection of the molecular structure of the 3 plus *t*Bu-NO [2 + 2] cycloaddition product 4c [thermal ellipsoids are set at 30% probability; only the *ipso*-C_6_F_5_/Dipp carbon atoms C51/C31 are shown for clarity, and H atoms are omitted (except for C17H and C18H)]. Selected bond lengths (Å) and angles (°): B1–O1 1.495(3), B1–C1 1.635(3), B1–C18 1.636(3), N3–O1 1.497(3), N3–C18 1.496(3), and ΣN3^CCO^ 319.6.

The reaction of boraalkene 3 with the aliphatic 2-methyl-2-nitrosopropane reagent (D_6_-benzene, 14 h, r.t.) took a similar course. Work-up furnished the [2 + 2] cycloaddition product 4c in *ca.* 90% yield as a mixture of two persistent conformational isomers (4c_eq_ and 4c_ax_) in a *ca.* 3 : 1 molar ratio. A slow conformational isomerization was observed in solution, eventually resulting in the almost pure thermodynamic isomer 4c_ax_ (see Scheme 4). This was characterised by X-ray diffraction (see Fig. 5). It shows a typical cycloheptatriene reminiscent boat shaped conformation of the central seven-membered ring with the methyl substituent at the C(sp^3^) tip oriented in an axial position. As in compound 4a, the methyl substituent is *cis*-oriented to the annulated four-membered ring, only in the conformationally inverted situation. The *in situ* generated reaction solution showed an EPR signal of a minor as yet unidentified nitrogen containing radical component. The EPR spectrum is depicted in the ESI.†

**Scheme 5:**
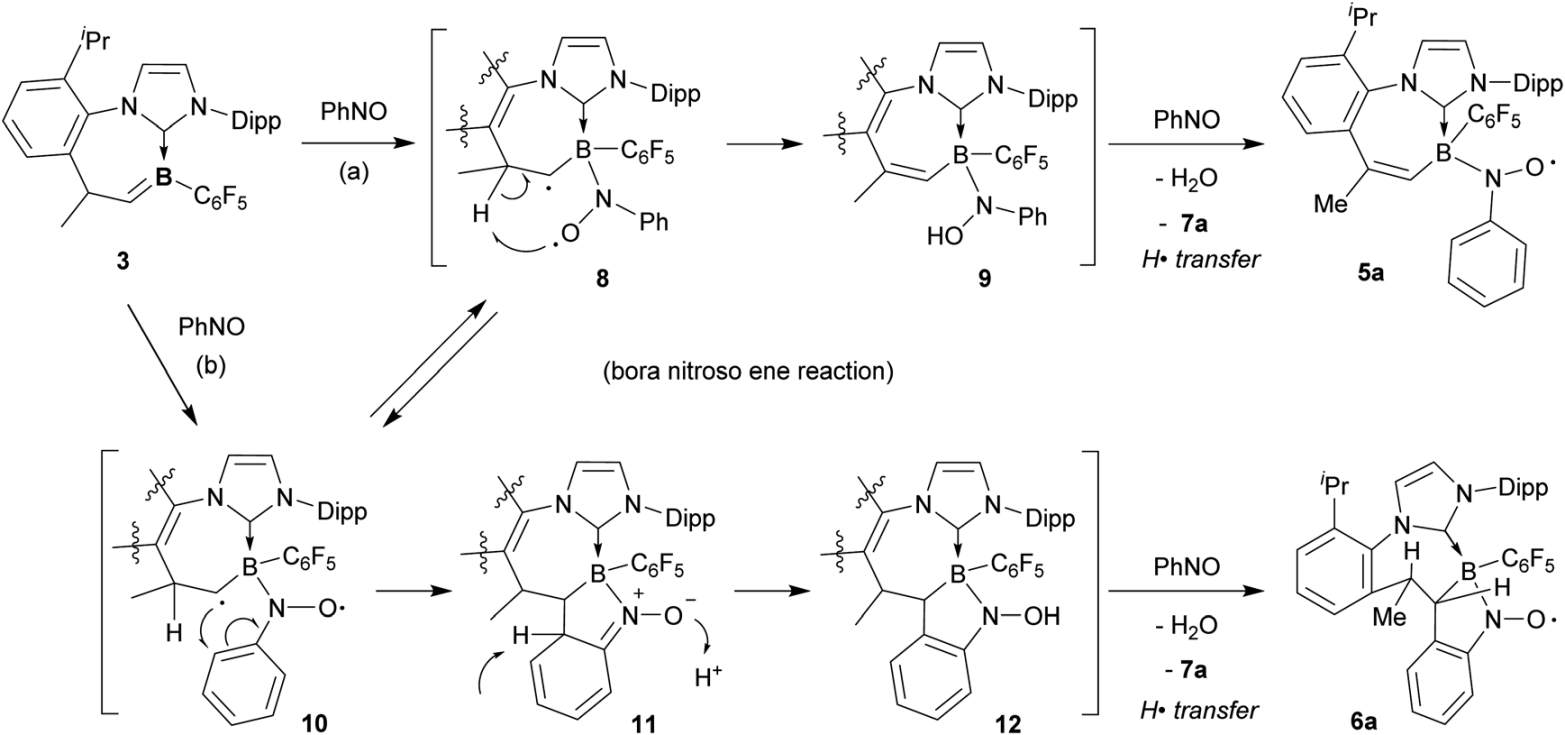
Possible pathways leading to the boryl nitroxide radicals 5a and 6a.

For the mechanistic scheme leading to the formation of the persistent B–N-oxyl radicals 5a and 6a, we propose that this reaction occurs by a variant of the nitroso ene reaction, here the bora nitroso ene reaction (see Scheme 5). It had been discussed that two different essential types of intermediates may be involved in the nitroso ene reaction, namely an aziridine *N*-oxide^10*b*^ or a polarized diradical.^10*a*^ The diradical pathway would serve to explain the formation of both the isomeric nitroxide radicals 5a and 6a to potentially occur through the same intermediate. Compound 8 would be generated by nitroso arene coordination to boron through nitrogen. Subsequent internal H-abstraction would then directly give the bora nitroso ene product, the “boryl hydroxylamine” derivative 9 which is only one hydrogen atom transfer step from the obtained product 5a. Apparently, the nitrosobenzene reagent serves as the H· acceptor to give 5a and azoxybenzene (after H_2_O elimination; see Fig. S61 in the ESI† for an *in situ* experiment on the generation of 5a from 9). In a competing reaction branch, the cyclic radical of conformer 10 of the diradical intermediate could attack the phenyl ring to generate intermediate 11. Tautomerization would give the N–OH product 12 which would provide the observed product 6a by its subsequent reaction with nitrosobenzene.

**Scheme 6:**
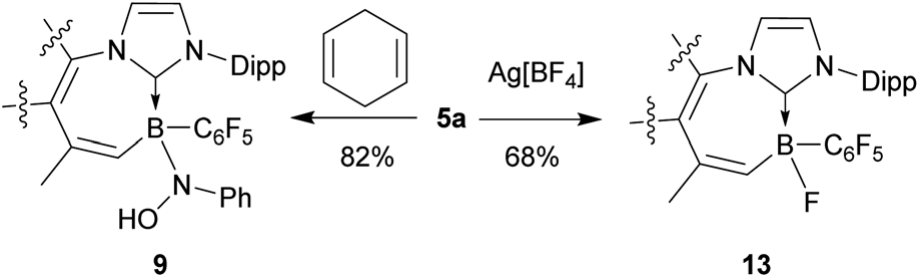
Reactions of BNO radical 5a.

The newly formed products undergo some typical nitroxide radical reactions.^1^ Compound 5a readily abstracts a hydrogen atom from 1,4-cyclohexadiene to form the corresponding diamagnetic N–OH product 9 (see Scheme 6). It shows the ^1^H NMR N–OH signal at *δ* 3.36 (in CD_2_Cl_2_ at 299 K) and a ^11^B NMR resonance at *δ* −8.5. Compound 9 was characterized by X-ray diffraction. It shows very similar general structural parameters to its precursor 5a, only the N3–O1 linkage in 9 is much longer at 1.467(2) Å (*cf.* 1.304(2) Å in 5a). We also note that the C1–B1 linkage to the N-heterocyclic carbene ligand is slightly longer in radical 5a (B1–C1 Å 1.631(2)) than in the N–OH compound 9 (B1–C1 1.623(2) Å) (see the ESI† for the single crystal X-ray structure of compound 9).

The boron substituent has an influence on the reactivity properties of the *B*-nitroxide radicals. Both the *B*-nitroxide radicals 5a and 6a are more easily oxidized than *e.g.* TEMPO. The cyclic voltammograms show redox features with *E*_1/2_ = 0.53 V for 5a and 0.28 V for 6a (rel. Ag/Ag^+^), respectively [*cf.* TEMPO *E*_1/2_ = 0.66 V (rel. Ag/Ag^+^)]; see the ESI† for details).^16^ We tried to oxidize radical 5a to an oxoammonium cation by using Ag[BF_4_] as a chemical oxidant in CH_2_Cl_2_ (r.t., 30 min). After workup including flash chromatography (SiO_2_), compound 13 was obtained as a white solid, and its structure was confirmed by X-ray single crystal analysis and by NMR spectroscopy (see the ESI† for details). This result indicates that the boryl NO radical indeed could be further oxidized, but the desired boryl oxoammonium cation was not stable under the reaction conditions; it underwent a substitution reaction to form the observed product 13.

Radical 6a was used in a Cu-catalyzed oxidation process of cinnamyl alcohol in a procedure similar to one described by Stahl *et al.*^17^ to give cinnamaldehyde. It turned out that the catalyst system derived from 6a was slightly less active than the published system using the ubiquitous TEMPO radical (see the ESI† for details).

## Conclusions

Boryl radical adducts of spin traps have been used to detect and characterize boron centred radicals.^5–8^ However, persistent boryl substituted nitroxide radicals that could be isolated and even characterized by X-ray crystal structure analysis are much less common.^9^ In this paper, we have described the preparation of a pair of boron substituted nitroxide radicals that were isolated and characterized spectroscopically and by X-ray crystal structure analysis. The compounds 5a and 6a were obtained in a unique way by an analogue of the nitroso ene reaction, namely the bora nitroso ene reaction of a reactive cyclic boraalkene with nitrosobenzene. The EPR spectra have revealed that the spin density is affected by the presence of the boryl substituent at nitrogen. There are sizable *A*(^11^B) coupling constants found for both the boryl nitroxide radicals 5a and 6a, and their *A*(^14^N) coupling constants are markedly lower than *e.g.* for TEMPO. However, this effect is too small to show up in the structural data. The N–O· bond lengths of 5a and 6a (1.304(2) Å and 1.289(2) Å) fall within the same range as those of P/B FLPNO radical IV (1.297(2) Å) and the ubiquitous TEMPO radical (1.284(2) Å). First experiments indicate that this type of B–NO radical shows the typical nitroxide radical chemical behaviour, and we shall see if the incorporated *N*-boryl unit might lead to an extension of their reaction patterns.

## Data availability

The ESI† contains experimental procedures, detailed analytical and structural data.

## Author contributions

C. C. designed and performed the experiments, C. C. and G. K. analysed the experimental data, C. G. D. performed the X-ray crystal structure analysis, S. K. and M. R. H. performed the EPR measurements and data analysis, and G. E. supervised the project and wrote the paper with input from all authors.

## Conflicts of interest

There are no conflicts to declare.

## Supplementary Material

SC-013-D2SC02485C-s001

SC-013-D2SC02485C-s002
