# Supplementary material for: Generation of boryl-nitroxide radicals from a boraalkene via the nitroso ene reaction
Source: Chem Sci. 2022 Aug 18;13(36):10891–6. doi: 10.1039/d2sc02485c (PMC9491209; doi:10.1039/d2sc02485c)
Supplement: SC-013-D2SC02485C-s001 [file SC-013-D2SC02485C-s001.pdf]

Electronic Supplementary Material (ESI) for Chemical Science.
This journal is © The Royal Society of Chemistry 2022

Electronic Supplementary Information

Generation of Boryl-nitroxide Radicals from a Boraalkene via the Nitroso Ene Reaction

Chaohuang Chen,[a] Constantin G. Daniliuc,[a] Sina Klabunde,[b] Michael Ryan Hansen,[b] Gerald Kehr,[a] and Gerhard Erker*[a]

[a]Organisch-Chemisches Institut, Westfälische Wilhelms-Universität Münster, Corrensstraße 40, 48149 Münster (Germany); [b] Institut für Physikalische Chemie, Westfälische Wilhelms-Universität Münster, Corrensstraße 28/30, 48149 Münster (Germany)

Contents

General information S2
1. Synthesis and characterization of compound **2** S4
2. Synthesis and characterization of compounds **4a**, **5a** and **6a** S10
3. Condition screening for the competitive formation of compounds **4a**, **5a** and **6a** S26
4. UV-vis spectra of compounds **5a** and **6a** S27
5. Cyclic voltamograms of compounds **5a** and **6a** S28
6. EPR spectra of compounds **5a** and **6a** S30
7. Synthesis and characterization of compound **4b** S33
8. Synthesis and characterization of compound **4c** S40
9. Synthesis and characterization of compound **9** S51
10. Generation of compound **5a** from compound **9** S57
11. Synthesis and characterization of compound **13** S58
12. Initial studies on (bpy)Cu^{I}/[B]NO• catalysed oxidation of alkyl alcohols S64
References S66

General information

All syntheses involving air- and moisture sensitive compounds were carried out using standard Schlenk-type glassware (or in a glove box) under an atmosphere of argon. Toluene, CH_2Cl_2, and pentane were dried using a Grubbs-type solvent purification system with alumina spheres as the drying agent. C_6H_5Br was dried with CaH_2 and stored with 4Å molecular sieves. All deuterated solvents (CD_2Cl_2, C_6D_6, toluene-d_8, C_6D_5Br) were dried with CaH_2 and stored with 4Å molecular sieves. All solvents were stored under an argon atmosphere. NMR spectra were recorded on a Varian Inova 600 (^{1}H: 600 MHz, ^{13}C{^{1}H}: 151 MHz, ^{31}P: 243 MHz, ^{19}F: 564 MHz, ^{11}B: 192 MHz). ^{1}H and ^{13}C NMR chemical shifts are given relative to tetramethylsilane (TMS) and referenced to the solvent signal. ^{31}P, ^{19}F and ^{11}B are referenced according to the proton resonance of TMS as the primary reference for the unified chemical shift scale (IUPAC recommendation 2001: R. K. Harris, E. D. Becker, S. M. Cabral De Menezes, R. Goodfellow, P. Granger, *Pure Appl. Chem.* 2001, **73**, 1795). NMR assignments were supported by additional 1D (NOESY and TOCSY) and 2D (gCOSY, gHSQC and gHMBC) NMR experiments. Elemental analysis data was recorded on a Foss Heraeus CHNO-Rapid machine or an Elementar UNICUBE machine. Melting points and decomposition points were obtained with a DSC 2010 (TA-instruments). HRMS was recorded using a Thermo Scientific Orbitrap LTQ XL machine.

X-Ray diffraction: Data sets for compounds **4c$_{ax}$**, **5a**, **6a**, **8** and **11** were collected with a Bruker D8 Venture Photon III Diffractometer. Data sets for compounds **2** and **4a** were collected with a Bruker APEX II CCD Diffractometer. Programs used: data collection: APEX3 V2019.1-0[1] (Bruker AXS Inc., **2019**); cell refinement: SAINT V8.40A (Bruker AXS Inc., **2019**); data reduction: SAINT V8.40A (Bruker AXS Inc., **2019**); absorption correction, SADABS V2016/2 (Bruker AXS Inc., **2019**); structure solution *SHELXT-2015*[2] (Sheldrick, G. M. *Acta Cryst.,* **2015**, *A71*, 3-8); structure refinement *SHELXL-2015*[3] (Sheldrick, G. M. *Acta Cryst.,* **2015**, *C71* (1), 3-8) and graphics, *XP*[4] (Version 5.1, Bruker AXS Inc., Madison, Wisconsin, USA, **1998**). *R*-values are given for observed reflections, and wR^2 values are given for all reflections. *Exceptions and special features*: For compound **6a** one C_6F_5 group was found disordered over two positions in the asymmetric unit. Several restraints (SADI, SAME, ISOR and SIMU) were used in order to improve refinement stability. For compound **4c** a badly disordered half heptane molecule was found in the asymmetrical unit and could not be satisfactorily refined. The program SQUEEZE (Spek, A.L. (**2015**). Acta Cryst. C71, 9-18) was therefore used to remove mathematically the effect of the solvent. The quoted formula and derived parameters are not included the squeezed solvent molecule.

Materials: Unless otherwise noted, all chemicals were purchased from commercially available sources and used as received. Compounds **1** and **3** were prepared according to procedures descried in the literature.[5]

1. Synthesis and characterization of compound 2

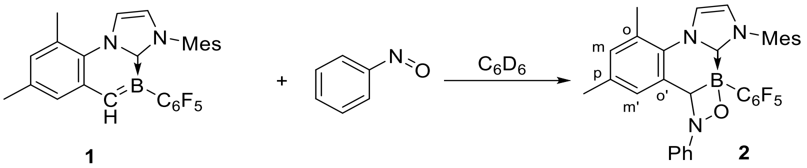

In a Young NMR tube, compound **1** (9.6 mg, 0.02 mmol, 1.0 equiv.) and PhNO (2.2 mg, 0.02 mmol, 1.0 equiv.) were mixed and C_6D_6 (0.6 mL) was added. After the resulting mixture was shaken at r.t. for 5 min., it was dried in vacuo. The remaining residue was washed with pentane (3 × 1 mL) and dried in vacuo, to give compound **2** as a pale-white solid (10.5 mg, 89%). Crystals suitable for the X-ray single crystal structure analysis of compound **2** were obtained from a solution of the obtained pale-white solid in C_6D_6/pentane (v/v < 1:5) at r.t.

Elemental analysis (%) calc. for $C_{33}H_{27}BF_5N_3O$: C, 67.48; H, 4.63; N, 7.15. Found: C, 67.03; H, 4.76; N, 7.22.

NMR data of compound **2** were obtained from a solution of the obtained pale-white solid in C_6D_6 at r.t.

^{1}H NMR (600 MHz, 299 K, C_6D_6) δ [7.56 (m, 2H, *o*-), 7.24 (m, 2H, *m*-), 6.84 (m, 1H, *p*-)](Ph), [7.19 (*m'*-), 6.70 (*m*-)](each m, each 1H, C_6H_2), [6.86, 6.40](each m, each 1H, *m*-Mes), [6.84, 5.94](each d, $^3J_{HH}$ = 1.8 Hz, each 1H, =CH), 4.57 (s, 1H, HCB), [2.41, 1.17](each s, each 3H, *o*-Me^{Mes}), 2.12 (s, 3H, *p*-Me^{Mes}), 2.05 (s, 3H, *p*-Me^{C6H2}), 2.00 (s, 3H, *o*-Me^{C6H2}).

^{13}C{^{1}H} NMR (151 MHz, 299 K, C_6D_6) δ 165.6 (br, BC), [160.1 (*i*-), 128.7 (*m*-), 120.9 (*p*-), 114.6 (*o*-)](Ph), 140.4 (*p*-Mes), 137.8 (*p*-C_6H_2), [137.3, 134.1](*o*-Mes), [134.5, 127.4 (*o*-)](C_6H_2), 133.4 (*i*-C_6H_2), [133.0 (*m*-), 131.4 (*m'*-)](C_6H_2), 132.8 (*i*-Mes), [130.3, 128.0](*m*-Mes), [120.4, 120.3](=CH), 69.1 (br, HCB), 21.9 (*o*-Me^{C6H2}), 20.9 (*p*-Me^{Mes}), 20.6 (*p*-Me^{C6H2}), [18.3, 16.0](*o*-Me^{Mes}), [C_6F_5 not listed].

^{11}B{^{1}H} NMR (192 MHz, 299 K, C_6D_6) δ -4.1 ($\nu_{1/2} \approx 90$ Hz).

^{19}F NMR (564 MHz, 299 K, C_6D_6) δ -134.6 (br, 2F, *o*-C_6F_5), -160.1 (t, $^3J_{FF}$ = 20.3 Hz, 1F, *p*-C_6F_5), -165.4 (br, 2F, *m*-C_6F_5), [$\Delta\delta^{19}F_{m,p}$ = 5.3].

^{1}H,^{13}C gHMBC (600 MHz/151 MHz, 299 K, C_6D_6)[selected traces] δ ^{1}H/^{13}C [7.19/133.4, 133.0, 69.1, 20.6](*m*-C_6H_2/*i*-C_6H_2, *m*-C_6H_2, HCB, *p*-Me^{C6H2}), [(6.84/165.6, 120.4), (5.94/165.6, 120.3)](=CH/BC, =CH), [4.57/165.6, 160.1, 133.4, 131.4, 118.5](HCB/BC, *i*-Ph, *i*-C_6H_2, *m'*-C_6H_2, *i*-C_6F_5).

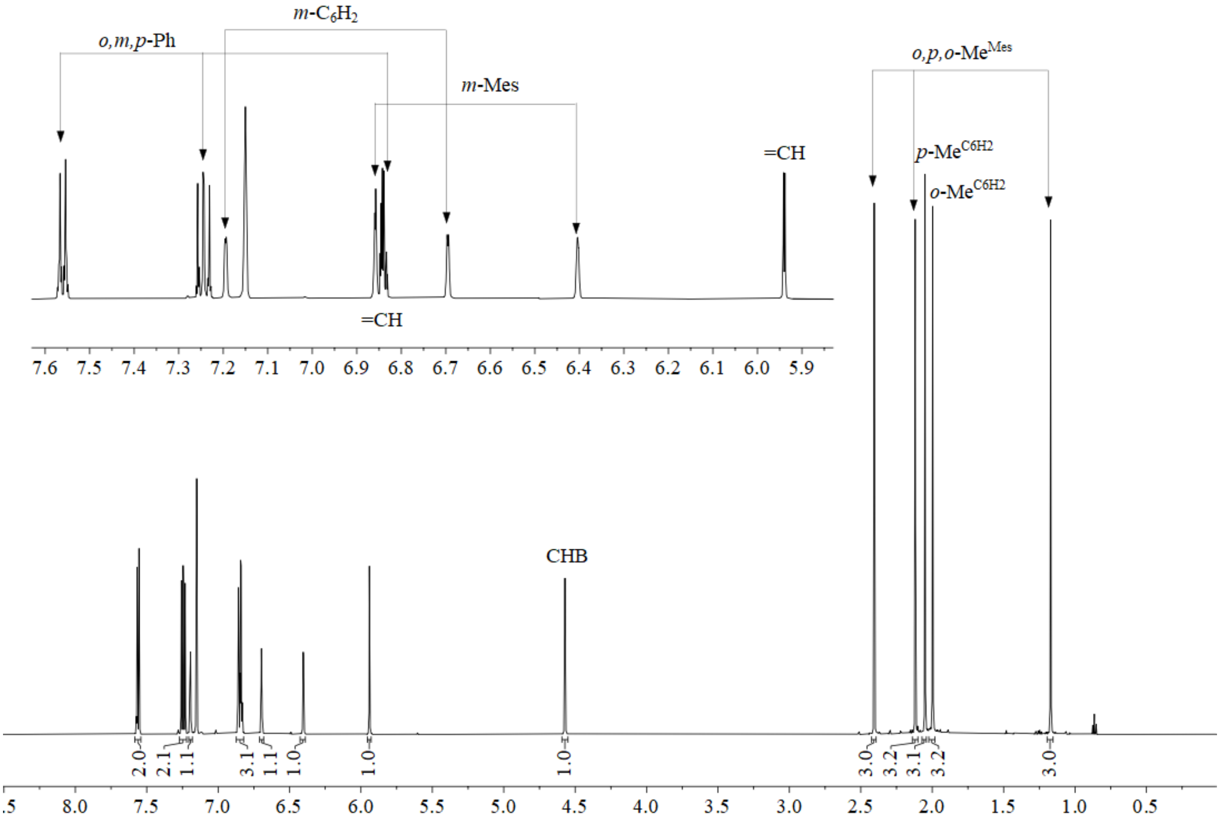

Figure S1. ^{1}H NMR (600 MHz, 299 K, C_6D_6) spectrum of compound **2**

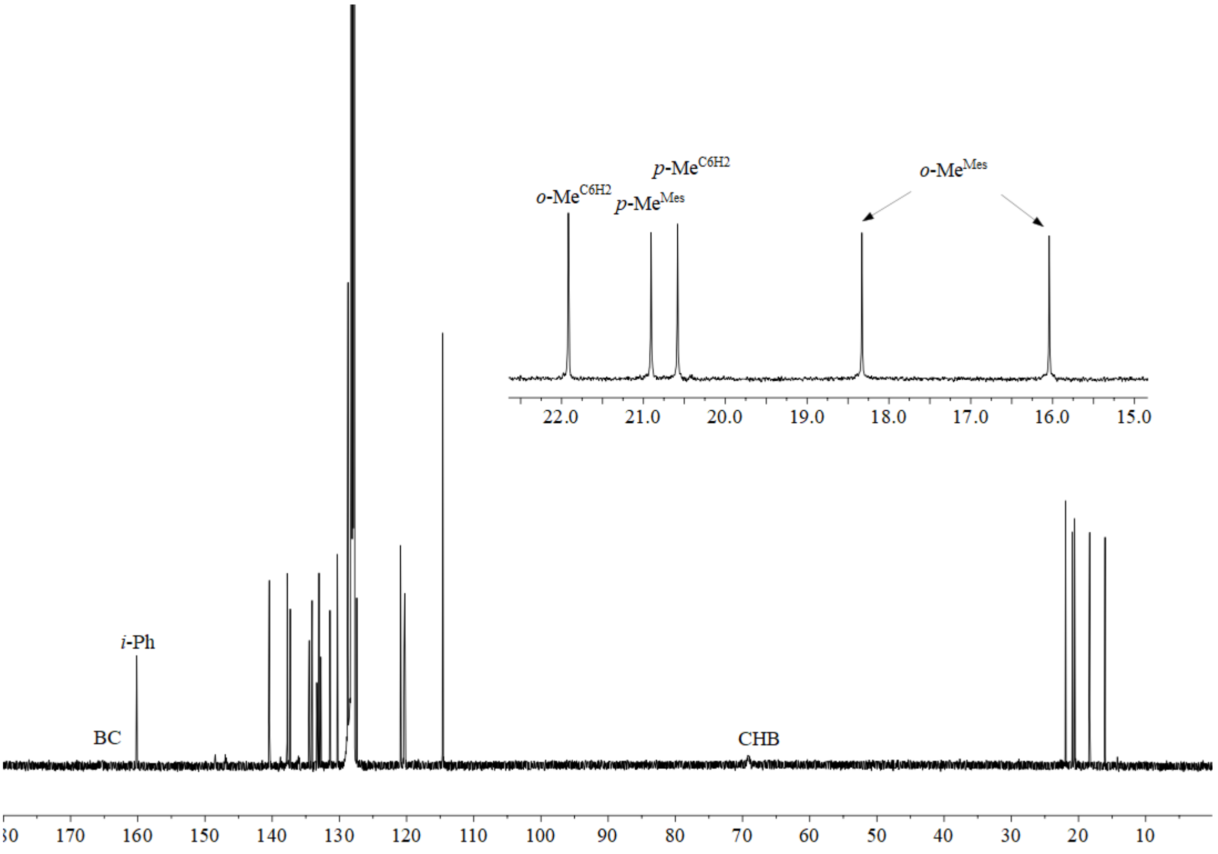

Figure S2a. $^{13}C\{^1H\}$ NMR (151 MHz, 299 K, C_6D_6) spectrum of compound **2**

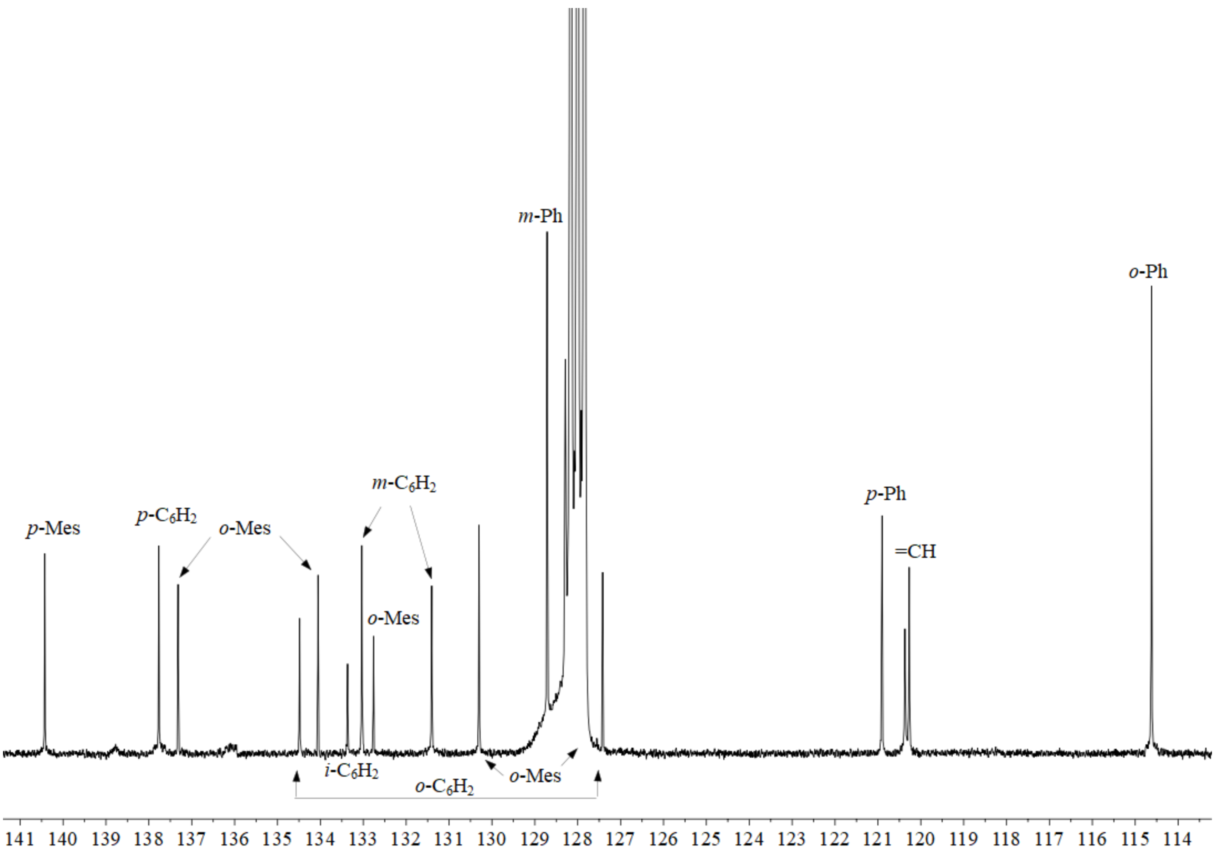

Figure S2b. $^{13}C\{^1H\}$ NMR (151 MHz, 299 K, C_6D_6) spectrum of compound **2**

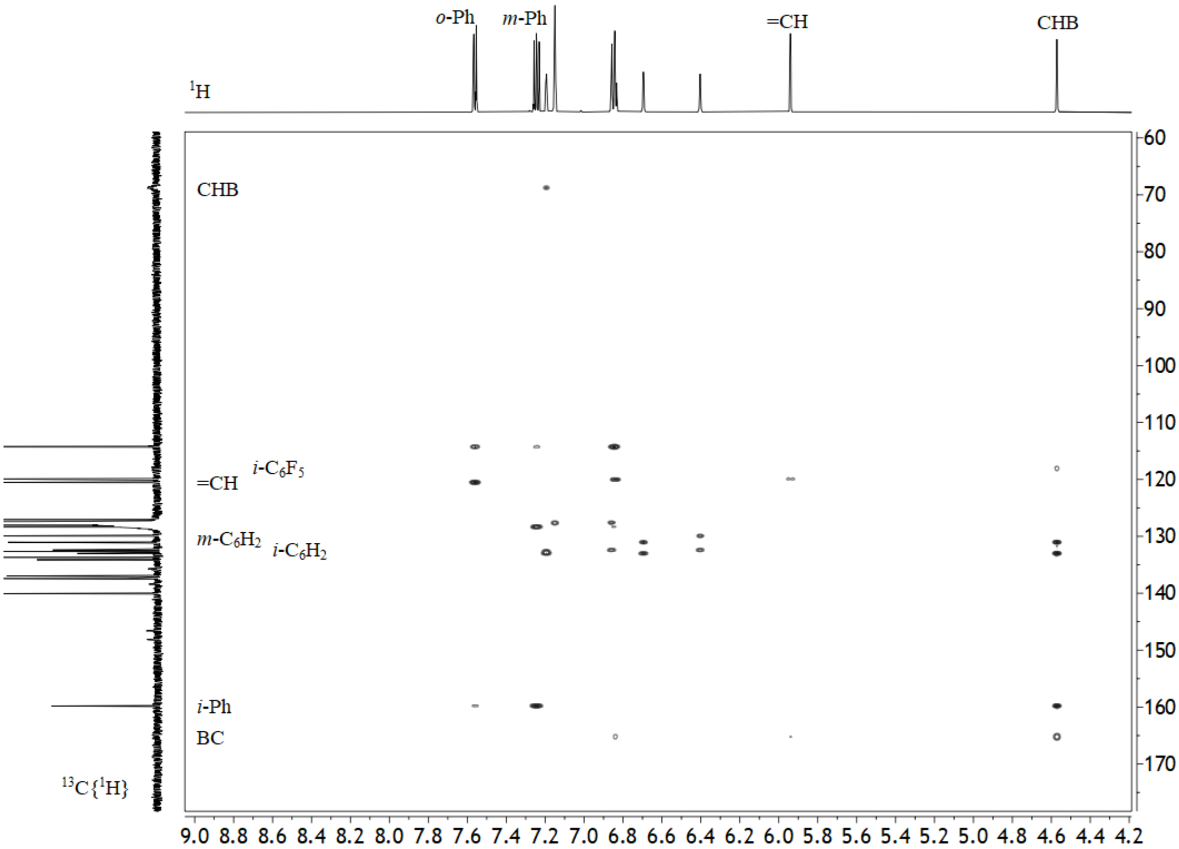

Figure S3. $^1H,^{13}C$ gHMBC (600/151 MHz, 299 K, C_6D_6) spectrum of compound **2**

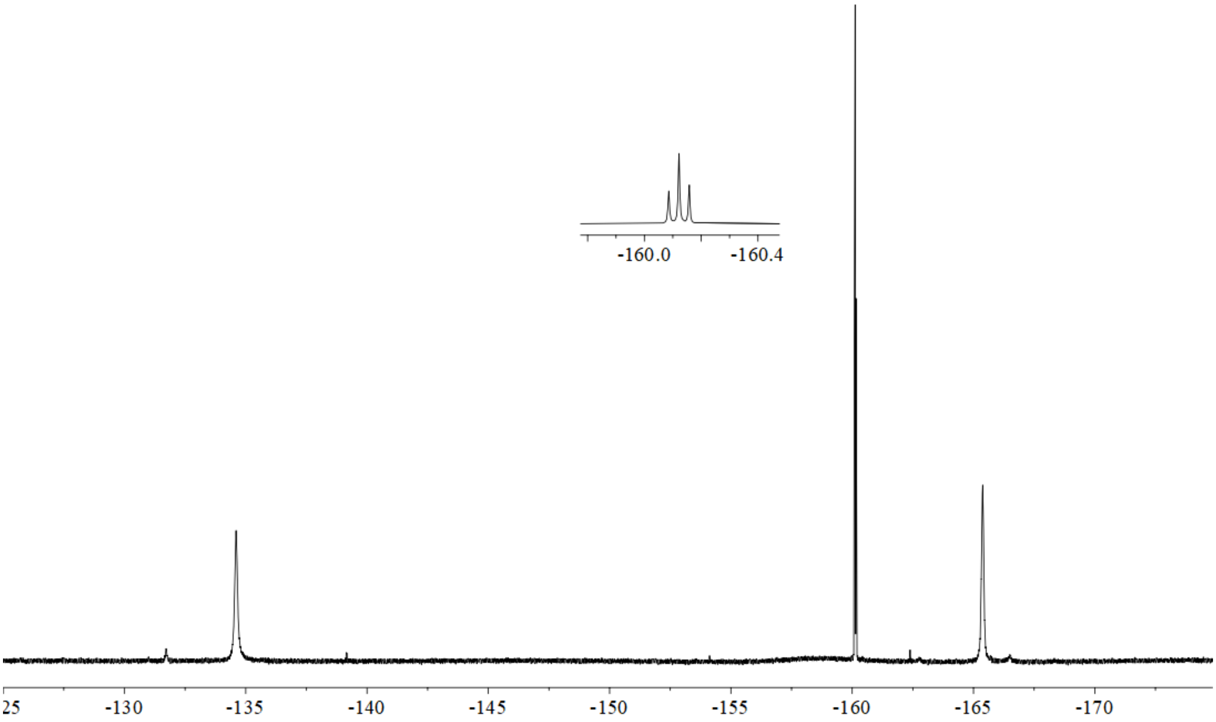

Figure S4. ^{19}F NMR (564 MHz, 299 K, C_6D_6) spectrum of compound **2**

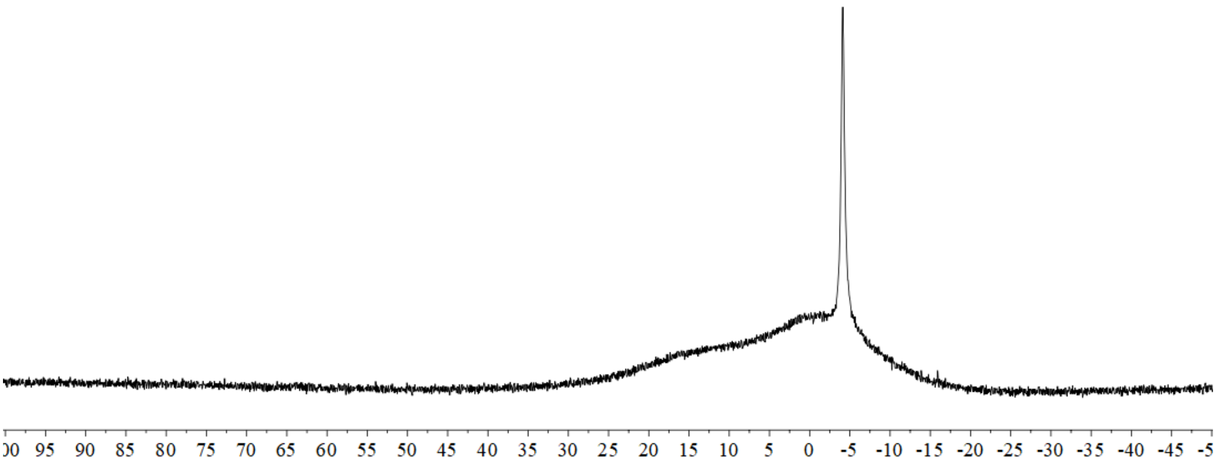

Figure S5. ^{11}B{^{1}H} NMR (192 MHz, 299 K, C_6D_6) spectrum of compound **2**

X-ray crystal structure analysis of compound 2 (ERK10103): A colorless plate-like specimen of $C_{33}H_{27}BF_5N_3O$, approximate dimensions 0.040 mm x 0.100 mm x 0.140 mm, was used for the X-ray crystallographic analysis. The X-ray intensity data were measured on a Kappa CCD APEXII Bruker APEXII Diffractometer system equipped with a fine-focus sealed tube Cu sealed tube (CuKα, λ = 1.54178 Å) and a graphite monochromator. A total of 1649 frames were collected. The total exposure time was 18.69 hours. The frames were integrated with the Bruker SAINT software package using a wide-frame algorithm. The integration of the data using a monoclinic unit cell yielded a total of 35334 reflections to a maximum θ angle of 66.77° (0.84 Å resolution), of which 5118 were independent (average redundancy 6.904, completeness = 98.5%, R_{int} = 10.34%, R_{sig} = 5.99%) and 3320 (64.87%) were greater than 2σ(F^2). The final cell constants of a = 8.5951(4) Å, b = 13.5070(7) Å, c = 25.5281(10) Å, β = 99.612(3)°, volume = 2922.1(2) $Å^3$, are based upon the refinement of the XYZ-centroids of 3651 reflections above 20 σ(I) with 7.024° < 2θ < 131.3°. Data were corrected for absorption effects using the Multi-Scan method (SADABS). The ratio of minimum to maximum apparent transmission was 0.864. The calculated minimum and maximum transmission coefficients (based on crystal size) are 0.8880 and 0.9660. The structure was solved and refined using the Bruker SHELXTL Software Package, using the space group $P2_1/c$, with Z = 4 for the formula unit, $C_{33}H_{27}BF_5N_3O$. The final anisotropic full-matrix least-squares refinement on F^2 with 394 variables converged at R1 = 5.21%, for the observed data and wR2 = 14.53% for all data. The goodness-of-fit was 1.026. The largest peak in the final difference electron density synthesis was 0.307 $e^-/Å^3$ and the largest hole was -0.270 $e^-/Å^3$ with an RMS deviation of 0.068 $e^-/Å^3$. On the basis of the final model, the calculated density was 1.335 g/cm^3 and F(000), 1216 e^-. CCDC Nr.: 2157861.

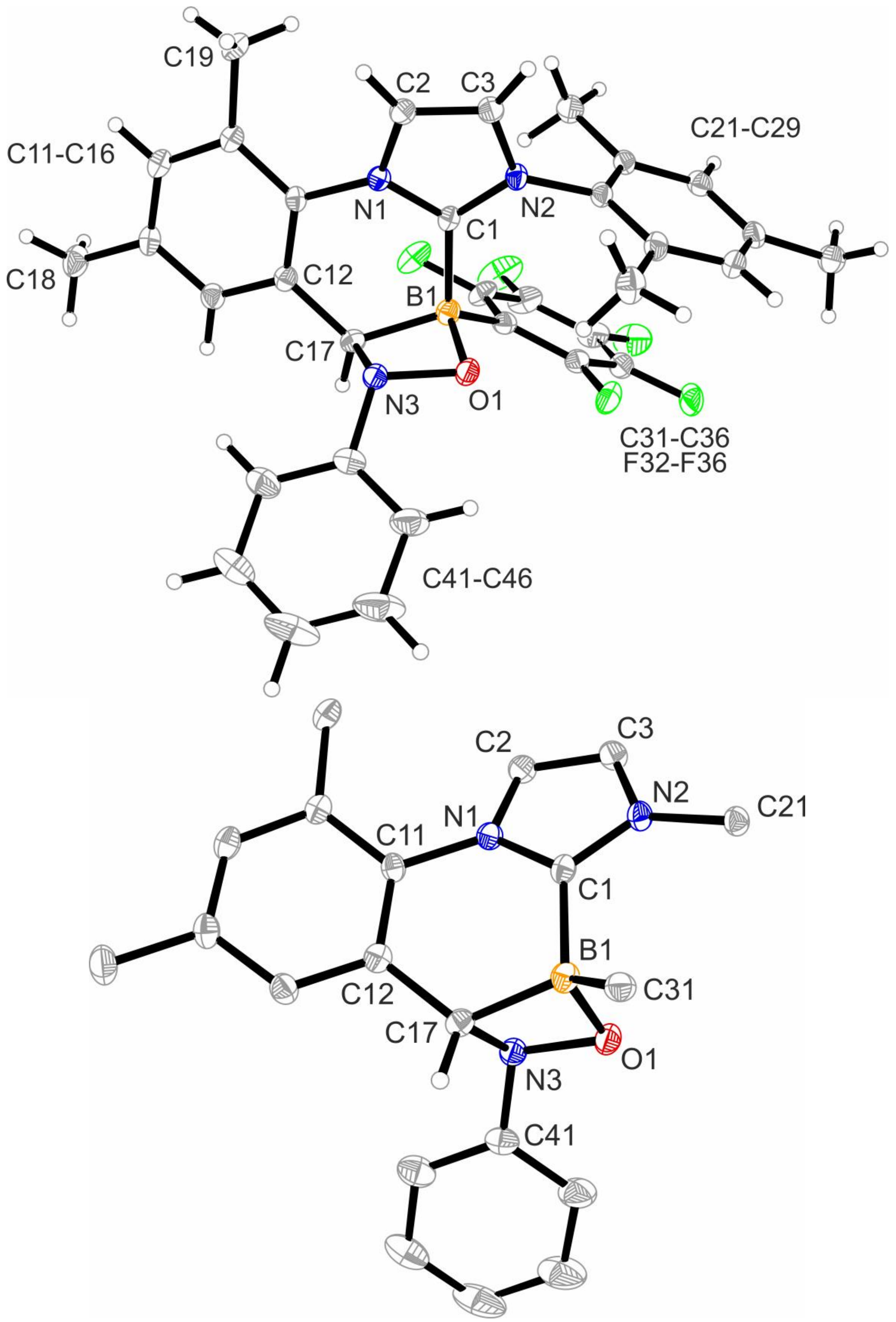

Figure S6. Crystal structure of compound **2** (thermal ellipsoids: 30% probability).

2. Synthesis and characterization of compounds 4a, 5a and 6a

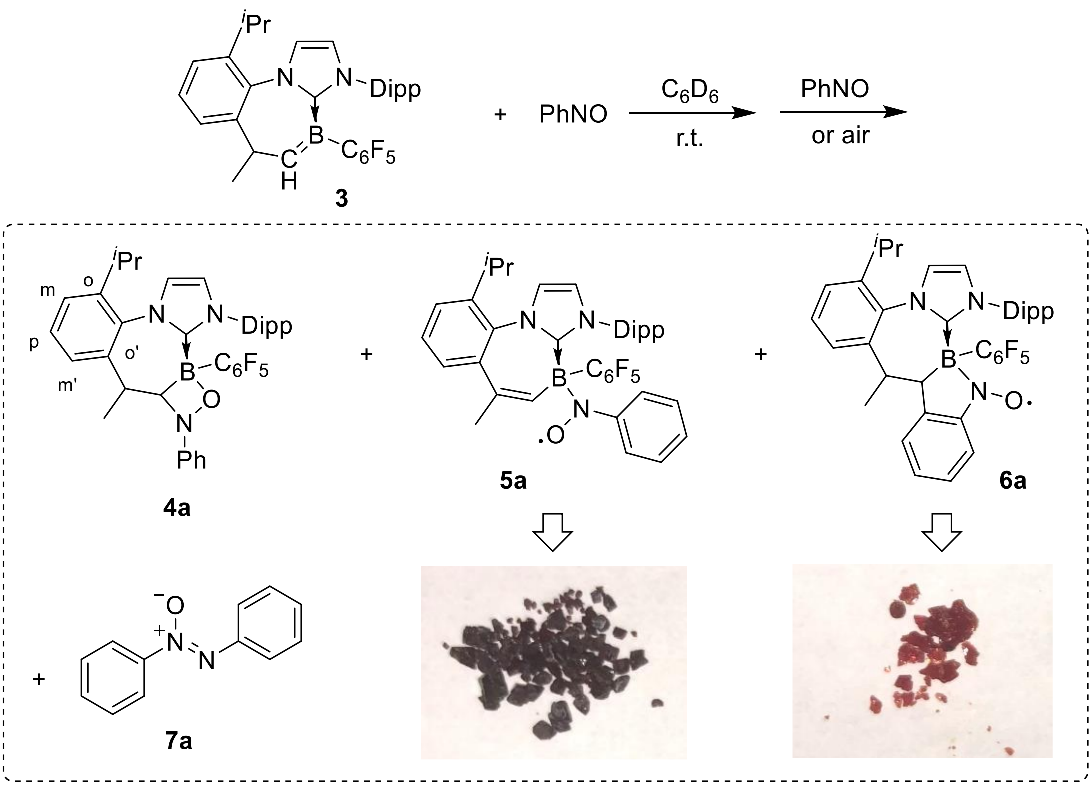

1st Experiment: Compound **3** (28.3 mg, 0.05 mmol, 1.0 equiv.) and PhNO (10.7 mg, 0.1 mmol, 2.0 equiv.) were mixed and C_6D_6 (0.5 mL) was added. The resulting mixture was stirred at r.t. for 1h and directly used for ^{1}H, ^{19}F, and ^{11}B NMR experiments (Figure S7-S9).

2nd Experiment: Compound **3** (112.6 mg, 0.2 mmol, 1.0 equiv.) and PhNO (42.8 mg, 0.4 mmol, 2.0 equiv.) were mixed and C_6D_6 (1.5 mL) was added. The resulting mixture was stirred at r.t. for 1h. Then the mixture was purified by flash chromatograph (SiO_2, eluent: pentane:ethyl acetate from 10:1 to 5:1) to give: (1) compound **4a** as an off-white solid (37.6 mg, 28%), (2) compound **5a** as a brown solid (46.9 mg, 35%), (3) compound **6a** as a yellow solid (23.7 mg, 18%), and (4) compound **7a** as a light-yellow oil (ca. 9.4 mg, 50%).

3rd Experiment: Compound **3** (56.3 mg, 0.1 mmol, 1.0 equiv.) and PhNO (21.4 mg, 0.2 mmol, 1.0 equiv.) were mixed and C_6D_6 (1.0 mL) was added. The mixture was stirred at r.t. for 10 min and then exposed to the air. The reaction was stirred at r.t. overnight and the obtained mixture was purified by flash chromatograph (SiO_2, eluent: pentane:ethyl acetate from 10:1 to 5:1) to give: (1) compound **4a** as an off-white solid (22.1 mg, 33%), (2) compound **5a** as a brown solid (27.2 mg, 41%), and (3) compound **6a** as a yellow solid (9 mg, 14%).

Crystallization of compounds **4a**, **5a**, and **6a**:

(1) Colorless crystals suitable for the X-ray single crystal structure analysis of compound **4a** were obtained from a solution of the obtained off-white solid in ethyl acetate at r.t.

(2) Deeply red crystals suitable for the X-ray single crystal structure analysis of compound **5a** were obtained from a solution of the obtained brown solid in CH_2Cl_2/pentane at r.t.

(3) Orange to red crystals suitable for the X-ray single crystal structure analysis of compound **6a** were obtained from a solution of the obtained brown solid in CH_2Cl_2/pentane at r.t.

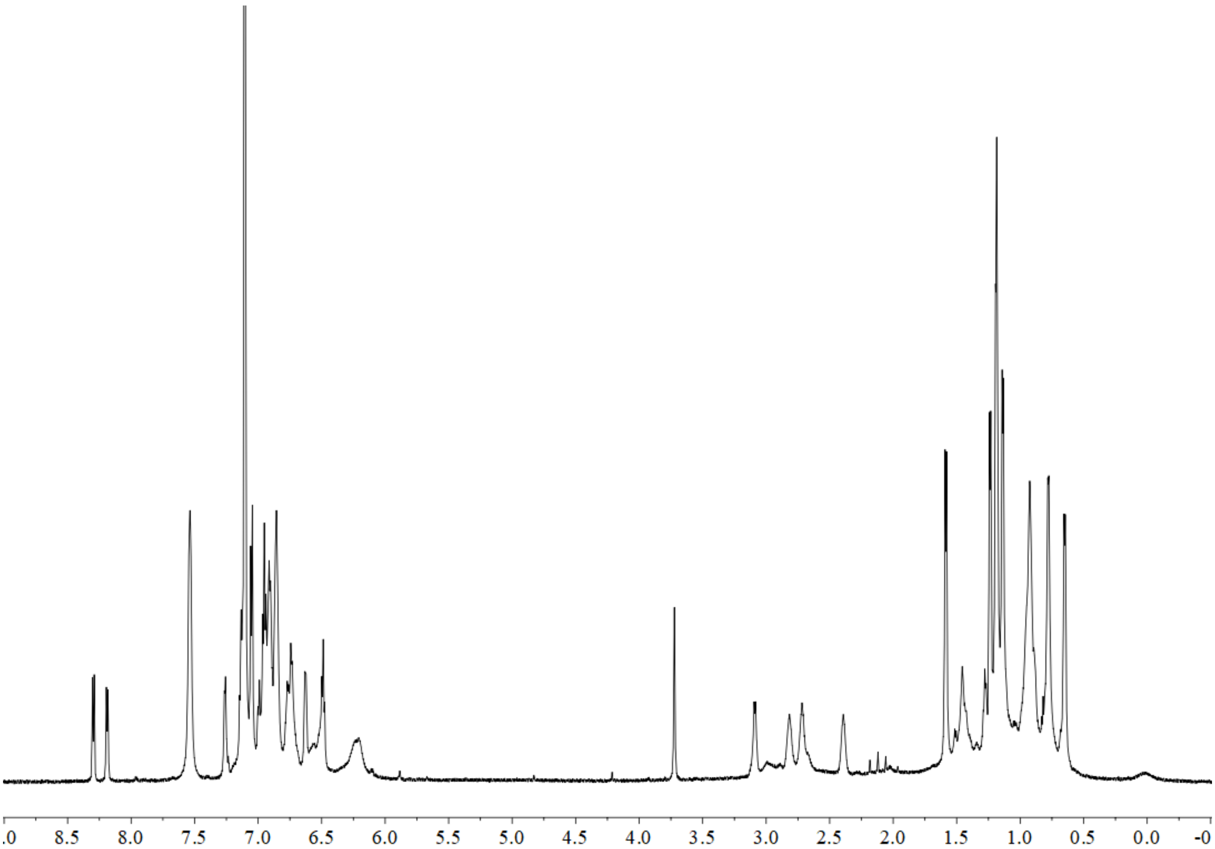

Figure S7. ^{1}H NMR (600 MHz, 299 K, C_6D_6) spectrum of the crude reaction mixture [**1st experiment**]

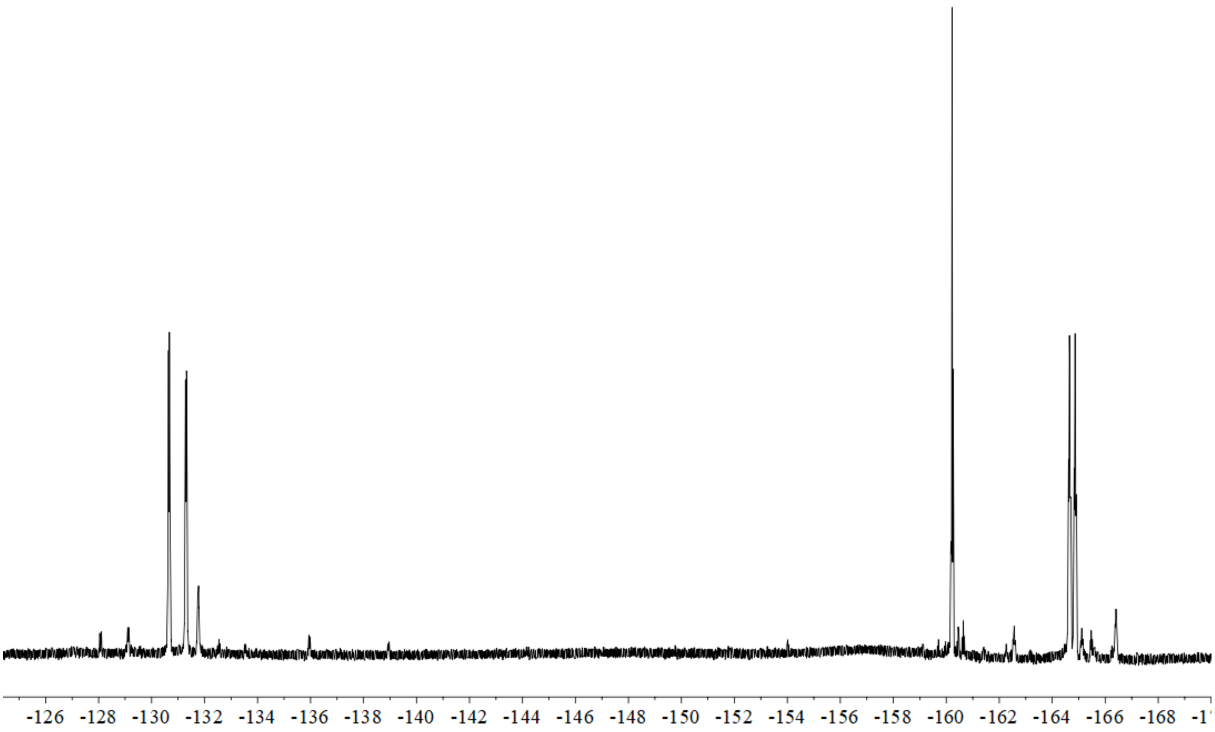

Figure S8. ^{19}F NMR (564 MHz, 299 K, C_6D_6) spectrum of the crude reaction mixture [**1st experiment**]

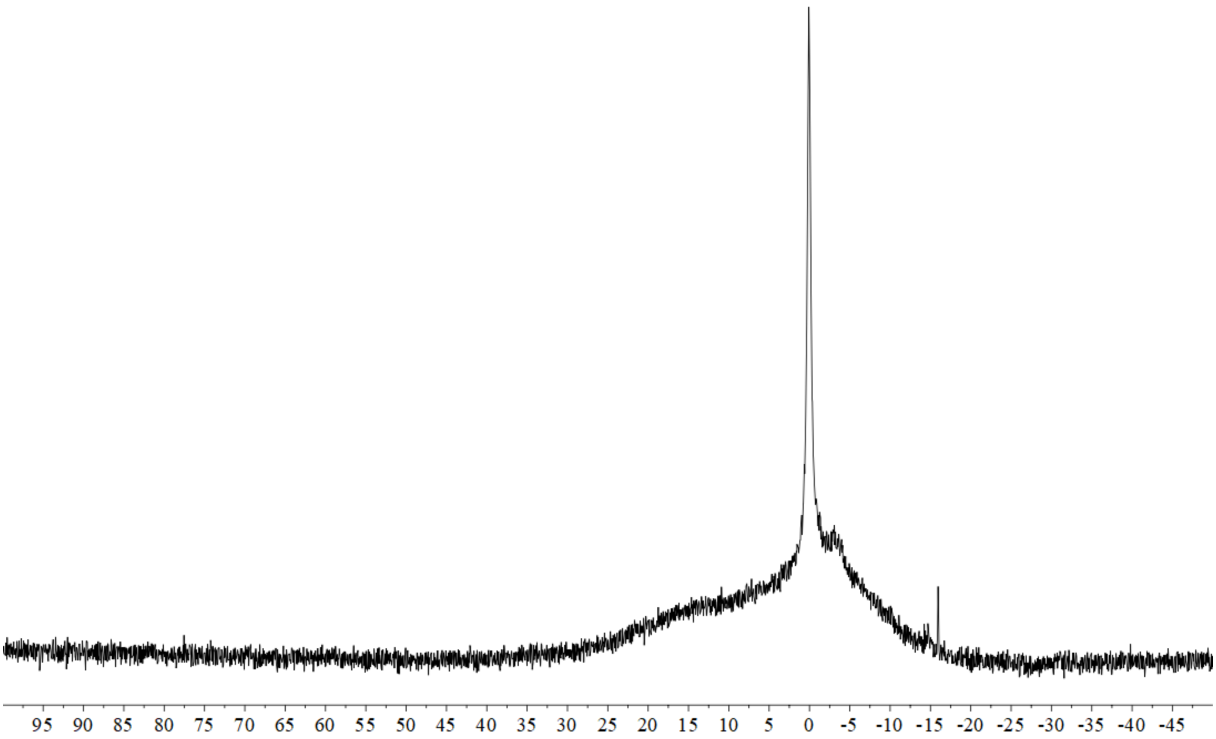

Figure S9. $^{11}B\{^1H\}$ NMR (192 MHz, 299 K, C_6D_6) spectrum of the crude reaction mixture [**1st experiment**]

Characterization of compound 4a (2nd Experiment):

Elemental analysis (%) calc. for $C_{39}H_{39}BF_5N_3O$: C, 69.75; H, 5.85; N, 6.26. Found: C, 69.37; H, 5.83; N, 6.58.

NMR data of compound **4a** were obtained from a solution of the obtained white solid in CD_2Cl_2 at r.t. [Dipp: 2,6-diisopropylphenyl]

^{1}**H NMR** (600 MHz, 299 K, CD_2Cl_2) δ [7.47 (m, *p*-), 7.46 (m, *m*-), 7.37 (ddm, $^3J_{HH}$ = 6.7 Hz, *J* = 2.5 Hz, *m'*-)](each 1H, C_6H_3), [7.36, 7.20] (each d, $^3J_{HH}$ = 1.8 Hz, each 1H, =CH), {7.23 (t, $^3J_{HH}$ = 7.8 Hz, 1H, *p*-), [7.11, 6.98](each dd, $^3J_{HH}$ = 7.8 Hz, $^4J_{HH}$ = 1.4 Hz, each 1H, *m*-)}(Dipp), [6.91 (m, 2H, *m*-), 6.60 (m, 2H, *o*-), 6.51 (m, 1H, *p*-)](Ph), 3.44 (d, *J* = 3.0 Hz, HCB), {3.13 (sept, $^3J_{HH}$ = 6.8 Hz, 1H), [1.48, 1.22](each d, $^3J_{HH}$ = 6.8 Hz, each 3H)}($i\mathrm{Pr}^{C6H3}$), 3.05 (q, $^3J_{HH}$ = 7.1 Hz, 1H, CH^{CHB}), [2.66 (sept, $^3J_{HH}$ = 6.7 Hz, 1H), 1.25 (dd, $^3J_{HH}$ = 6.7 Hz, *J* ≈ 0.7 Hz, 3H), 0.97 (d, $^3J_{HH}$ = 6.7 Hz, 3H)]($i\mathrm{Pr}^{Dipp}$), [2.46 (sept, $^3J_{HH}$ = 6.7 Hz, 1H), 1.32 (dd, $^3J_{HH}$ = 6.7 Hz, *J* ≈ 1.1 Hz, 1H), 1.09 (d, $^3J_{HH}$ = 6.7 Hz, 1H)]($i\mathrm{Pr}^{Dipp}$), 1.62 (d, $^3J_{HH}$ = 7.1 Hz, 3H, Me^{CHC6H3}).

^{13}**C{^{1}H} NMR** (151 MHz, 299 K, CD_2Cl_2) δ 164.0 (br, BC), [157.4 (*i*-), 128.0 (*m*-), 118.5 (*p*-), 112.8 (*o*-)](Ph), [145.7, 145.5](*o*-Dipp), [141.9 (*o*-), 140.7 (*o'*-)](C_6H_3), 135.0 (*i*-C_6H_3), 133.3 (*i*-Dipp), 130.8 (*p*-Dipp), 129.2 (*p*-C_6H_3), [125.6 (*m'*-), 124.8 (*m*-)](C_6H_3), [125.1, 124.4](=CH), [124.1, 123.4](*m*-Dipp), 80.7 (br, HCB), 38.1 (CH^{CHB}), 29.5 (*o*-CH^{iPr}(C_6H_3)), [29.2 (m, CH), 26.3 (Me), 21.1 (d, *J* = 3.8 Hz, Me)]($i\mathrm{Pr}^{Dipp}$), [29.1 (CH), 27.2 (Me), 21.6 (d, *J* = 7.3 Hz, Me)]($i\mathrm{Pr}^{Dipp}$), [24.5, 22.4](*o*-Me^{iPr}(C_6H_3)), 19.5 (Me^{CHC6H3}), [C_6F_5 not listed].

^{11}**B{^{1}H} NMR** (192 MHz, 299 K, CD_2Cl_2) δ -0.7 ($\nu_{1/2}$ ≈ 55 Hz).

^{19}**F NMR** (564 MHz, 299 K, CD_2Cl_2) δ [-130.8, -131.1](each m, each 1F, *o*-C_6F_5), -161.2 (t, $^3J_{FF}$ = 20.1 Hz, *p*-C_6F_5), [-165.2, -165.9](each m, each 1F, *m*-C_6F_5), [$\Delta\delta^{19}\mathrm{F}_{m,p}$ = 4.0, 4.7].

^{1}**H,**^{13}**C gHMBC** (600 MHz/151 MHz, 299 K, CD_2Cl_2)[selected traces] δ ^{1}H/^{13}C {[7.36/164.0, 125.1], [7.20/164.0, 124.4]}(=CH/BC, =CH), [3.44/164.0, 157.4, 140.7, 121.3, 19.5](HCB/BC, *i*-Ph, *o'*-C_6H_3, *i*-C_6F_5, Me^{CHC6H3}), [3.05/140.7, 135.0, 125.6, 19.5](CH^{CHB}/*o'*-C_6H_3, *i*-C_6H_3, *m'*-C_6H_3, Me^{CHC6H3}).

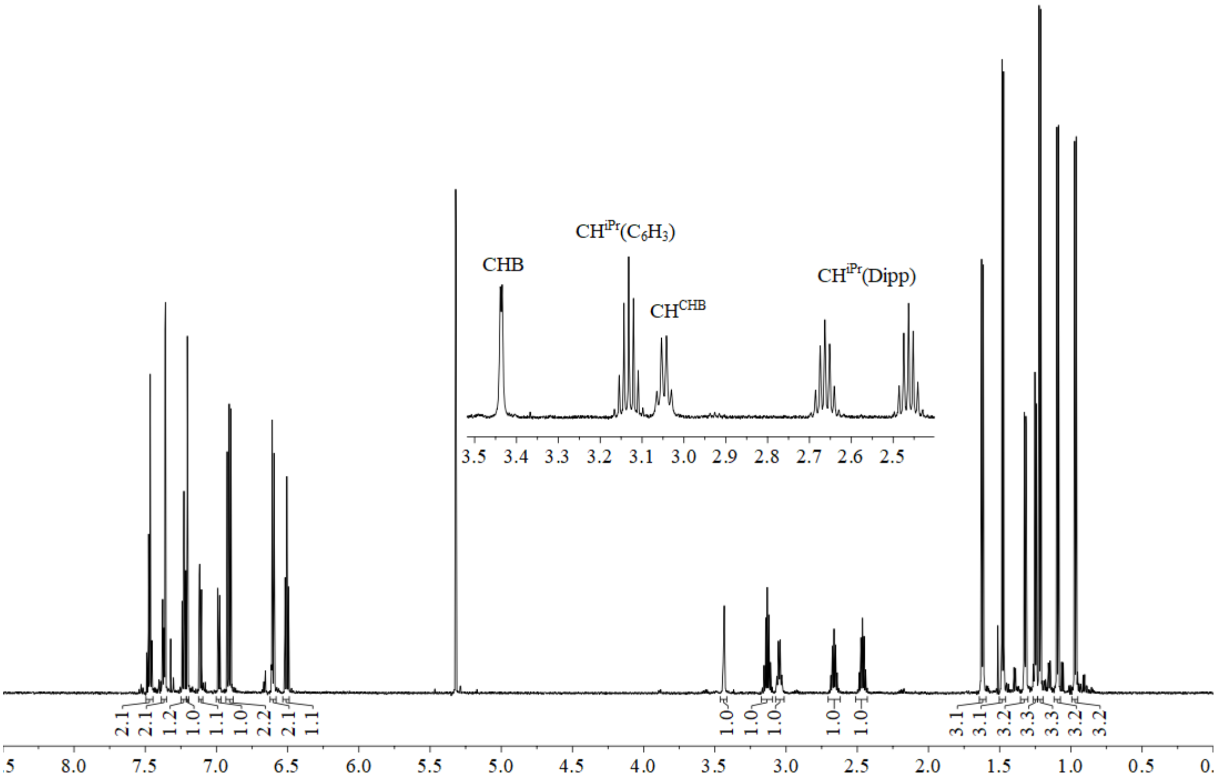

Figure S10a. ^{1}H NMR (600 MHz, 299 K, CD_2Cl_2) spectrum of compound **4a**

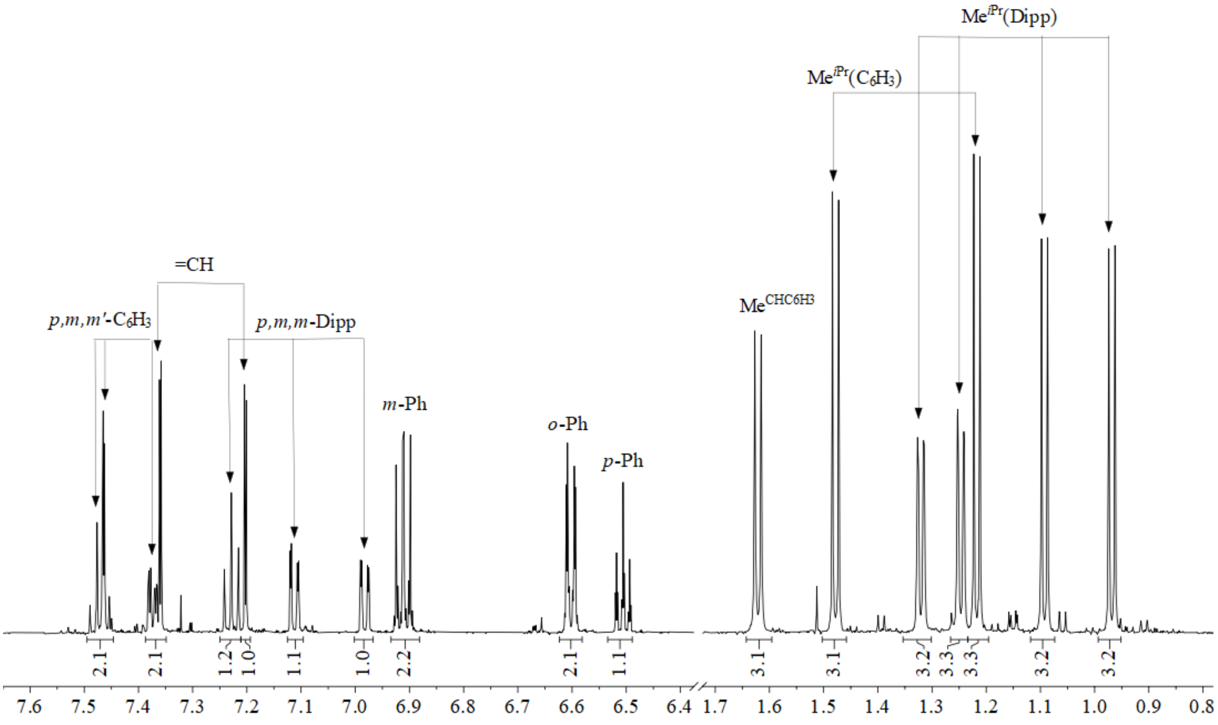

Figure S10b. ^{1}H NMR (600 MHz, 299 K, CD_2Cl_2) spectrum of compound **4a**

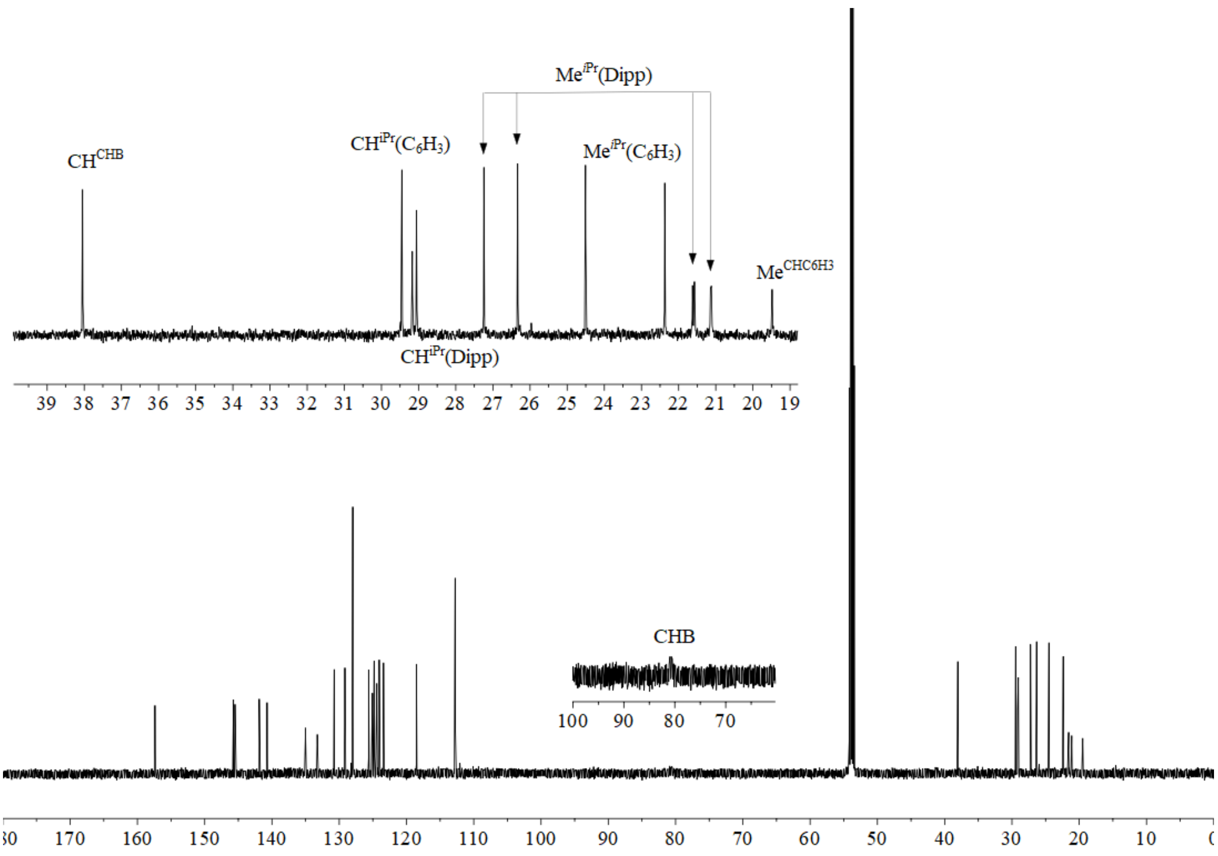

Figure S11a. $^{13}C\{^1H\}$ NMR (151 MHz, 299 K, CD_2Cl_2) spectrum of compound **4a**

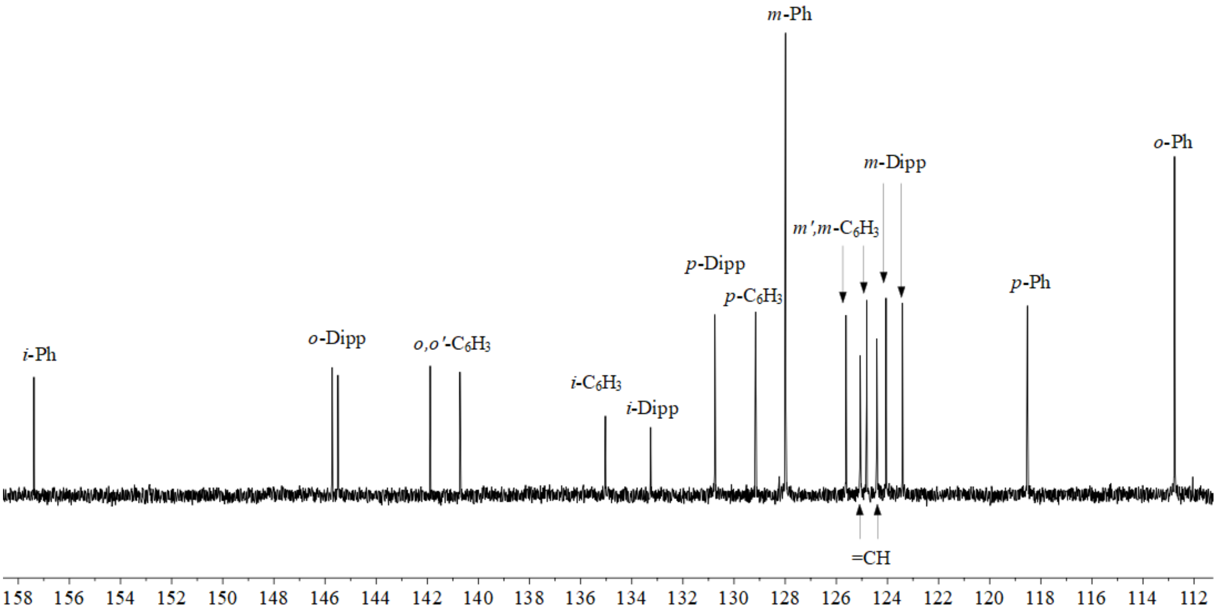

Figure S11b. $^{13}C\{^1H\}$ NMR (151 MHz, 299 K, CD_2Cl_2) spectrum of compound **4a**

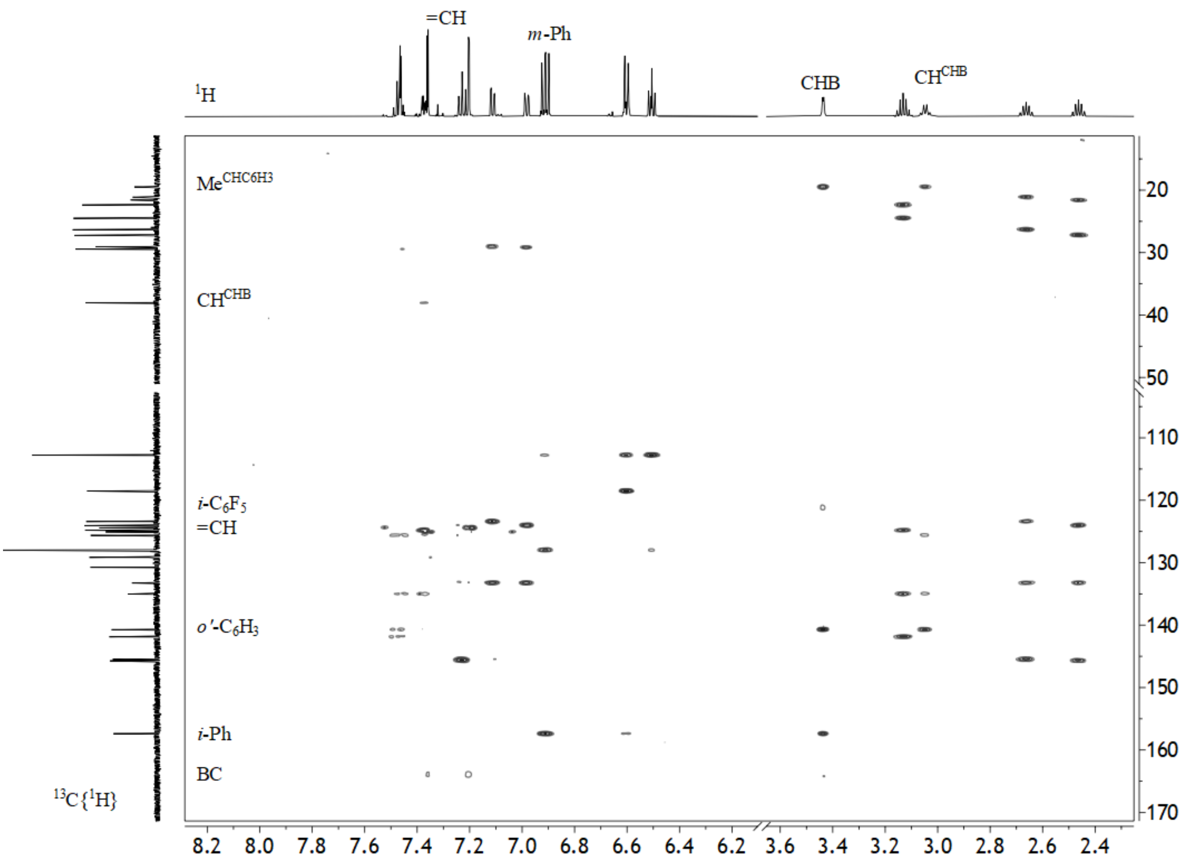

Figure S12. ^{1}H,^{13}C gHMBC (600/151 MHz, 299 K, CD_2Cl_2) spectrum of compound **4a**

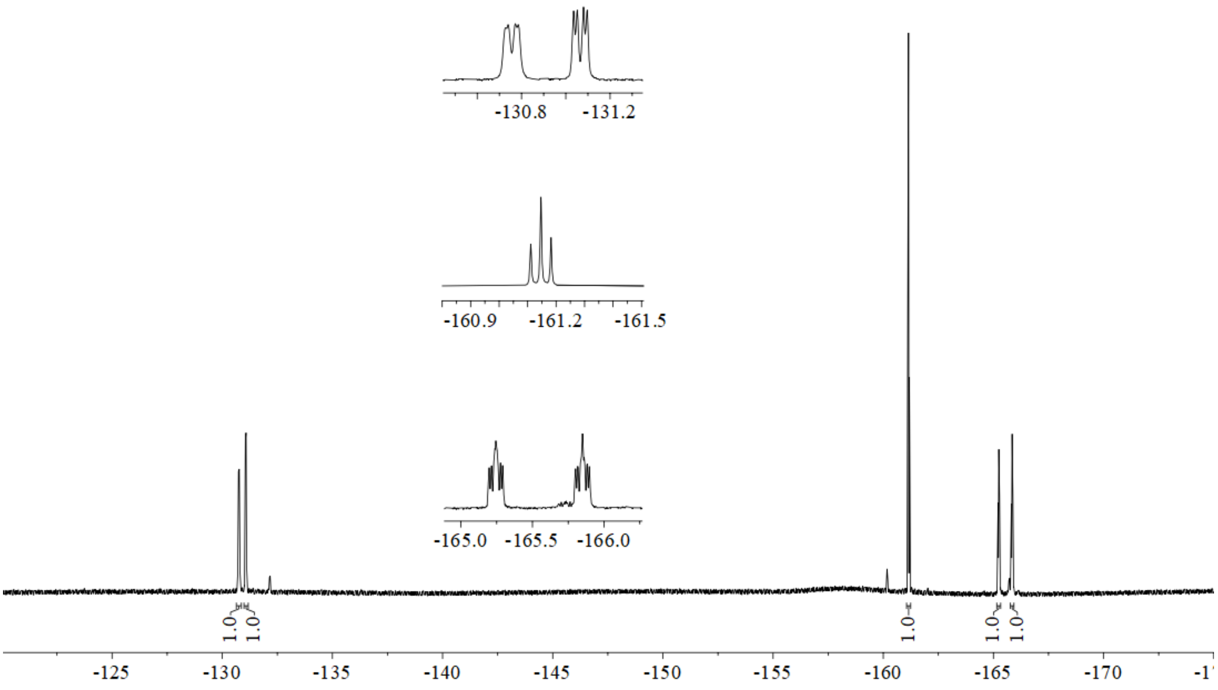

Figure S13. ^{19}F NMR (564 MHz, 299 K, CD_2Cl_2) spectrum of compound **4a**

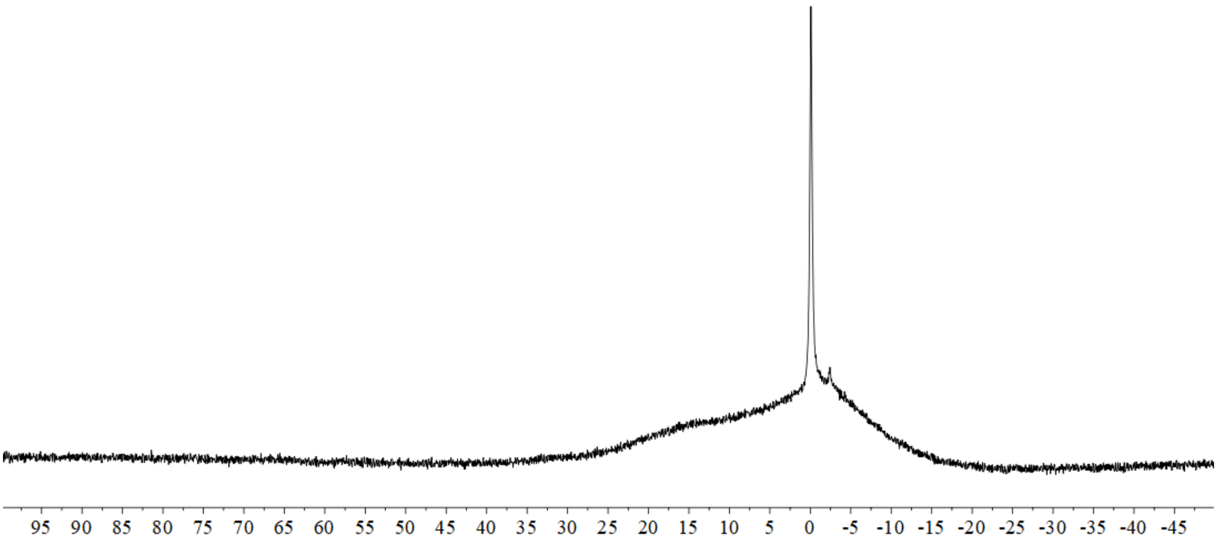

Figure S14. $^{11}B\{^{1}H\}$ NMR (192 MHz, 299 K, CD_2Cl_2) spectrum of compound **4a**

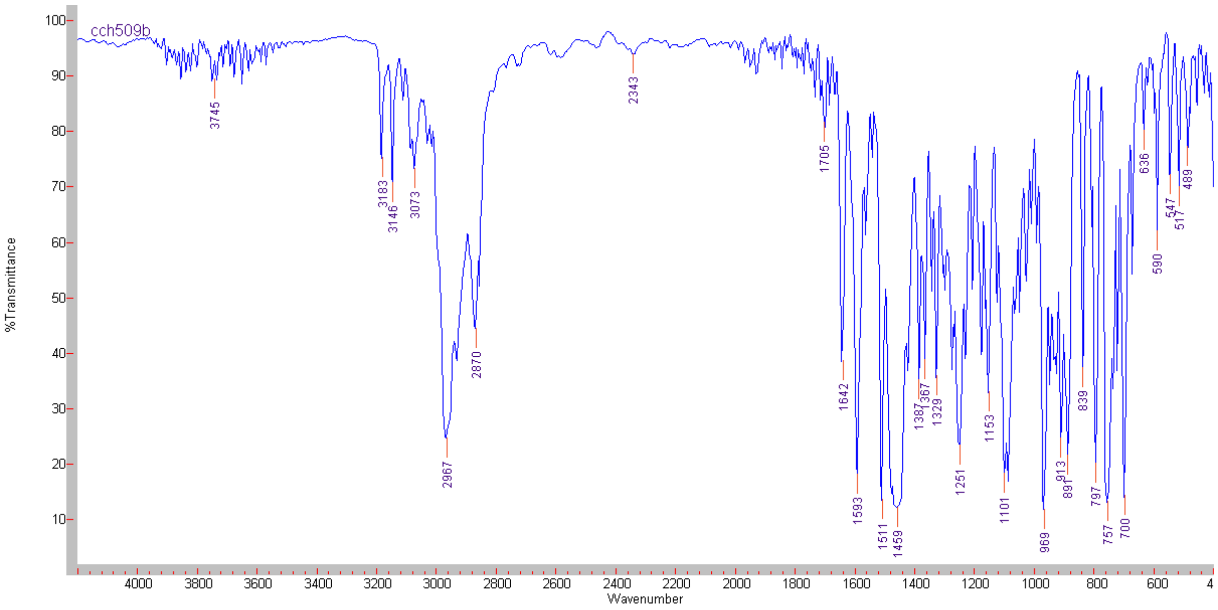

Figure S15. FT-IR spectrum of compound **4a** (KBr)

X-ray crystal structure analysis of compound 4a (ERK10081): A colorless plate-like specimen of $C_{39}H_{39}BF_5N_3O$, approximate dimensions 0.040 mm x 0.080 mm x 0.220 mm, was used for the X-ray crystallographic analysis. The X-ray intensity data were measured on a Kappa CCD APEXII Bruker APEXII Diffractometer system equipped with a fine-focus sealed tube Cu sealed tube (CuK_α, λ = 1.54178 Å) and a graphite monochromator. A total of 1429 frames were collected. The total exposure time was 20.55 hours. The frames were integrated with the Bruker SAINT software package using a wide-frame algorithm. The integration of the data using a monoclinic unit cell yielded a total of 44910 reflections to a maximum θ angle of 66.70° (0.84 Å resolution), of which 5909 were independent (average

redundancy 7.600, completeness = 99.8%, R_{int} = 8.60%, R_{sig} = 4.63%) and 4460 (75.48%) were greater than 2σ(F^2). The final cell constants of a = 9.5222(3) Å, b = 18.1892(5) Å, c = 19.3469(5) Å, β = 94.624(2)°, volume = 3340.00(16) $Å^3$, are based upon the refinement of the XYZ-centroids of 5140 reflections above 20 σ(I) with 9.172° < 2θ < 133.0°. Data were corrected for absorption effects using the Multi-Scan method (SADABS). The ratio of minimum to maximum apparent transmission was 0.872. The calculated minimum and maximum transmission coefficients (based on crystal size) are 0.8390 and 0.9680. The structure was solved and refined using the Bruker SHELXTL Software Package, using the space group $P2_1/c$, with Z = 4 for the formula unit, $C_{39}H_{39}BF_5N_3O$. The final anisotropic full-matrix least-squares refinement on F^2 with 449 variables converged at R1 = 4.16%, for the observed data and wR2 = 10.64% for all data. The goodness-of-fit was 1.023. The largest peak in the final difference electron density synthesis was 0.248 $e^-/Å^3$ and the largest hole was -0.258 $e^-/Å^3$ with an RMS deviation of 0.050 $e^-/Å^3$. On the basis of the final model, the calculated density was 1.335 g/cm^3 and F(000), 1408 e^-. CCDC Nr.: 2157862.

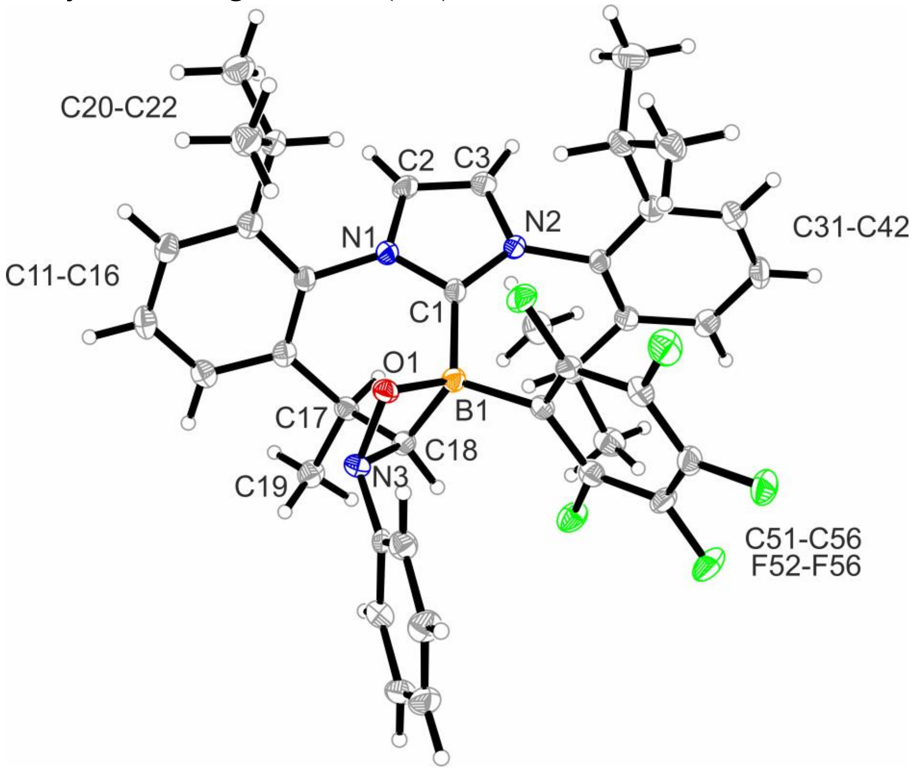

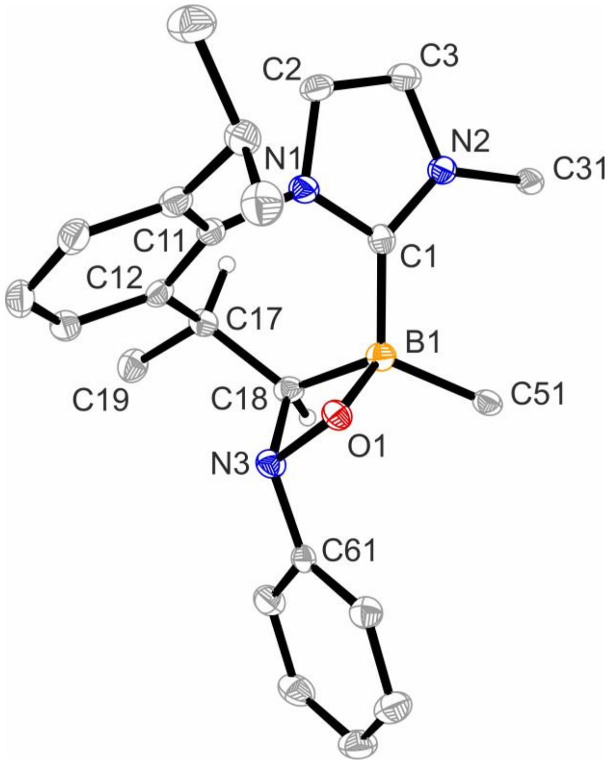

Figure S16. Crystal structure of compound **4a** (thermal ellipsoids: 50% probability)

Characterization of compound 5a (2nd Experiment):

Elemental analysis (%) [from crystals] calc. for $C_{39}H_{38}BF_5N_3O$: C, 69.86; H, 5.71; N, 6.27. Found: C, 69.64; H, 5.82; N, 6.37.

Decomposition point [from crystals]: 197 °C.

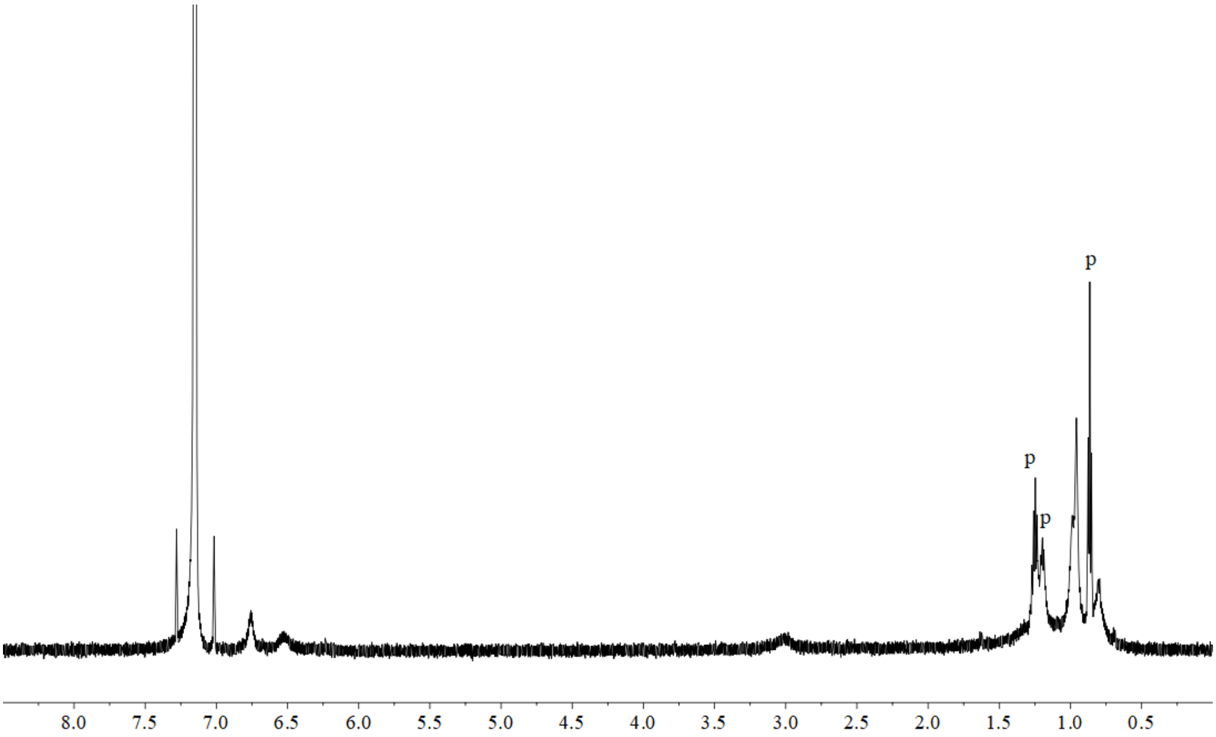

Figure S17. ^{1}H NMR (600 MHz, 299 K, C_6D_6) spectrum of compound **5a** [from crystals, pentane(p)]

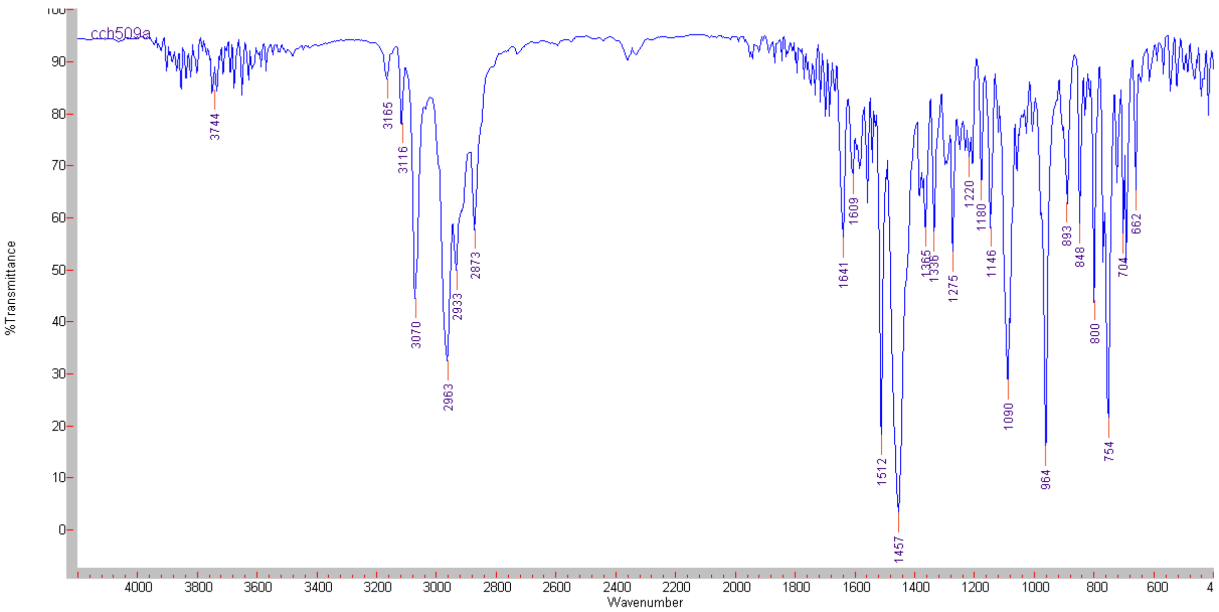

Figure S18. FT-IR spectrum of compound **5a** (KBr)

X-ray crystal structure analysis of compound 5a (erk9943): A orange prism-like specimen of $C_{39}H_{38}BF_5N_3O$, approximate dimensions 0.192 mm x 0.243 mm x 0.300 mm, was used for the X-ray crystallographic analysis. The X-ray intensity data were measured on a Bruker D8 Venture PHOTON III Diffractometer system equipped with a micro focus tube Mo Ims (MoK_{α}, λ = 0.71073 Å) and a MX mirror monochromator. A total of 673 frames were collected. The total exposure time was 3.74 hours. The frames were integrated with the Bruker SAINT software package using a narrow-frame algorithm. The integration of the data using a tetragonal unit cell yielded a total of 162403 reflections to a maximum θ angle of 26.75° (0.79 Å resolution), of which 7357 were independent (average redundancy 22.075, completeness = 99.8%, R_{int} = 9.31%, R_{sig} = 2.50%) and 6063 (82.41%) were greater than $2\sigma(F^2)$. The final cell constants of a = 23.5399(8) Å, b = 23.5399(8) Å, c = 24.9570(11) Å, volume = 13829.3(11) $Å^3$, are based upon the refinement of the XYZ-centroids of 9952 reflections above 20 σ(I) with 4.894° < 2θ < 53.39°. Data were corrected for absorption effects using the Multi-Scan method (SADABS). The ratio of minimum to maximum apparent transmission was 0.911. The calculated minimum and maximum transmission coefficients (based on crystal size) are 0.9720 and 0.9820. The structure was solved and refined using the Bruker SHELXTL Software Package, using the space group $I4_1/a$, with Z = 16 for the formula unit, $C_{39}H_{38}BF_5N_3O$. The final anisotropic full-matrix least-squares refinement on F^2 with 449 variables converged at R1 = 4.22%, for the observed data and wR2 = 9.95% for all data. The goodness-of-fit was 1.030. The largest peak in the final difference electron density synthesis was 0.269 $e^-/Å^3$ and the largest hole was -0.197 $e^-/Å^3$ with an RMS deviation of

0.039 e$^-$/Å^3. On the basis of the final model, the calculated density was 1.288 g/cm^3 and F(000), 5616 e$^-$. CCDC Nr.: 2157863.

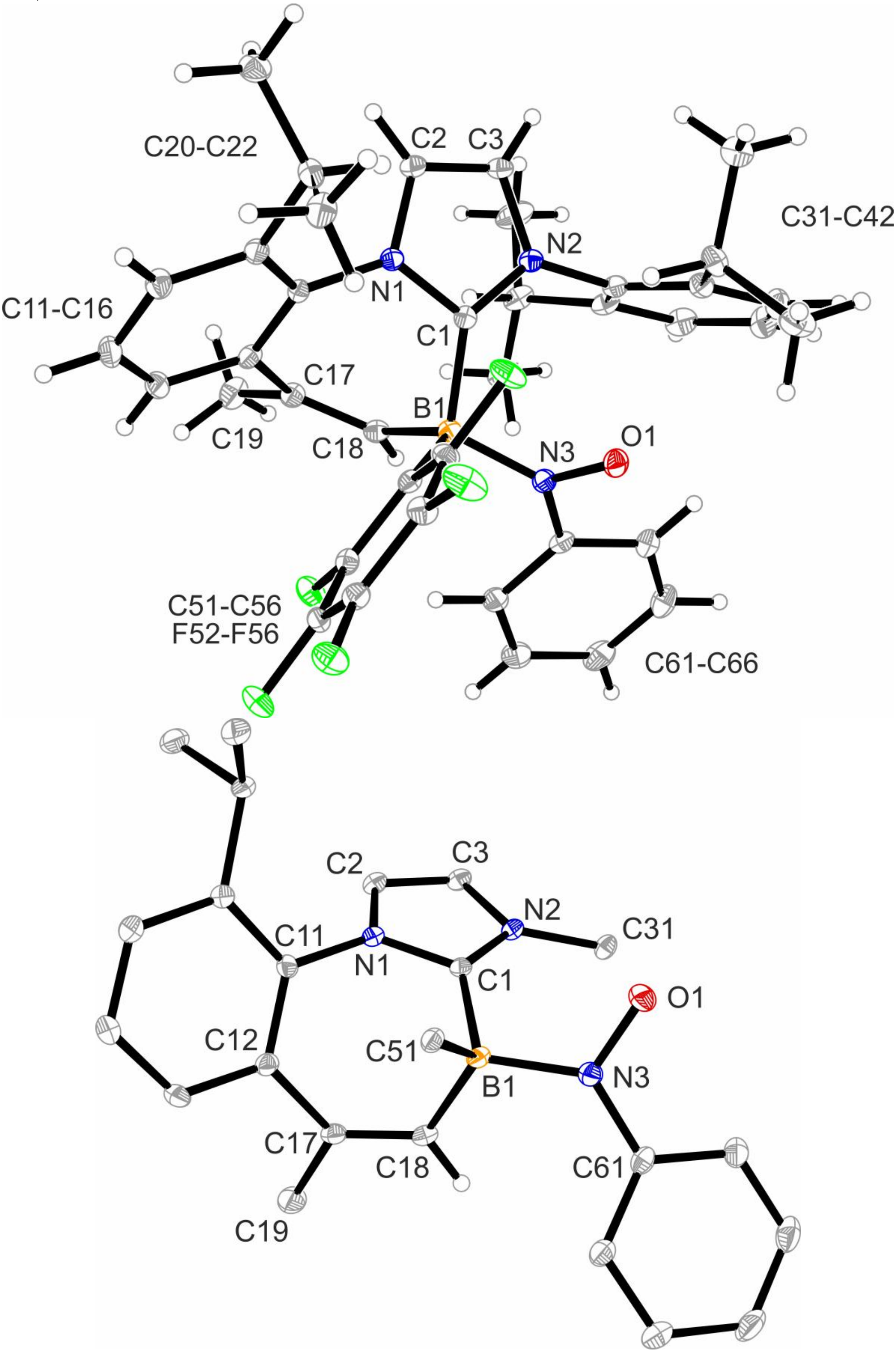

Figure S19. Crystal structure of compound **5a** (thermal ellipsoids: 30% probability)

Characterization of compound 6a (2nd Experiment):

Elemental analysis (%) [from crystals] calc. for $C_{39}H_{38}BF_5N_3O$: C, 69.86; H, 5.71; N, 6.27. Found: C, 69.21; H, 5.73; N, 6.15.

Decomposition point [from crystals]: 256 °C.

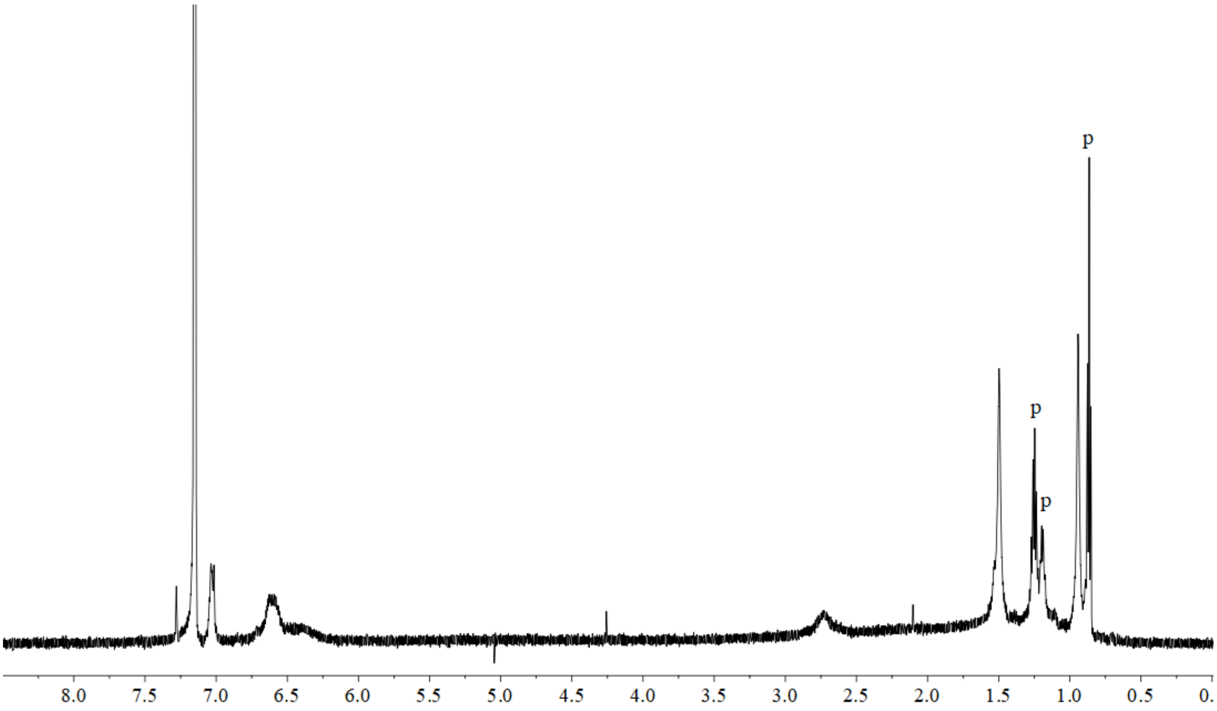

Figure S20. ^{1}H NMR (600 MHz, 299 K, C_6D_6) spectrum of compound **6a** [from crystals, pentane(p)]

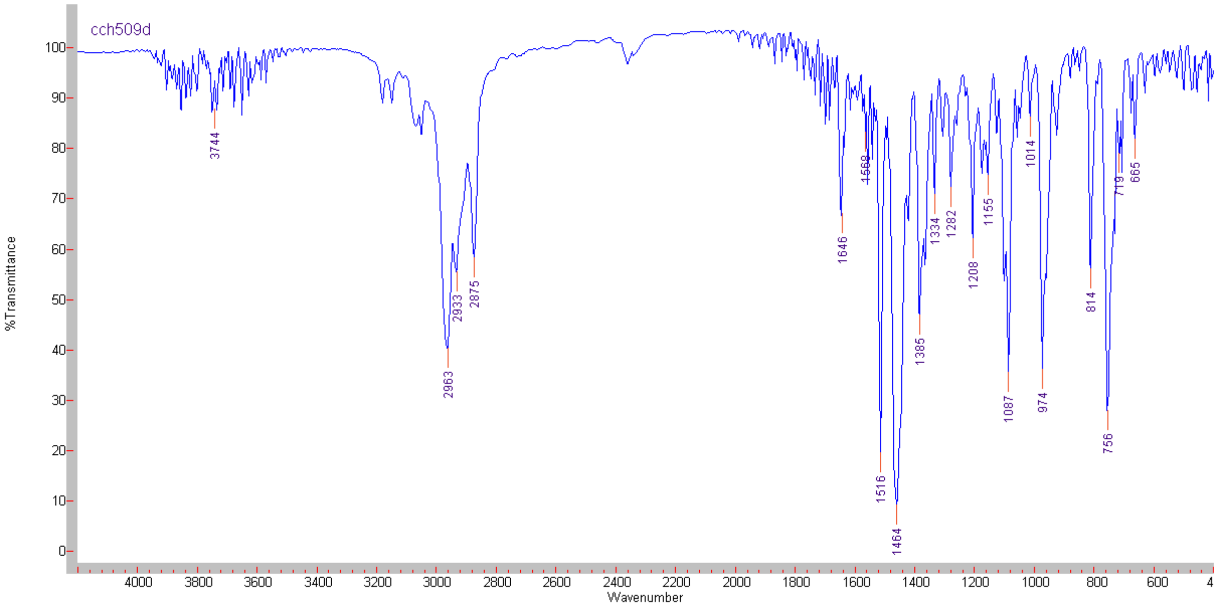

Figure S21. FT-IR spectrum of compound **6a** (KBr)

X-ray crystal structure analysis of compound 6a (erk10125): A yellow-orange plate-like specimen of $C_{39}H_{38}BF_5N_3O$, approximate dimensions 0.073 mm x 0.102 mm x 0.240 mm,

was used for the X-ray crystallographic analysis. The X-ray intensity data were measured (λ = 1.54178 Å). A total of 1922 frames were collected. The total exposure time was 23.15 hours. The frames were integrated with the Bruker SAINT software package using a wide-frame algorithm. The integration of the data using a monoclinic unit cell yielded a total of 76484 reflections to a maximum θ angle of 66.58° (0.84 Å resolution), of which 6046 were independent (average redundancy 12.650, completeness = 99.7%, R_{int} = 8.77%, R_{sig} = 3.00%) and 4654 (76.98%) were greater than $2\sigma(F^2)$. The final cell constants of a = 14.1129(2) Å, b = 16.9245(3) Å, c = 15.4437(3) Å, β = 111.5270(10)°, volume = 3431.47(10) $Å^3$, are based upon the refinement of the XYZ-centroids of 9937 reflections above 20 σ(I) with 8.072° < 2θ < 132.6°. Data were corrected for absorption effects using the Multi-Scan method (SADABS). The ratio of minimum to maximum apparent transmission was 0.884. The calculated minimum and maximum transmission coefficients (based on crystal size) are 0.8310 and 0.9440. The structure was solved and refined using the Bruker SHELXTL Software Package, using the space group $P2_1/n$, with Z = 4 for the formula unit, $C_{39}H_{38}BF_5N_3O$. The final anisotropic full-matrix least-squares refinement on F^2 with 547 variables converged at R1 = 5.68%, for the observed data and wR2 = 15.34% for all data. The goodness-of-fit was 1.046. The largest peak in the final difference electron density synthesis was 0.962 $e^-/Å^3$ and the largest hole was -0.296 $e^-/Å^3$ with an RMS deviation of 0.051 $e^-/Å^3$. On the basis of the final model, the calculated density was 1.298 g/cm^3 and F(000), 1404 e^-. CCDC Nr.: 2157864.

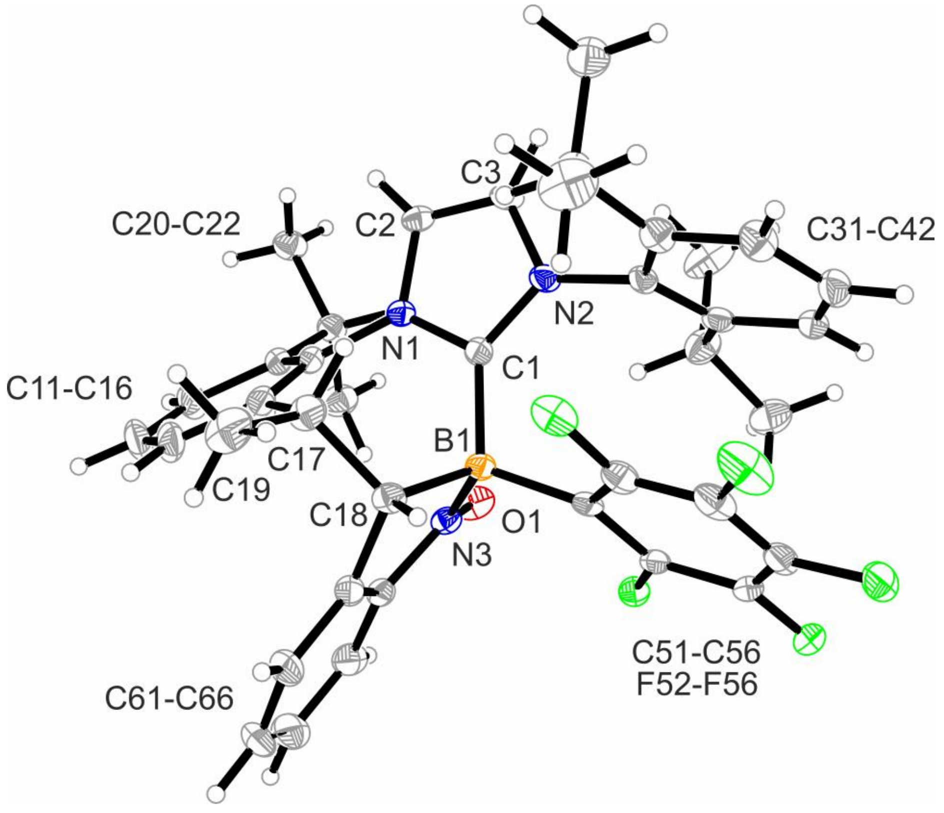

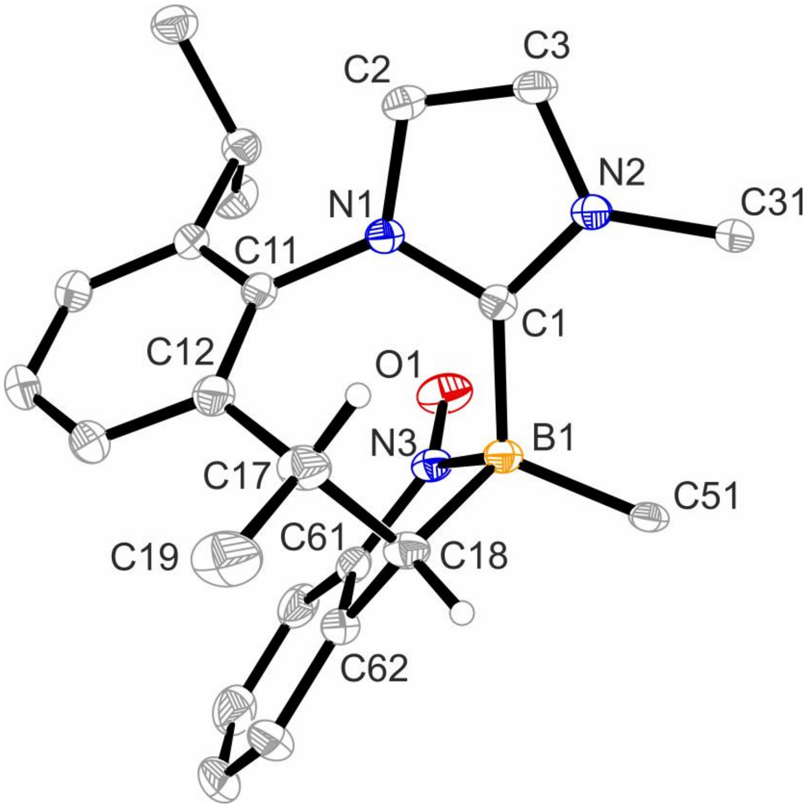

Figure S22. Crystal structure of compound **6a** (thermal ellipsoids: 30% probability)

Characterization of compound 7a (1st Experiment):

[*Comment*: ^{1}H and $^{13}C\{^{1}H\}$ NMR data of compound **7a** are consistent with those data reported in the literature.[6]]

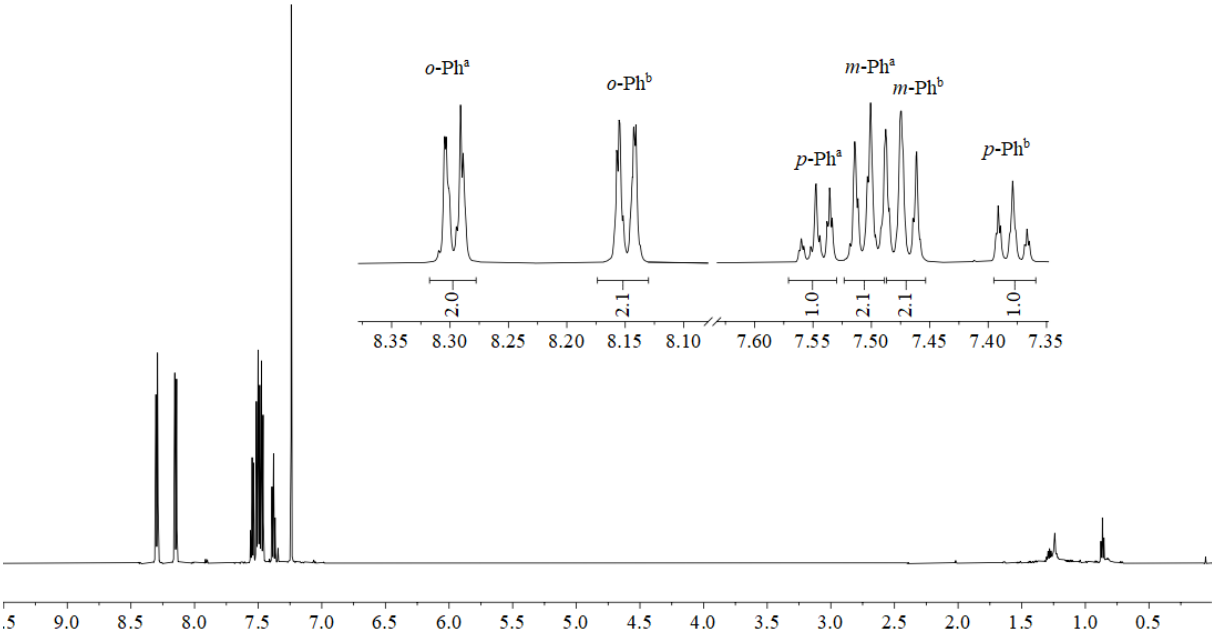

Figure S23. ^{1}H NMR (600 MHz, 299 K, $CDCl_3$) spectrum of compound **7a**

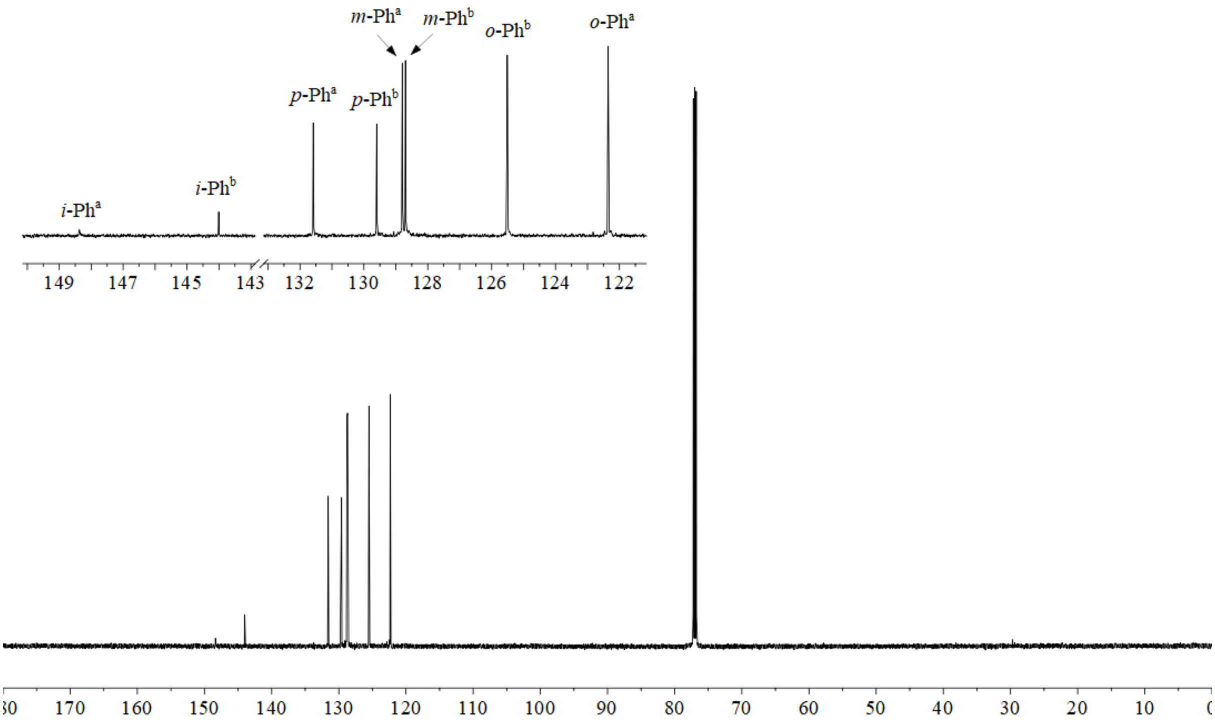

Figure S24. $^{13}C\{^1H\}$ NMR (600 MHz, 299 K, $CDCl_3$) spectrum of compound **7a**

3. Condition screening for the competitive formation of compounds 4a, 5a and 6a

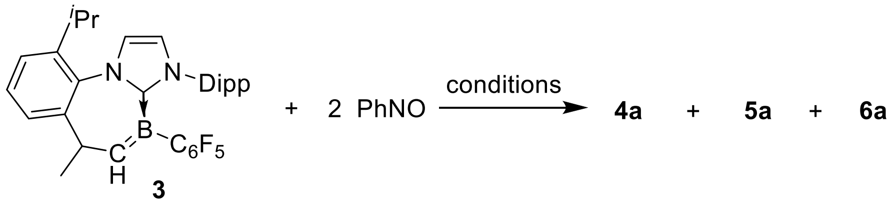

Entry	Reaction conditions		[2+2]	nitroso ene reaction		
	Solvent	temp./time	4a	5a	6a	5a+6a
1	C_6D_6	r.t./1h	28%	**35%**	**18%**	**53%**
2	Toluene	r.t./1h	26%	**36%**	**17%**	**53%**
3	Toluene	0 °C ~ r.t./14h	17%	23%	17%	40%
4	1,2-$F_2C_6H_4$	0 °C ~ r.t./14h	31%	19%	13%	32%
5	THF	r.t./1h	45%	26%	10%	36%
6	CH_3CN	r.t./14h	48%	24%	10%	34%
7	DMSO	r.t./1h	31%	24%	6%	30%

General procedure:

Compound **3** (112.6 mg, 0.2 mmol, 1.0 equiv.) and PhNO (42.8 mg, 0.4 mmol, 2.0 equiv.) were mixed and the respective solvent (1.5 mL) was added. The resulting mixture was stirred at the selected temperature for the given time. Then the reaction mixture was directly purified by flash chromatograph (SiO_2, eluent: pentane:ethyl acetate from 10:1 to 5:1) and the corresponding yields of products **4a**, **5a**, and **6a** were obtained.

4. UV-vis spectra of compounds 5a and 6a

Absorption spectra were recorded on a *JASCO* V-730 two-beam UV/Vis spectrometer.

For compound **5a**: $\varepsilon_{573} \approx 116$ L·mol^{-1}·cm^{-1}.

For compound **6a**: $\varepsilon_{500} \approx 99$ L·mol^{-1}·cm^{-1}; $\varepsilon_{475} \approx 975$ L·mol^{-1}·cm^{-1}. [see for comparison[7]]

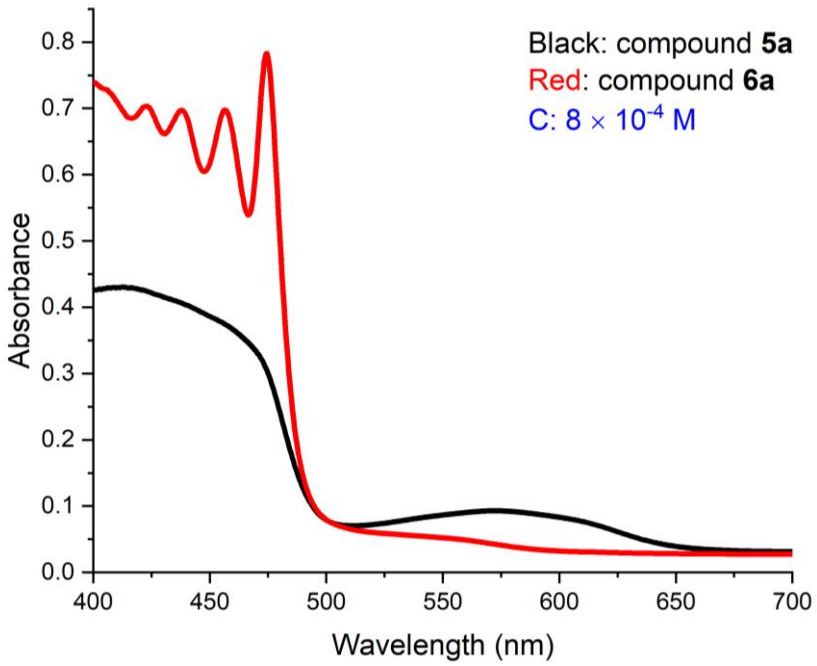

Figure S25a. UV-Vis spectra of compounds **5a** and **6a** in dichloromethane solution [c: 8×10^{-4} mol/L]

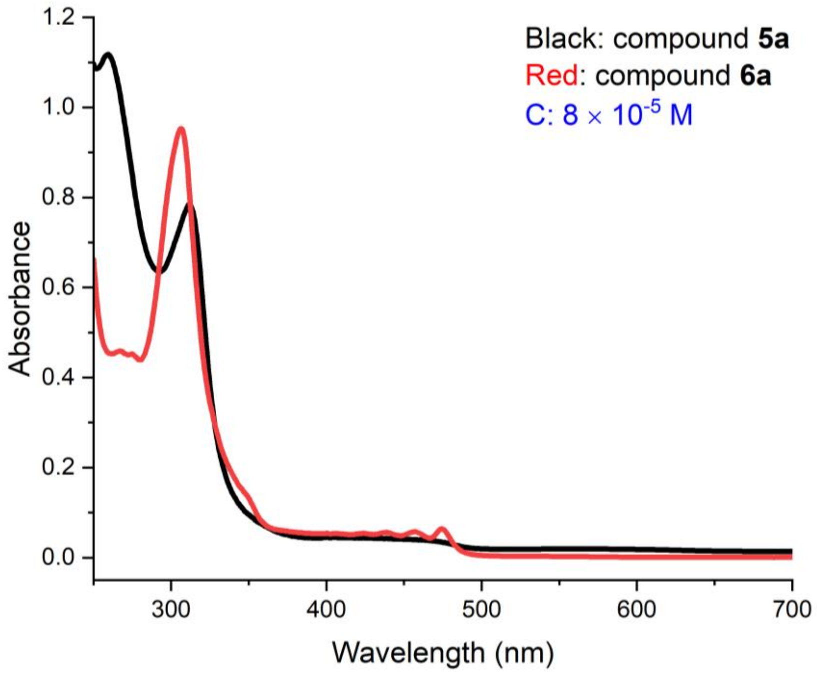

Figure S25b. UV-Vis spectra of compounds **5a** and **6a** in dichloromethane solution [c: 8×10^{-5} mol/L]

5. Cyclic voltammograms of compounds 5a and 6a

The Cyclic voltammograms were recorded on a CHI600E Electrochemical Workstation. Samples (compounds **5a**, **6a,** and TEMPO) were prepared in CH_3CN solution (*c*: 1 × 10^{-3} mol/L) with the Bu_4NPF_6 as additive (*c*: 1 ×10^{-1} mol/L).

Compound **5a**: $E_{1/2}$ = 0.53 V;

Compound **6a**: $E_{1/2}$ = 0.28 V;

TEMPO: $E_{1/2}$ = 0.66 V. [cf. $E_{1/2}$(TEMPO) = 0.68 V vs Ag/AgCl in CH_3CN][8]

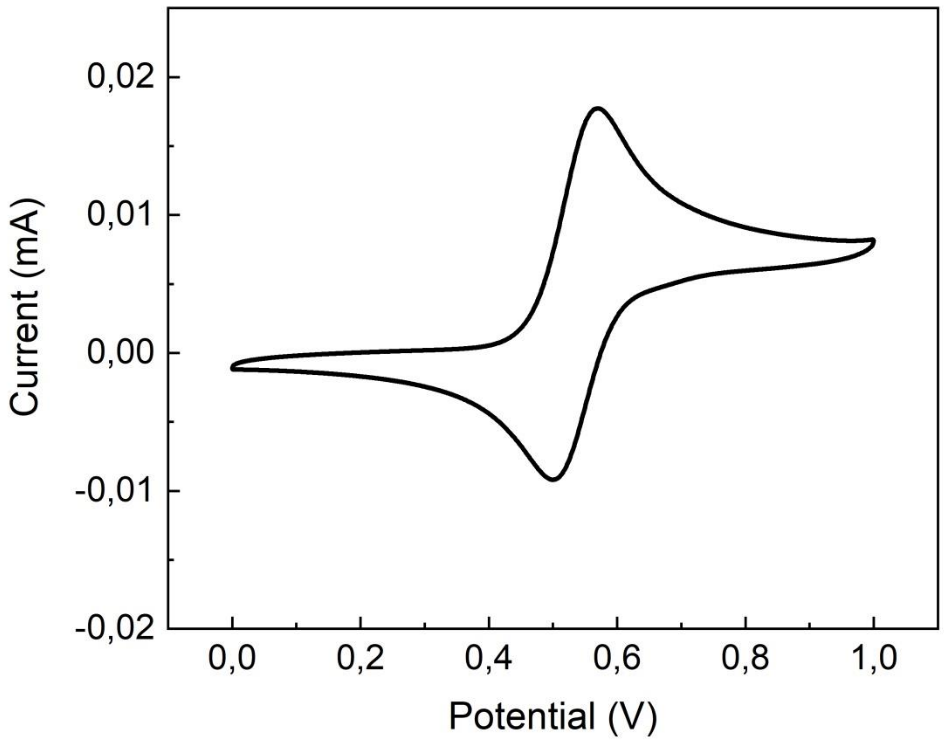

Figure S26. Cyclic voltammogram of compound **5a**

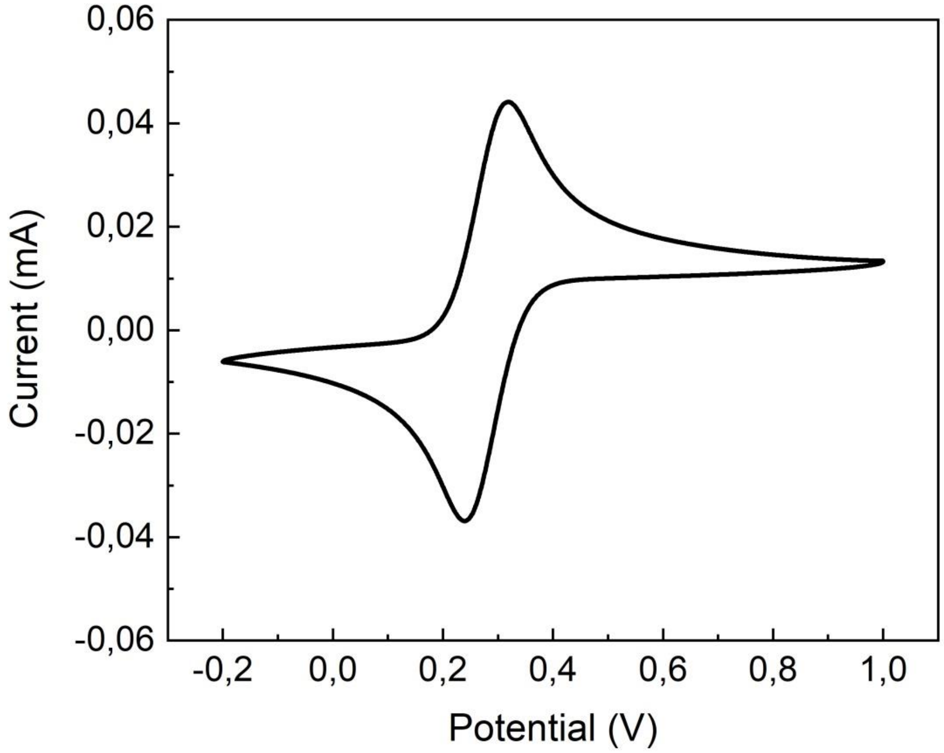

Figure S27. Cyclic voltammogram of compound **6a**

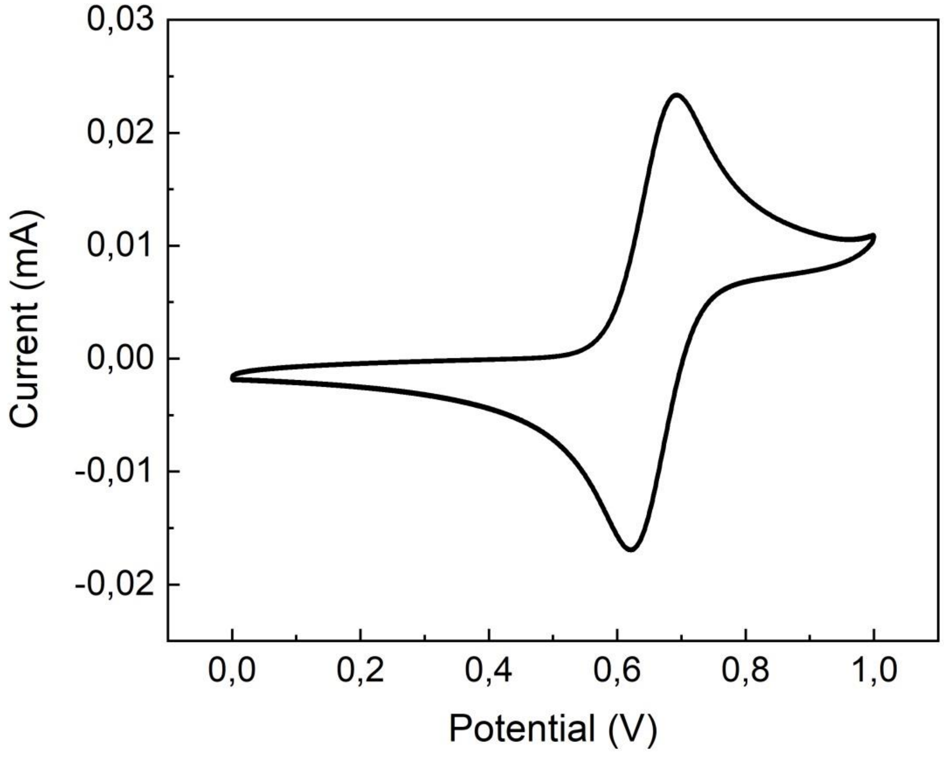

Figure S28. Cyclic voltammogram of TEMPO

6. EPR spectra of compounds 5a and 6a

Experimental part: All liquid-state EPR experiments were carried out on a BRUKER EMXnano spectrometer operating at X-band (9,6 GHz) in the range from 0–6000 G. The X-band continuous wave (CW) experiments were performed at room temperature. The applied microwave power was 1.259 mW. For the acquisition a sweep rate of 10 G s^{-1} and a time constant of 1.28 ms were used. 10000 data points were recorded using 10 scans. The modulation frequency was set to 100 kHz and a modulation amplitude of 0.5 G was used. For the measurements, 1 mM solutions of the sample in degassed dichloromethane were prepared. The resulting data were processed and simulated using the fitting program SpinFit by BRUKER and the simulation software Easyspin.[9,10]

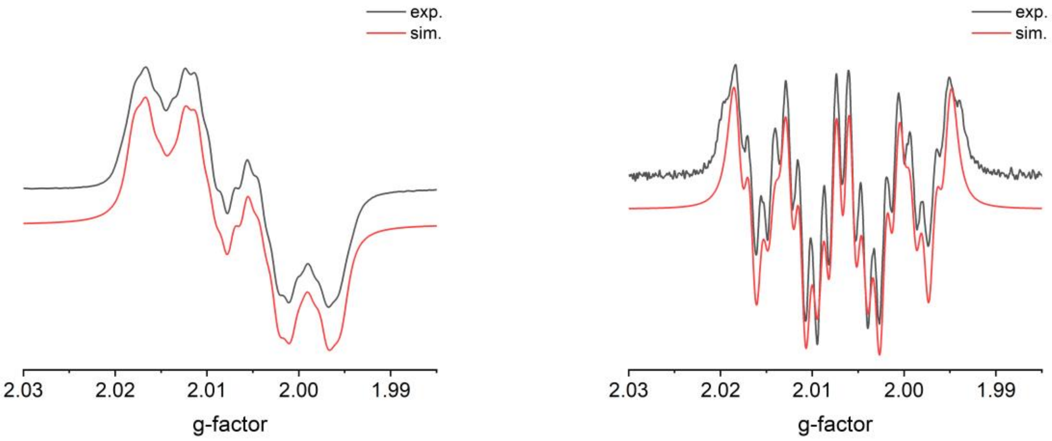

Figure S29. Liquid-state CW EPR spectrum of the radical compound **5a** recorded in dichloromethane at X-band and at room temperature. [*The black curve represents the experimental EPR spectrum, and the red curve is the best fit simulation. The left hand EPR spectrum shows the first derivative of the absorptive lineshape, while the right hand shows the second derivate of the absorptive lineshape.*]

EPR results: The liquid-state CW EPR spectrum in Figure S29 (left) shows a hyperfine splitting into several lines with a g-factor of 2.0066. The hyperfine splitting is caused by one ^{14}N nucleus (I = 1), one ^{11}B nucleus (I = 3/2), and the five ^{1}H nuclei (I = ½, 2:2:1) of the phenyl ring.

Table S1. g-factor and hyperfine coupling constants (in G and MHz) for **5a**, yielding the optimum agreement with the experimental CW EPR spectrum and corresponding second derivative EPR spectrum (see Figure S29).

Nuclei	*g*-factor	A(^{14}N)	A(^{11}B)	A(^{1}H)		
^{14}N, ^{11}B, ^{1}H	2.0066	9.30 G	4.46 G	2.25 G	0.92 G	0.51 G
		26.06 MHz	12.50 MHz	6.31 MHz	2.58 MHz	1.42 MHz

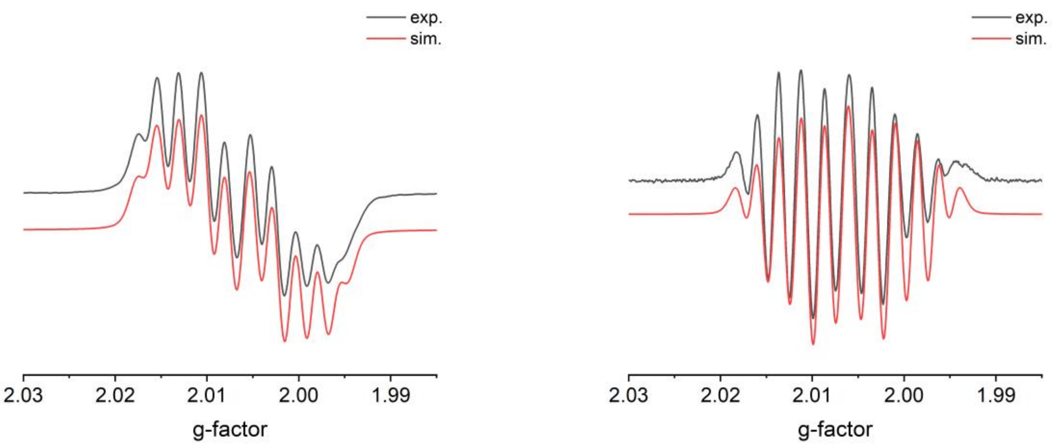

Figure S30. Liquid-state CW EPR spectrum of the radical compound **6a** recorded in dichloromethane at X-band and at room temperature. [*The black curve represents the experimental EPR spectrum, and the red curve is the best fit simulation. The left hand EPR spectrum shows the first derivative of the absorptive lineshape, while the right hand shows the second derivate of the absorptive lineshape.*]

EPR results: The liquid-state CW EPR spectrum in Figure S30 (left) shows a hyperfine splitting into several lines with a g-factor of 2.00604. The hyperfine splitting is caused by one ^{14}N nucleus (I = 1), one ^{11}B nucleus (I = 3/2) and the four ^{1}H nuclei (I = ½, 2:1:1) of the phenylene ring.

Table S2. g-factor and hyperfine coupling constants (in G and MHz) for **6a**, yielding the optimum agreement with the experimental CW EPR spectrum and the corresponding second derivative EPR spectrum (see Figure S30).

Nuclei	*g*-factor	A(^{14}N)	A(^{11}B)	A(^{1}H)		
^{14}N, ^{11}B, ^{1}H	2.00604	8.99 G	3.95 G	3.44 G	0.21 G	0.15 G
		25.18 MHz	11.07 MHz	9.65 MHz	0.58 MHz	0.43 MHz

The EPR spectra depicted in Figure S29-S30 (left) correspond to the first derivatives of the absorption EPR signal, because of the field modulation technique as applied here. It is known from literature that the resolution can be artificially enhanced by plotting the second derivative (Figure S29-S30 (right)).[11,12,13,14,15] Thus, using a simultaneous simulation of the first and second derivates, the hyperfine coupling constants for the radicals **5a** and **6a** could be determined (Table S1 and S2). The differences in the EPR spectra of the radicals **5a** and **6a** are caused by the different coordination and additional bonding at the phenyl/phenylene ring. Radical **5a** has five protons at the phenyl ring, where the protons in ortho and meta position are chemically equivalent (2:2:1). In comparison, radical **6a** includes a five-ring with boron, nitrogen, and the phenylene ring, and for this reason only four protons are left at the phenylene ring.

DFT calculations of spin densities: For the crystal structures obtained by X-ray diffraction, geometry optimization was restricted to the position of hydrogen atoms as these are not included in the XRD structure refinement. All compounds were optimized using ORCA 4.1.2., a def2-TZVP basis set including D3BJ dispersion correction, and the PBE0 functional.[16,17,18]

(a) (b)

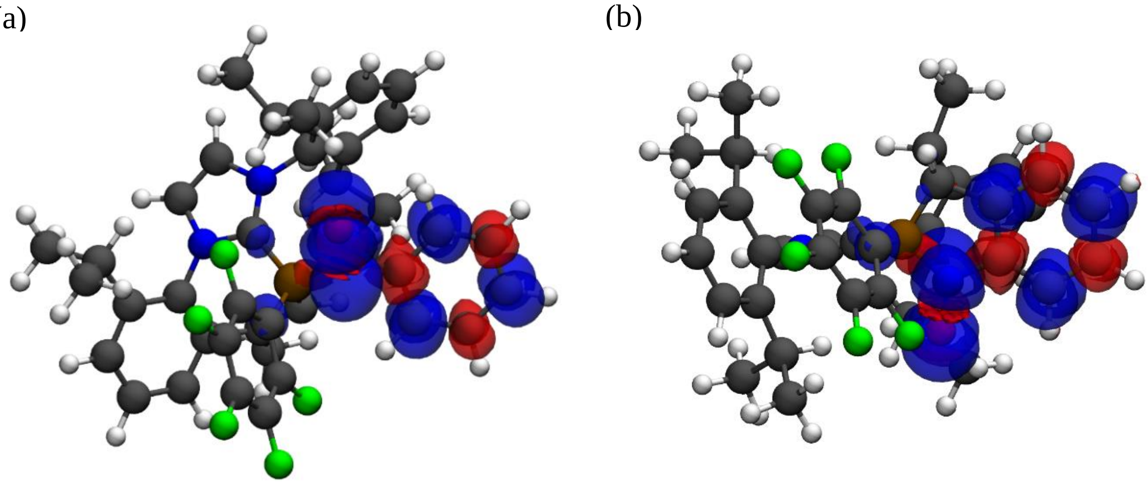

Figure S31. Spin densities of the radicals **5a** (a) and **6a** (b).

The calculated spin density via DFT in Figure S31a is significant at the oxygen where the radical is located and at the oxygen bonded nitrogen nucleus. Moreover, the spin density is higher at the nitrogen nucleus than at the boron nucleus, which correlates well with the differences in hyperfine coupling constants, cf. Table S1 and S2, that is, higher hyperfine coupling constants values are caused by a higher probability density of the electron at the nucleus. Furthermore, spin density is located at the conjugated phenyl ring, explaining the presence of hyperfine couplings to the abundant ^{1}H of the phenyl ring. In Figure S31b, the spin density located at the conjugated phenylene ring is a slightly higher, which correlates with the experimental determined hyperfine coupling constant for the two equivalent protons of 3.44 G (cf. Table S2).

7. Synthesis and characterization of compound 4b

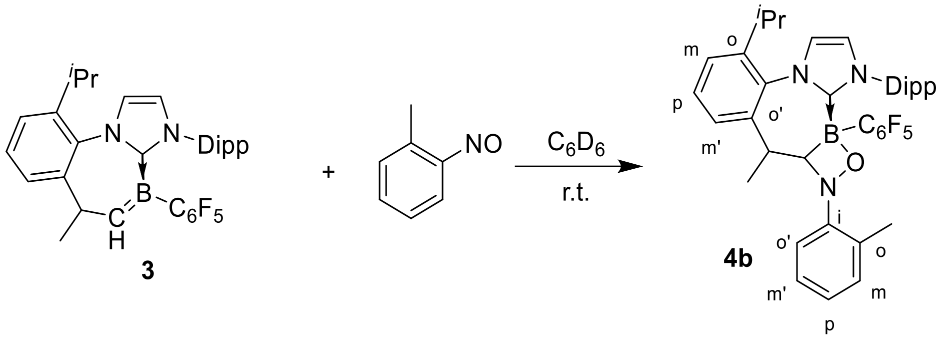

1st Experiment: Compound **3** (28.2 mg, 0.05 mmol, 1.0 equiv.) and 2-nitrosotoluene (12.1 mg, 0.1 mmol, 2.0 equiv.) were mixed and C_6D_6 (0.5 mL) was added. The resulting mixture was directly characterized by ^{1}H, ^{19}F, and ^{11}B NMR experiments (see Figure S32-S34; for comparison: Figure S36, S39, S40).

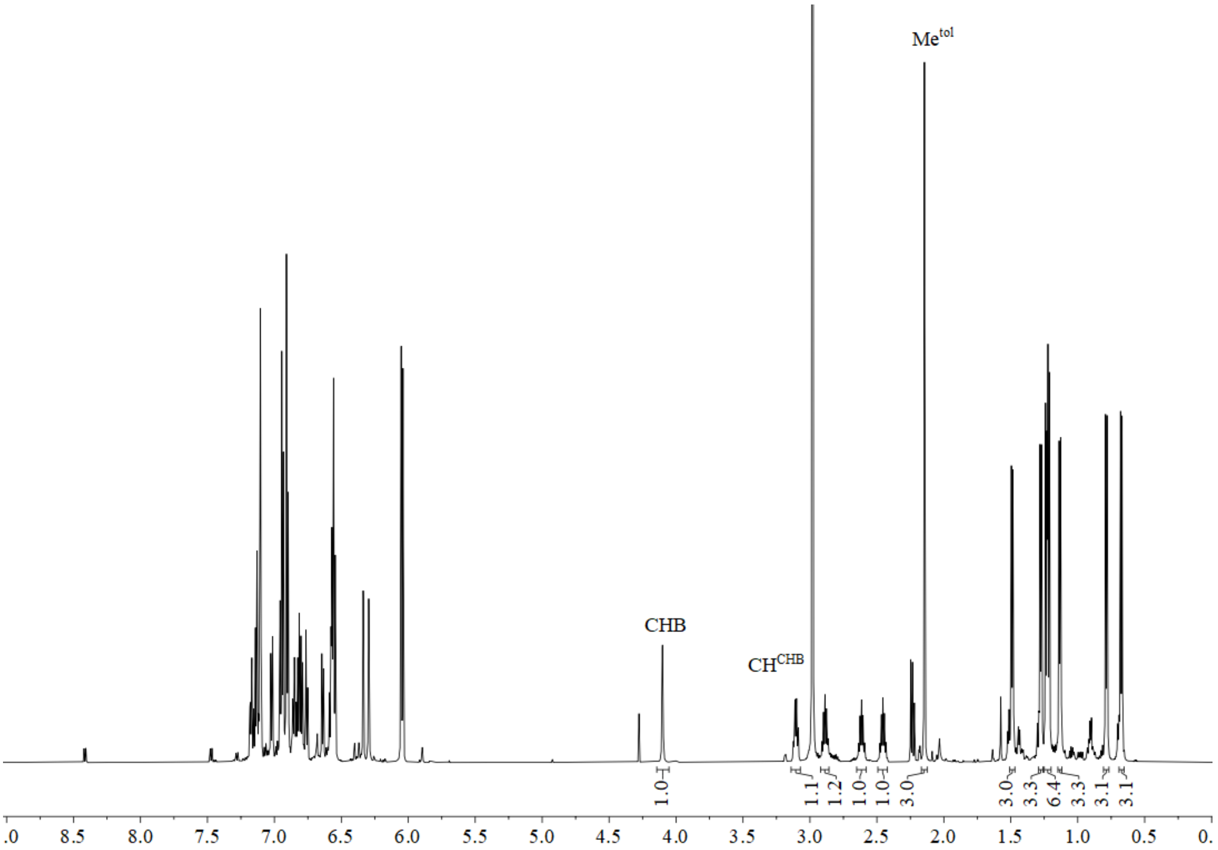

Figure S32. ^{1}H NMR (600 MHz, 299 K, C_6D_6) spectrum of the reaction mixture

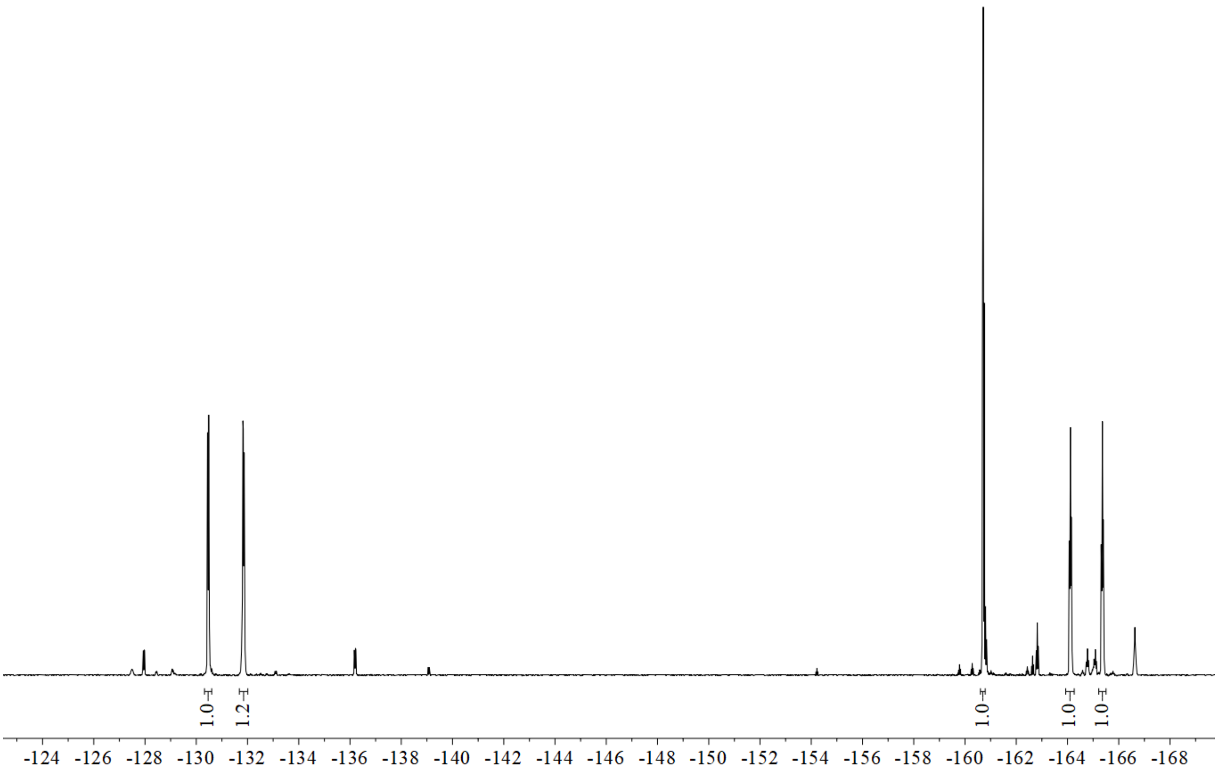

Figure S33. ^{19}F NMR (564 MHz, 299 K, C_6D_6) spectrum of the reaction mixture

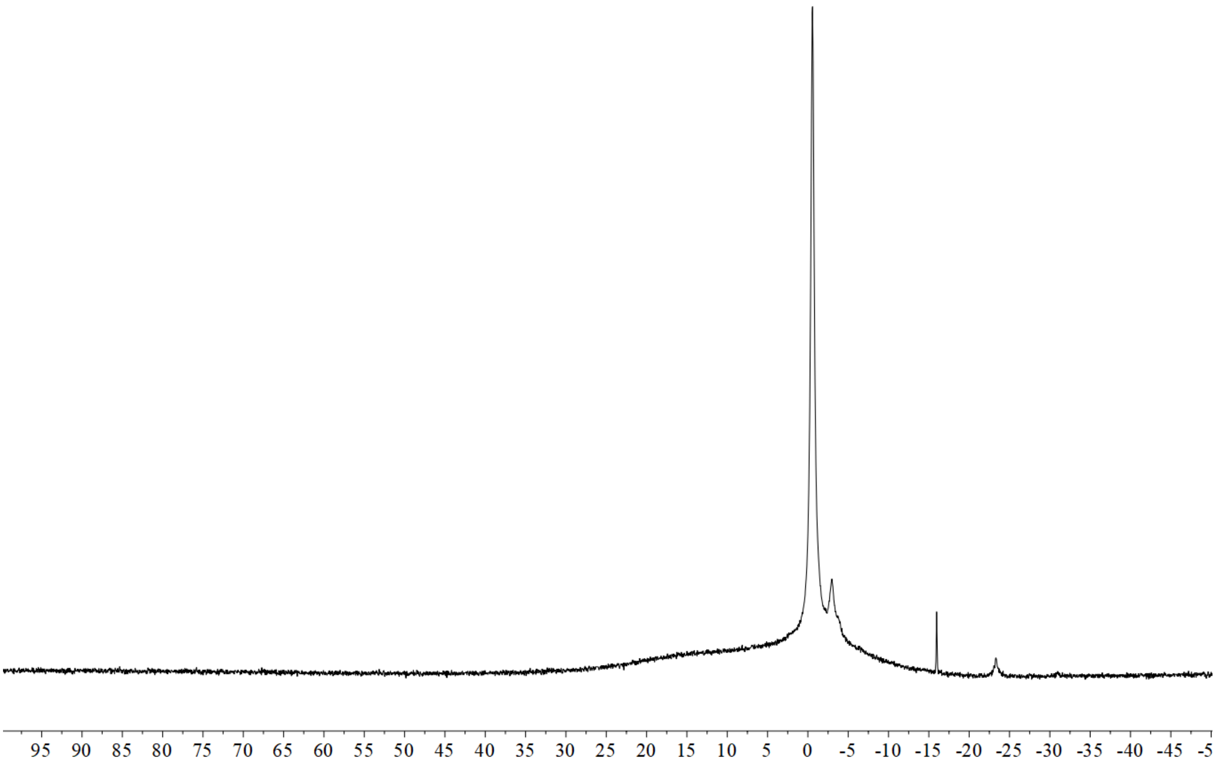

Figure S34. ^{11}B{^{1}H} NMR (192 MHz, 299 K, C_6D_6) spectrum of the reaction mixture

2nd Experiment: Compound **3** (5.6 mg, 0.01 mmol, 1.0 equiv.) and 2-nitrosotoluene (2.4 mg, 0.02 mmol, 2.0 equiv.) were mixed and toluene (1.0 mL) was added. The resulting mixture was stirred at r.t. for 10 min and directly used for the EPR experiment (see Figure S35).

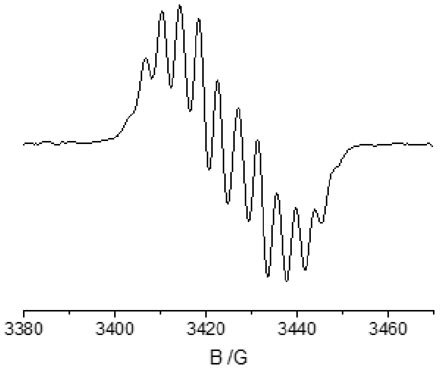

Figure S35. Liquid-state CW EPR spectrum of the reaction mixture recorded in toluene at X-band and at room temperature.

Table S3. g-factor and hyperfine coupling constants (in G) for the reaction mixture, yielding the optimum agreement with the experimental CW EPR spectrum.

Nuclei	*g*-factor	A(^{14}N)	A(^{11}B)	A(^{1}H)		
^{14}N, ^{11}B, ^{1}H	2.00634	8.83 G	3.84 G	3.49 G	0.20 G	0.10 G

3rd Experiment: Compound **3** (56.3 mg, 0.1 mmol, 1.0 equiv.) and 2-nitrosotoluene (12.1 mg, 0.1 mmol, 1.0 equiv.) were mixed and C_6D_6 (1.0 mL) was added. The resulting mixture was stirred at r.t. for 1h. Then the solvent was removed in vacuo and the resulting solid was dissolved with CH_2Cl_2/heptane (v/v 1:5, ca. 3.0 mL). The mixture was kept at -35 °C for 3 days. The formed white crystalline material was collected, washed with cold pentane (2 × 0.2 mL) and dried in vacuo to give compound **4b** as a white solid (ca. 55 mg, 80%).

Elemental analysis (%) calc. for $C_{40}H_{41}BF_5N_3O{\cdot}0.5CH_2Cl_2$: C, 66.81; H, 5.81; N, 5.77. Found: C, 66.73; H, 5.90; N, 6.07.

NMR data of compound **4b** were obtained from a solution of the obtained white solid in CD_2Cl_2 at r.t. [Dipp: 2,6-diisopropylphenyl]

^{1}**H NMR** (600 MHz, 299 K, CD_2Cl_2) δ [7.46 (*m*-), 7.43 (*p*-), 7.30 (*m'*-)](each m, each 1H, C_6H_3), [7.34, 7.19] (each m, each 1H, =CH), {7.25 (t, $^{3}J_{HH}$ = 7.8 Hz, 1H, *p*-), [7.10, 7.02](each d, $^{3}J_{HH}$ = 7.8 Hz, each 1H, *m*-)}(Dipp), [6.78 (1H, *m*-)), 6.70 (*m'*-), 6.69 (o'-), 6.56 (*p*-)](each m, each 1H, CH^{tol}), 3.72 (s, 1H, HCB), {3.16 (sept, $^{3}J_{HH}$ = 6.8 Hz, 1H), [1.49, 1.24](each d, $^{3}J_{HH}$ = 6.8 Hz, each 3H)}(iPr^{C6H3}), 3.07 (q, $^{3}J_{HH}$ = 7.2 Hz, 1H, CH^{CHB}), [2.63 (sept, $^{3}J_{HH}$ = 6.7 Hz, 1H), 1.25 (d, $^{3}J_{HH}$ = 6.7 Hz, 3H), 1.01 (d, $^{3}J_{HH}$ = 6.7 Hz, 3H)](iPr^{Dipp}), [2.50 (sept, $^{3}J_{HH}$ = 6.7 Hz, 1H), 1.33 (d, $^{3}J_{HH}$ = 6.7 Hz, 1H), 1.06 (d, $^{3}J_{HH}$ = 6.7 Hz,

1H)](iPr^{Dipp}), 1.94 (s, 3H, Me^{tol}), 1.56 (d, $^{3}J_{HH}$ = 7.1 Hz, 3H, Me^{CHC6H3}).

^{13}C{^{1}H} NMR (151 MHz, 299 K, CD_2Cl_2) δ 164.8 (br, BC), [154.1 (*i*-C), 130.5 (*m*-CH), 127.5 (*o*-C), 125.4 (*m'*-CH), 120.7 (*p*-CH), 114.5 (*o'*-CH](tol), [145.75, 145.74](*o*-Dipp), [141.9 (*o*-), 140.6 (*o'*-)](C_6H_3), 135.3 (*i*-C_6H_3), 133.3 (*i*-Dipp), 130.8 (*p*-Dipp), 128.9 (*p*-C_6H_3), [125.5 (*m'*-), 124.5 (*m*-)](C_6H_3), [125.0, 124.6](=CH), [123.9, 123.6](*m*-Dipp), 74.7 (br, HCB), 39.0 (CH^{CHB}), 29.6 (*o*-CH^{iPr}(C_6H_3)), [29.11 (m, CH), 26.7 (Me), 21.2 (d, *J* = 4.1 Hz, Me)](iPr^{Dipp}), [29.09 (CH), 27.0 (Me), 21.5 (d, *J* = 5.0 Hz, Me)](iPr^{Dipp}), [24.6, 22.3](*o*-Me^{iPr}(C_6H_3)), 20.0 (Me^{CHC6H3}), 19.8 (Me^{tol}), [C_6F_5 not listed].

^{11}B{^{1}H} NMR (192 MHz, 299 K, CD_2Cl_2) δ -0.4 ($\nu_{1/2}$ ≈ 55 Hz).

^{19}F NMR (564 MHz, 299 K, CD_2Cl_2) δ [-130.9, -131.4](each m, each 1F, *o*-C_6F_5), -161.3 (t, $^{3}J_{FF}$ = 19.7 Hz, *p*-C_6F_5), [-164.8, -165.7](each m, each 1F, *m*-C_6F_5), [$\Delta\delta^{19}F_{m,p}$ = 3.5, 4.4].

^{1}H,^{13}C gHMBC (600 MHz/151 MHz, 299 K, CD_2Cl_2)[selected traces] δ ^{1}H/^{13}C {[7.34/164.8, 125.0], [7.19/164.8, 124.6]}(=CH/BC, =CH), [3.72/164.8, 154.1, 140.6, 121.6, 20.0](HCB/BC, *i*-tol, *o'*-C_6H_3, *i*-C_6F_5, Me^{CHC6H3}), [3.07/140.6, 135.3, 125.5, 20.0](CH^{CHB}/*o'*-C_6H_3, *i*-C_6H_3, *m'*-C_6H_3, Me^{CHC6H3}).

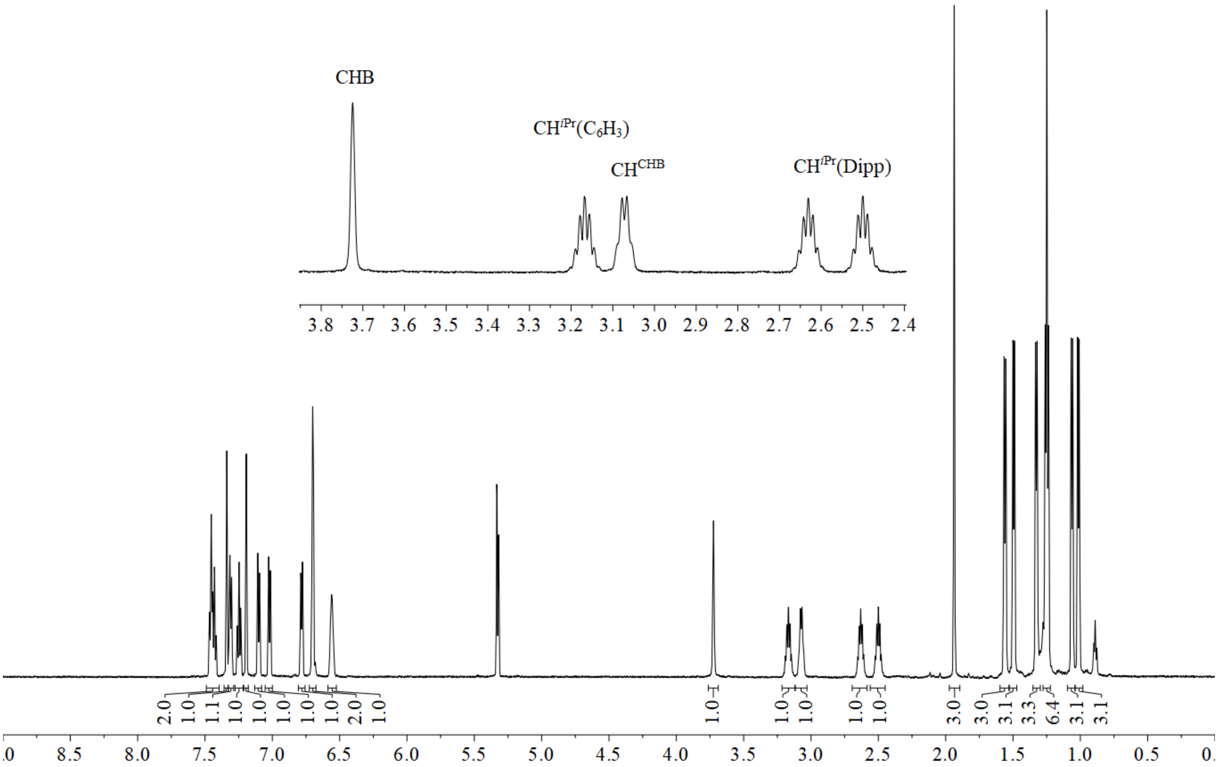

Figure S36a. ^{1}H NMR (600 MHz, 299 K, CD_2Cl_2) spectrum of compound **4b**

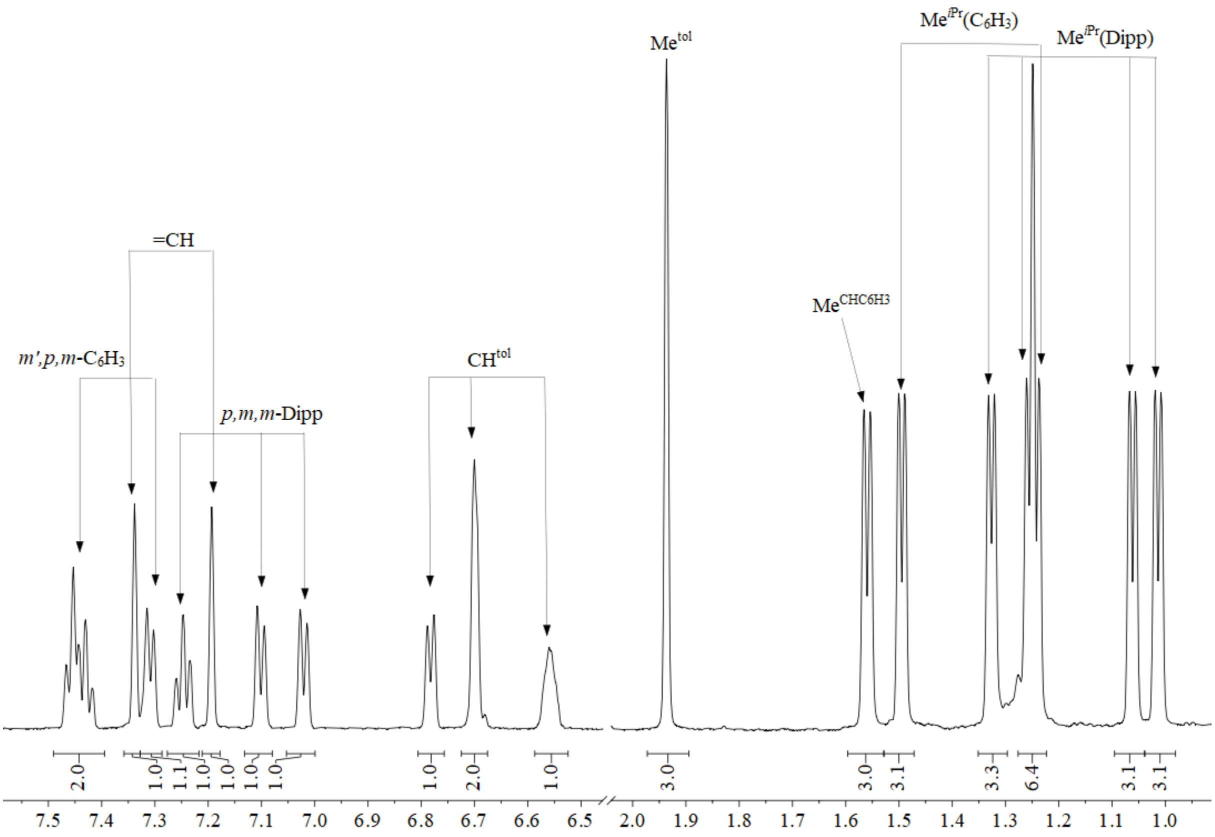

Figure S36b. ^{1}H NMR (600 MHz, 299 K, CD_2Cl_2) spectrum of compound **4b**

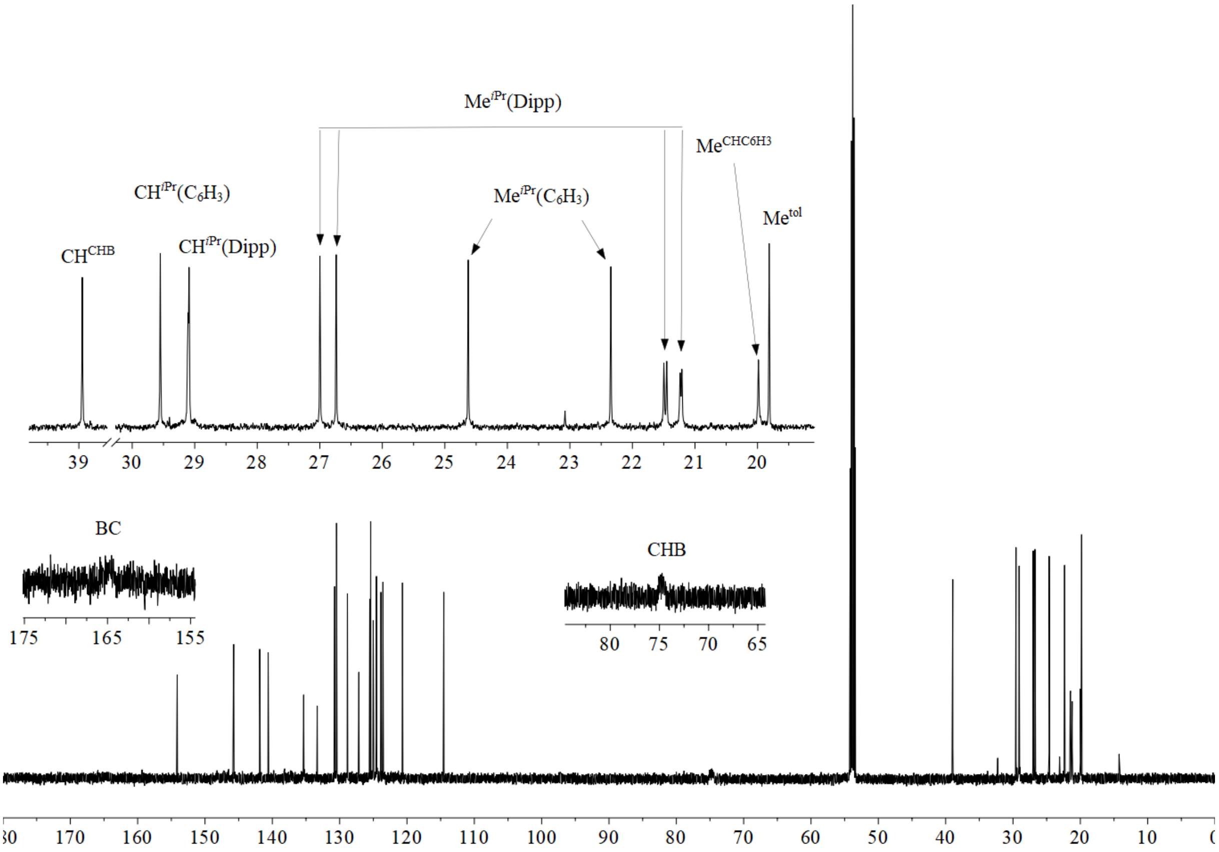

Figure S37a. ^{13}C{^{1}H} NMR (151 MHz, 299 K, CD_2Cl_2) spectrum of compound **4b**

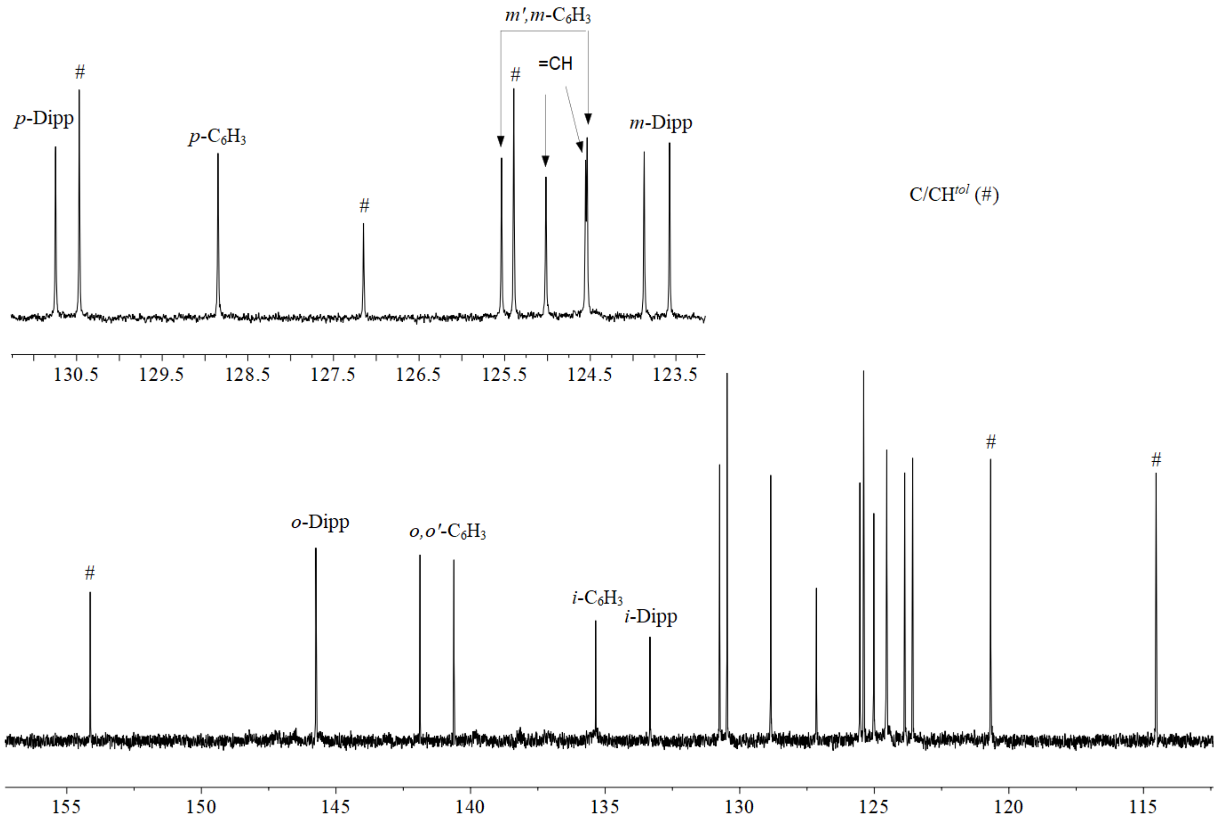

Figure S37b. $^{13}C\{^1H\}$ NMR (151 MHz, 299 K, CD_2Cl_2) spectrum of compound **4b**

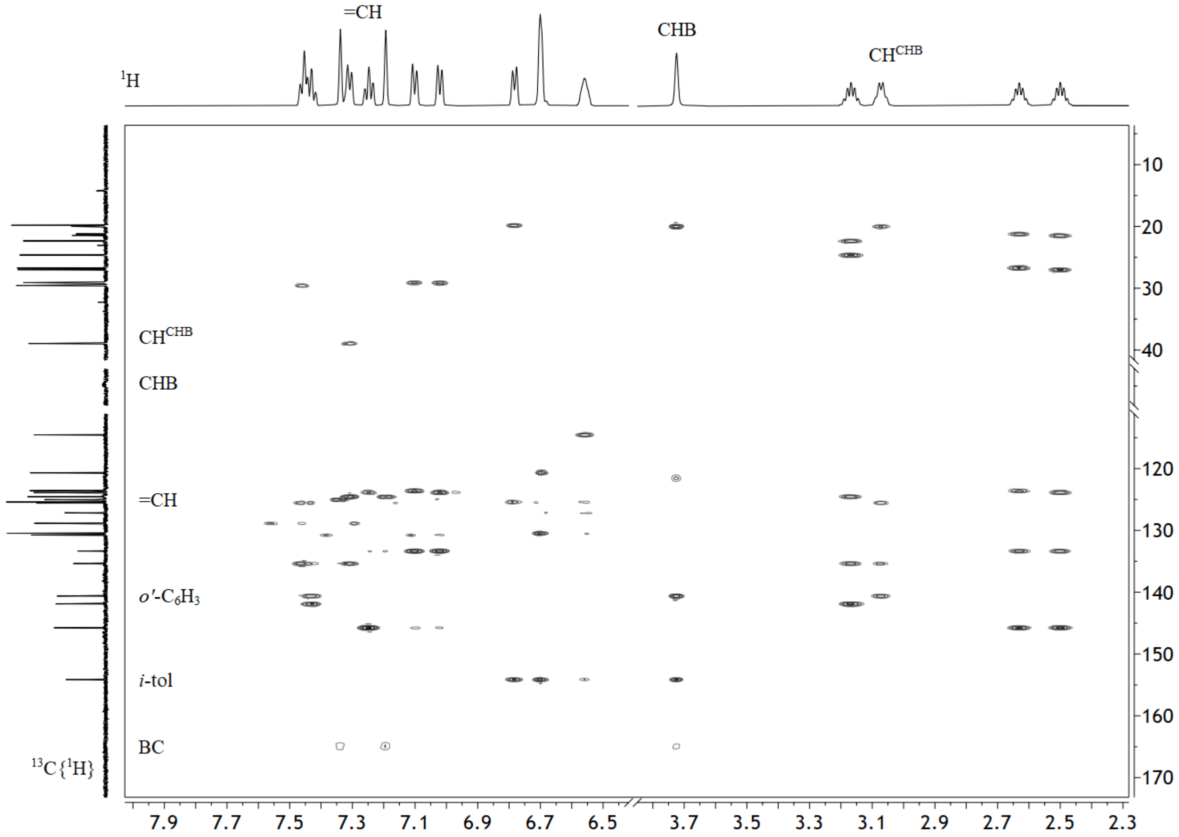

Figure S38. $^1H,^{13}C$ gHMBC (600/151 MHz, 299 K, CD_2Cl_2) spectrum of compound **4b**

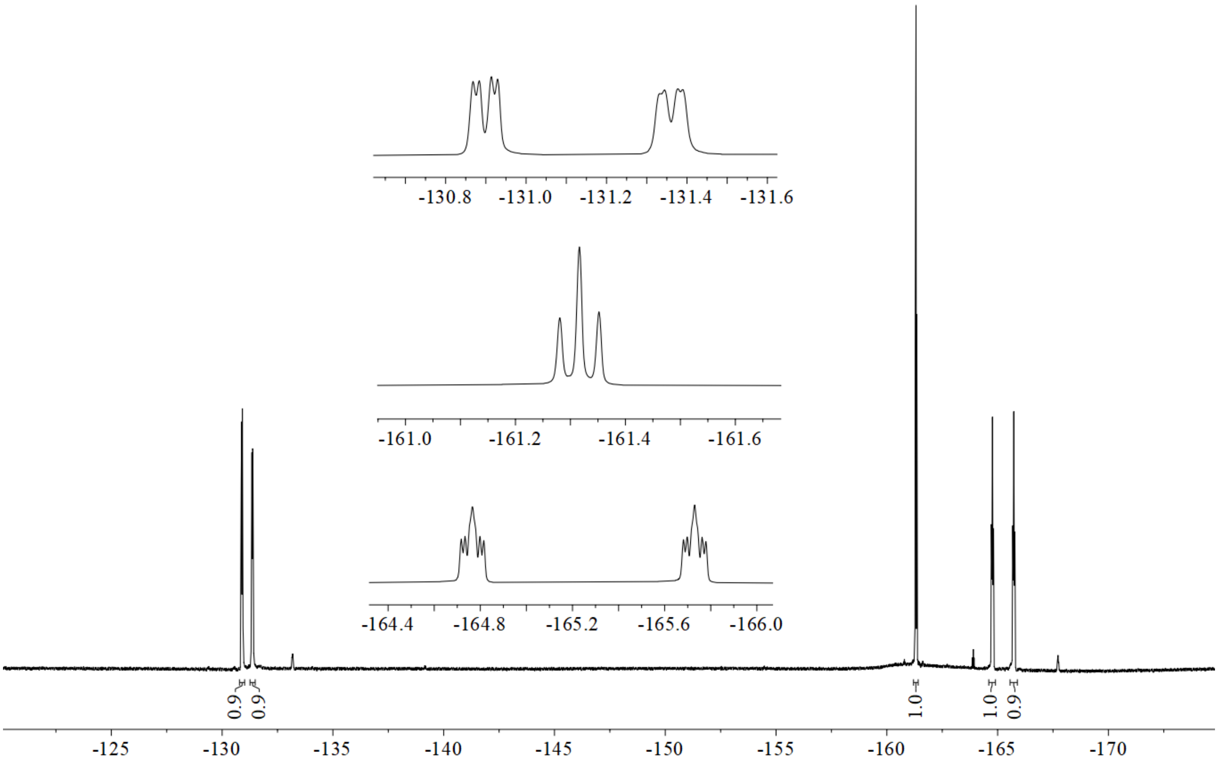

Figure S39. ^{19}F NMR (564 MHz, 299 K, CD_2Cl_2) spectrum of compound **4b**

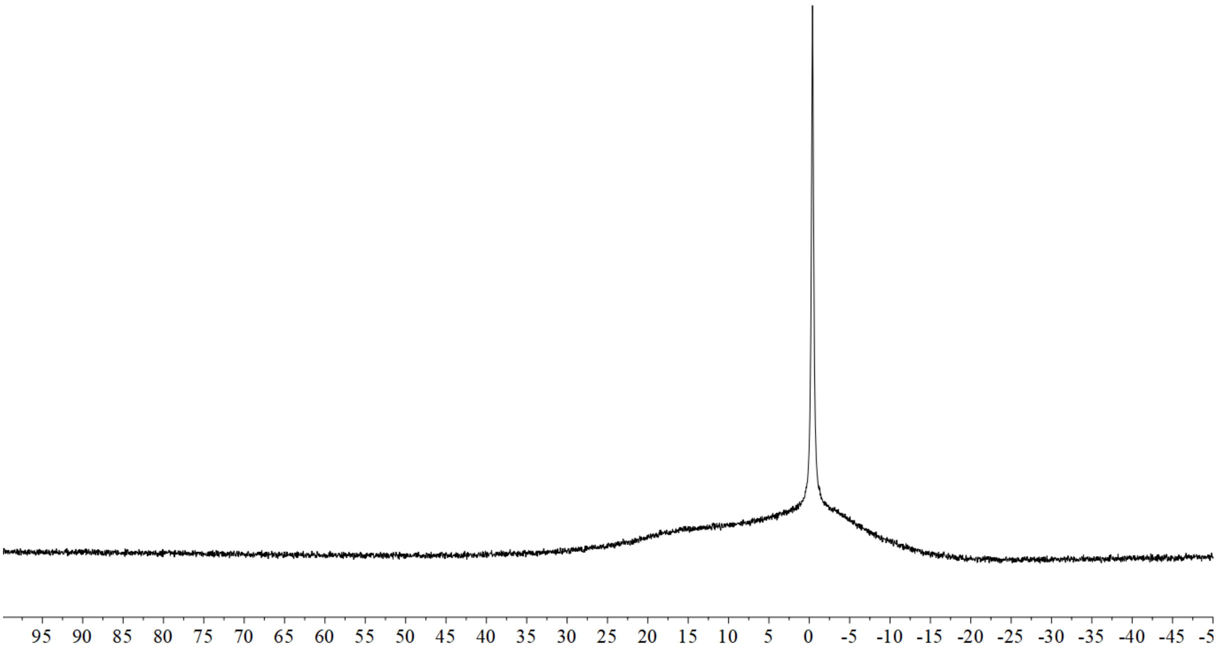

Figure S40. ^{11}B{^{1}H} NMR (192 MHz, 299 K, CD_2Cl_2) spectrum of compound **4b**

8. Synthesis and characterization of compound 4c

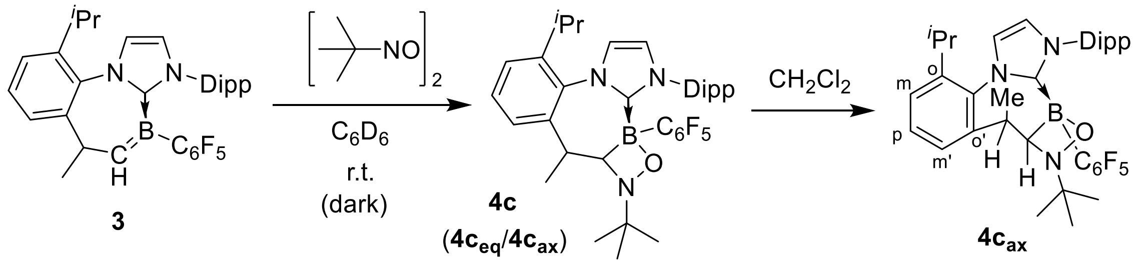

1st Experiment:

Step 1: Compound **3** (28.2 mg, 0.05 mmol, 1.0 equiv.) and [^{t}Bu-NO]$_2$ (8.7 mg, 0.05 mmol, 1.0 equiv. (2.0 equiv. "NO")) were mixed and C_6D_6 (0.5 mL) was added. The resulting mixture was stirred at room temperature for 14 h in the dark. The obtained mixture was directly characterized by ^{1}H, ^{19}F, and ^{11}B NMR experiments (see Figure S41-S43). [*Comment: the reaction was conducted in the dark to reduce the photolysis*[19] *of [^{t}Bu-NO]$_2$, since the generated NO would reacted with* **3** *via a formal [2+1+1] cycloaddition reaction*[5].]

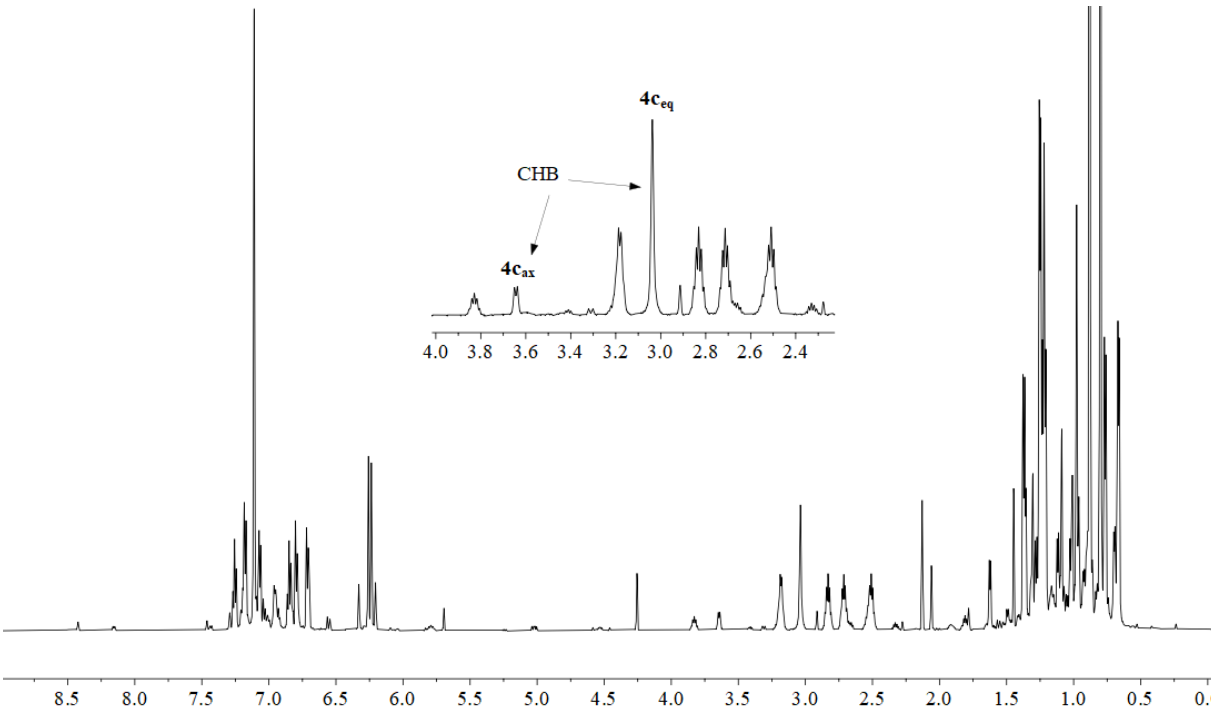

Figure S41. ^{1}H NMR (600 MHz, 299 K, C_6D_6) spectrum of the reaction mixture [step 1]

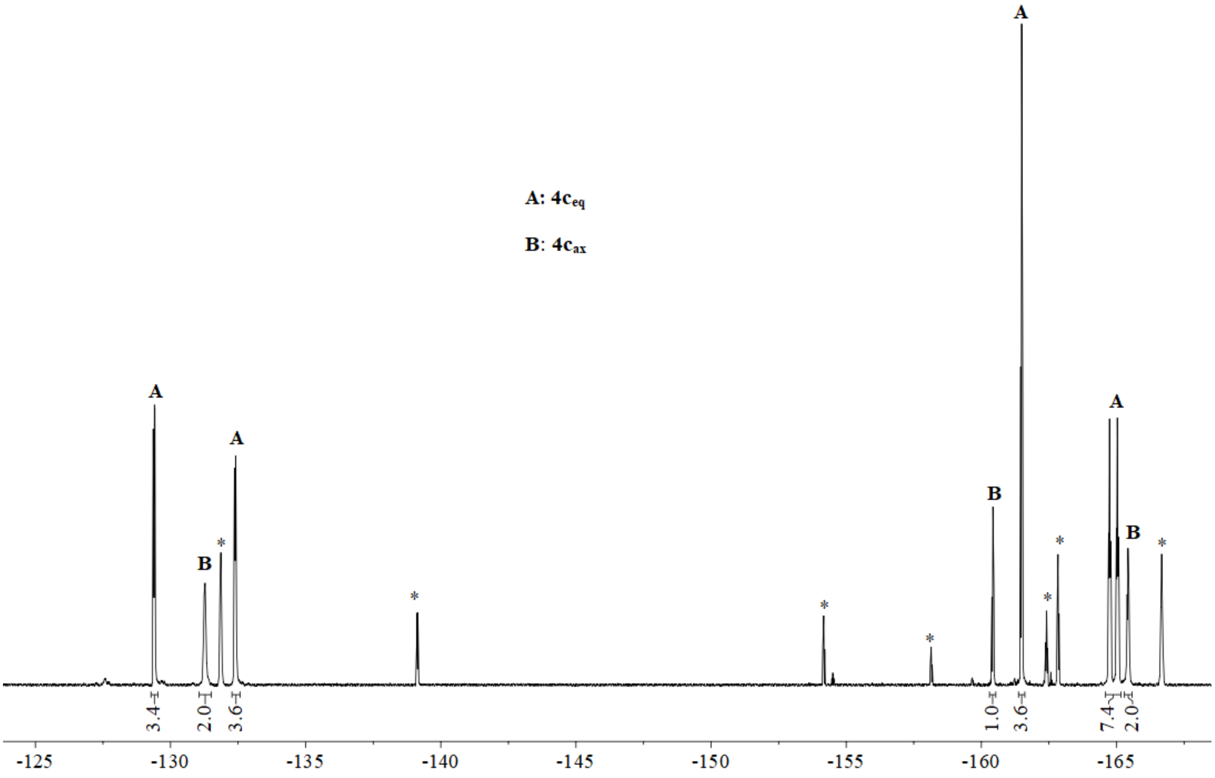

Figure S42. ^{19}F NMR (564 MHz, 299 K, C_6D_6) spectrum of the reaction mixture [step 1]

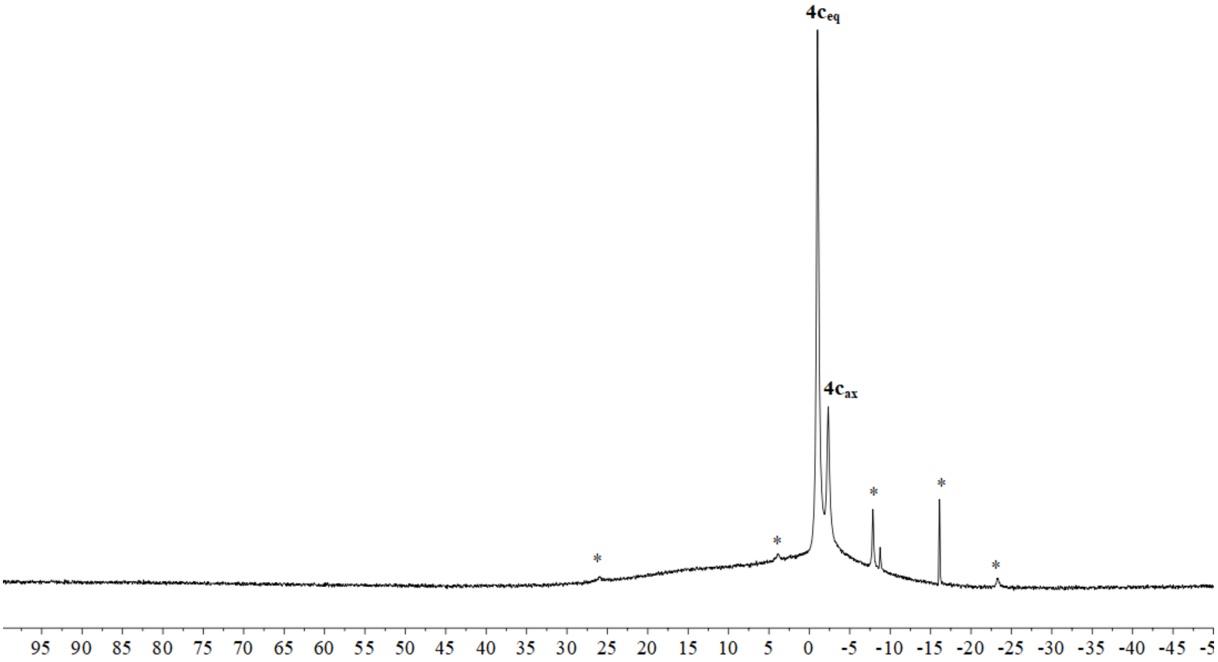

Figure S43. ^{11}B{^{1}H} NMR (192 MHz, 299 K, C_6D_6) spectrum of the reaction mixture [step 1; *: impurities]

Step 2: After that the solvent was removed in vacuo and the residue was extracted with pentane (5 mL). The pentane extract was filtered and the obtained solution was dried in vacuo to give a light-yellow oil (29.5 mg, ca. 90%) [*Comment: the isolated compound **4c** contained a mixture of conformational isomers **4c**$_{eq}$/**4c**$_{ax}$ (ca 3:1) due to the orientation of the Me^{CHC6H3} group at the 7-membered ring core*]. Then, a solution of the **4c**$_{eq}$/**4c**$_{ax}$ mixture was prepared in CD_2Cl_2 and kept at r.t. (18 h + 26 h) and followed at 50 °C (oil bath, 18 h). The corresponding ^1H, ^{19}F, and ^{11}B NMR spectra were recorded (see Figure S44-S46).

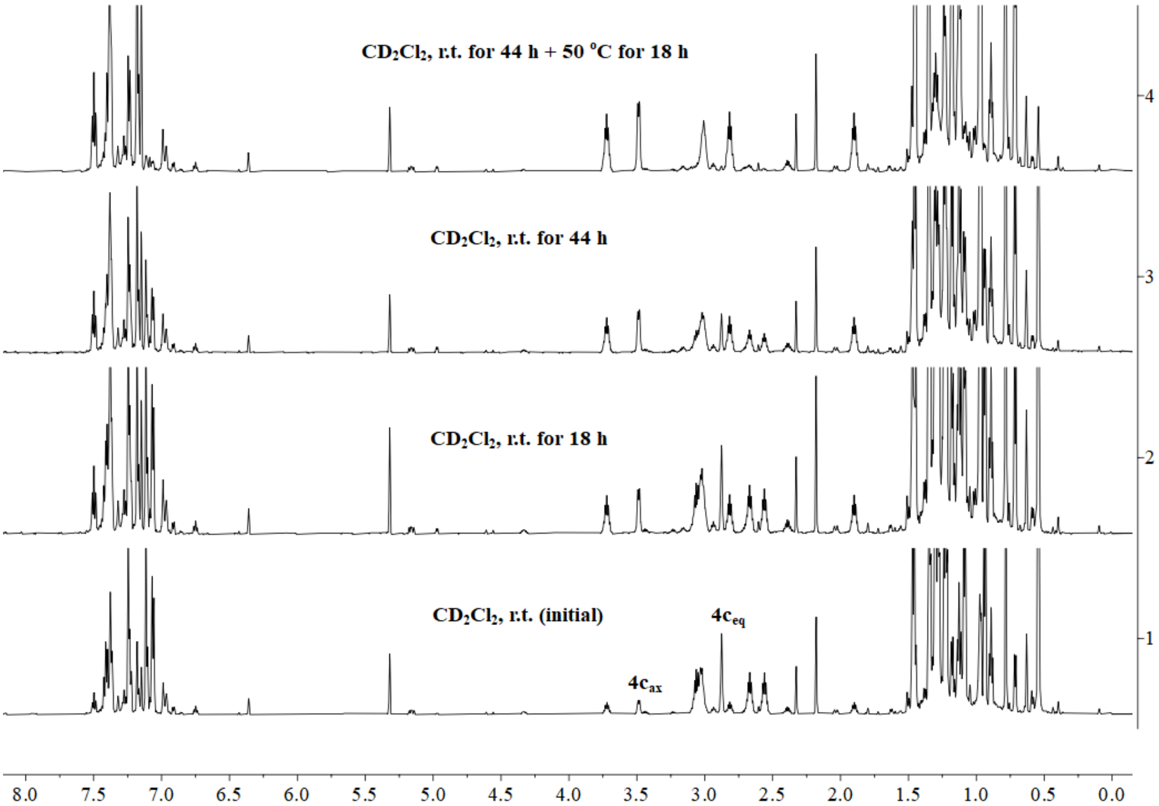

Figure S44. (1,2,3,4) ^1H NMR (600 MHz, 299 K, CD_2Cl_2) spectra of compound **4c** [step 2]

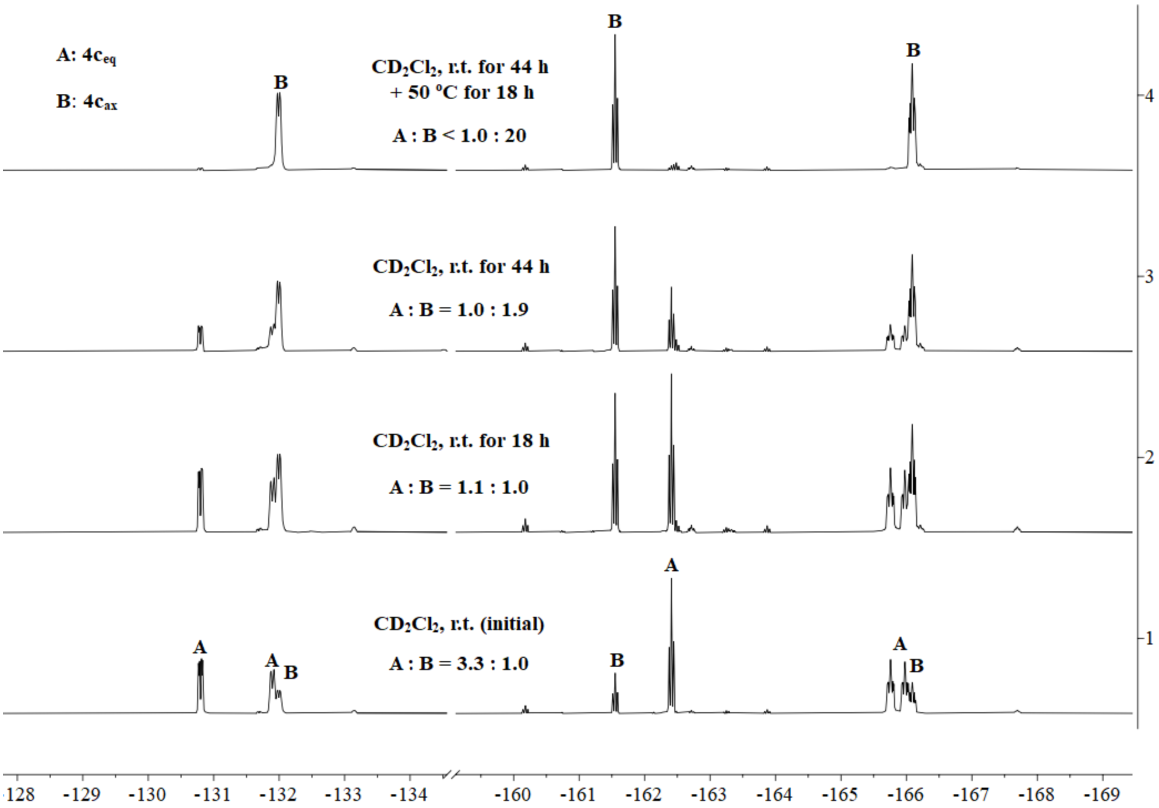

Figure S45. (1,2,3,4) ^{19}F NMR (564 MHz, 299 K, CD_2Cl_2) spectra of compound **4c** [step 2]

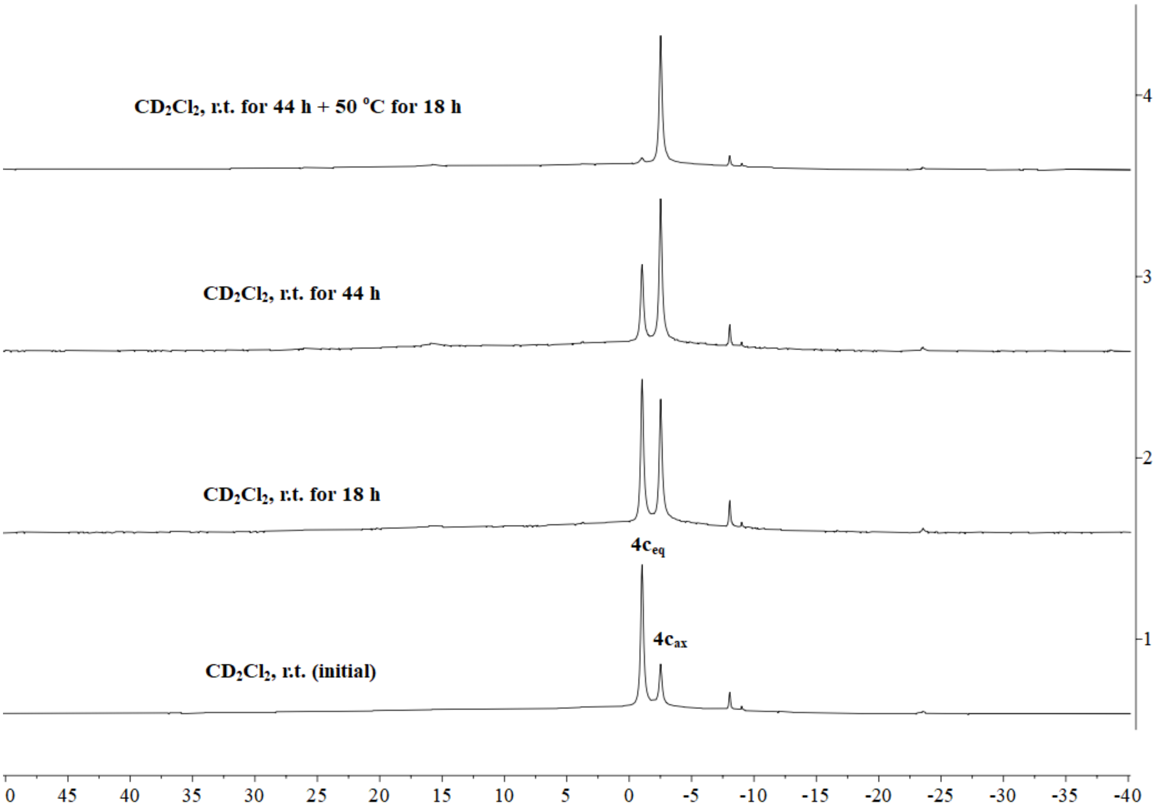

Figure S46. (1,2,3,4) $^{11}B\{^1H\}$ NMR (192 MHz, 299 K, CD_2Cl_2) spectra of compound **4c** [step 2]

2nd Experiment: Compound **3** (5.6 mg, 0.01 mmol, 1.0 equiv.) and [^{t}BuNO]$_2$ (1.7 mg, 0.01 mmol, 1.0 equiv. (2.0 equiv. "NO")) were mixed and toluene (1.0 mL) was added. The resulting mixture was stirred at r.t. for 1.5 h and then used for the EPR experiment (see Figure S47).[20]

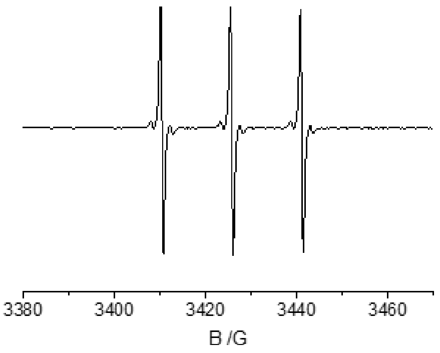

Figure S47. Liquid-state CW EPR spectrum of the reaction mixture recorded in toluene at X-band and at room temperature.

Table S4. g-factor and hyperfine coupling constants (in G) for the reaction mixture, yielding the optimum agreement with the experimental CW EPR spectrum.

Nuclei	*g*-factor	A(^{14}N)
^{14}N	2.00688	15.37 G

3rd Experiment: Compound **3** (56.3 mg, 0.1 mmol, 1.0 equiv.) and [^{t}Bu-NO]$_2$ (17.4 mg, 0.1 mmol, 1.0 equiv. (2.0 equiv. "NO")) were mixed and C_6D_6 (1.0 mL) was added. The resulting mixture was stirred at room temperature for 14 h in the dark. Then the solvent was removed in vacuo and the residue was extracted with pentane (5 mL). The pentane extract was filtered and the obtained solution was dried in vacuo to give compound **4c** as light-yellow oil. The obtained oil was dissolved with CH_2Cl_2 (0.5 mL) and the solution was heated at 50 °C (oil bath) for 24 hours. After that, heptane (2 mL) was added and the resulting suspension was filtered. The obtained solution was kept at -35 °C for 5 days to give compound **4c$_{ax}$** as a white crystalline material (ca. 50 mg, 77%). Some of the obtained crystals were suitable for the X-ray single crystal structure analysis.

Characterization of compound 4c$_{ax}$:

Elemental analysis (%) calc. for $C_{37}H_{43}BF_5N_3O$: C, 68.21; H, 6.65; N, 6.45. Found: C, 68.26; H, 6.98; N, 6.44.

NMR data of compound **4c$_{ax}$** were obtained from a solution of the obtained white solid in CD_2Cl_2 at r.t. [Dipp: 2,6-diisopropylphenyl]

^{1}H NMR (600 MHz, 299 K, CD_2Cl_2) δ {7.49 (t, $^3J_{HH}$ = 7.8 Hz, 1H, *p*-), [7.37, 7.24](each d, $^3J_{HH}$ = 7.8 Hz, each 1H, *m*-)}(Dipp), [7.40 (*p*-), 7.37 (*m*-), 7.17 (*m'*-)](each m, each 1H,

C_6H_3), [7.18, 7.15] (each s, each 1H, =CH), [3.71 (sept, $^3J_{HH}$ = 6.7 Hz, 1H), 1.45 (dd, $^3J_{HH}$ = 6.7 Hz, 3H), 1.18 (d, $^3J_{HH}$ = 6.7 Hz, 3H)](*i*Pr^{Dipp}), 3.48 (d, *J* = 7.8 Hz, HCB), 3.00 (m, 1H, CH^{CHB}), {2.81 (sept, $^3J_{HH}$ = 6.8 Hz, 1H), [1.35, 0.71](each d, $^3J_{HH}$ = 6.8 Hz, each 3H)}(*i*Pr^{C6H3}), [1.89 (sept, $^3J_{HH}$ = 6.7 Hz, 1H), 0.97 (d, $^3J_{HH}$ = 6.7 Hz, 3H), 0.96 (d, $^3J_{HH}$ = 6.7 Hz, 3H)](*i*Pr^{Dipp}), 1.12 (d, $^3J_{HH}$ = 7.6 Hz, 3H, Me^{CHC6H3}), 0.78 (s, 3H, *t*Bu).

^{13}**C{^{1}H} NMR** (151 MHz, 299 K, CD_2Cl_2) δ 168.5 (br, BC), [147.2, 145.8](*o*-Dipp), [144.5 (*o*-), 143.0 (*o'*-)](C_6H_3), 135.0 (*i*-C_6H_3), 134.4 (*i*-Dipp), 130.5 (*p*-Dipp), 129.9 (*p*-C_6H_3), [128.7 (*m'*-), 125.4 (*m*-)](C_6H_3), [125.5, 123.6](=CH), [124.3, 123.4](*m*-Dipp), 61.7 (br m, CHB), 58.7 (*t*Bu), 43.9 (CH^{CHB}), [29.0 (CH), 26.4 (Me), 21.3 (Me)](*i*Pr^{Dipp}), [28.9 (CH), 25.7 (Me), 22.8 (Me)](*i*Pr^{Dipp}), 28.5 (*o*-CH^{iPr}(C_6H_3)), 24.2 (*t*Bu), [23.9, 22.7](*o*-Me^{iPr}(C_6H_3)), 14.2 (Me^{CHC6H3}), [C_6F_5 not listed].

^{11}**B{^{1}H} NMR** (192 MHz, 299 K, CD_2Cl_2) δ -2.5 ($\nu_{1/2}$ ≈ 45 Hz).

^{19}**F NMR** (564 MHz, 299 K, CD_2Cl_2) δ -132.0 (m, 2F, *o*-C_6F_5), -161.6 (t, $^3J_{FF}$ = 19.7 Hz, *p*-C_6F_5), -166.1 (m, 2F, *m*-C_6F_5), [$\Delta\delta^{19}F_{m,p}$ = 4.5].

^{1}**H,**^{13}**C gHMBC** (600 MHz/151 MHz, 299 K, CD_2Cl_2)[selected traces] δ ^{1}H/^{13}C {[7.18/168.5, 125.4], [7.15/168.5, 123.6]}(=CH/BC, =CH), [3.48/168.5, 121.8, 58.7, 14.2](HCB/BC, *i*-C_6F_5, C^{tBu}, Me^{CHC6H3}), [3.00/143.0, 135.0, 128.7, 61.7, 14.2](CH^{CHB}/*o'*-C_6H_3, *i*-C_6H_3, *m'*-C_6H_3, CHB, Me^{CHC6H3}).

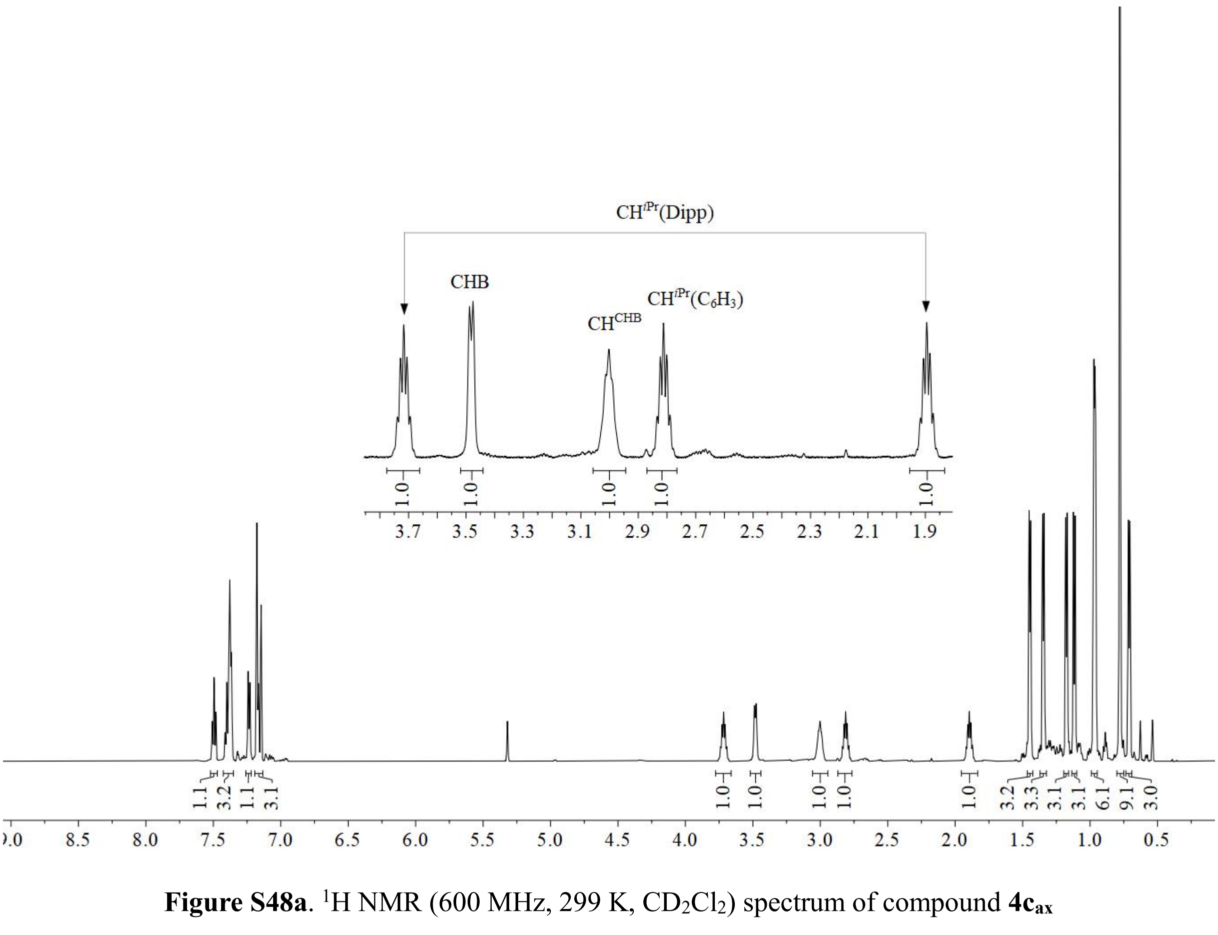

Figure S48a. ^{1}H NMR (600 MHz, 299 K, CD_2Cl_2) spectrum of compound **4c$_{ax}$**

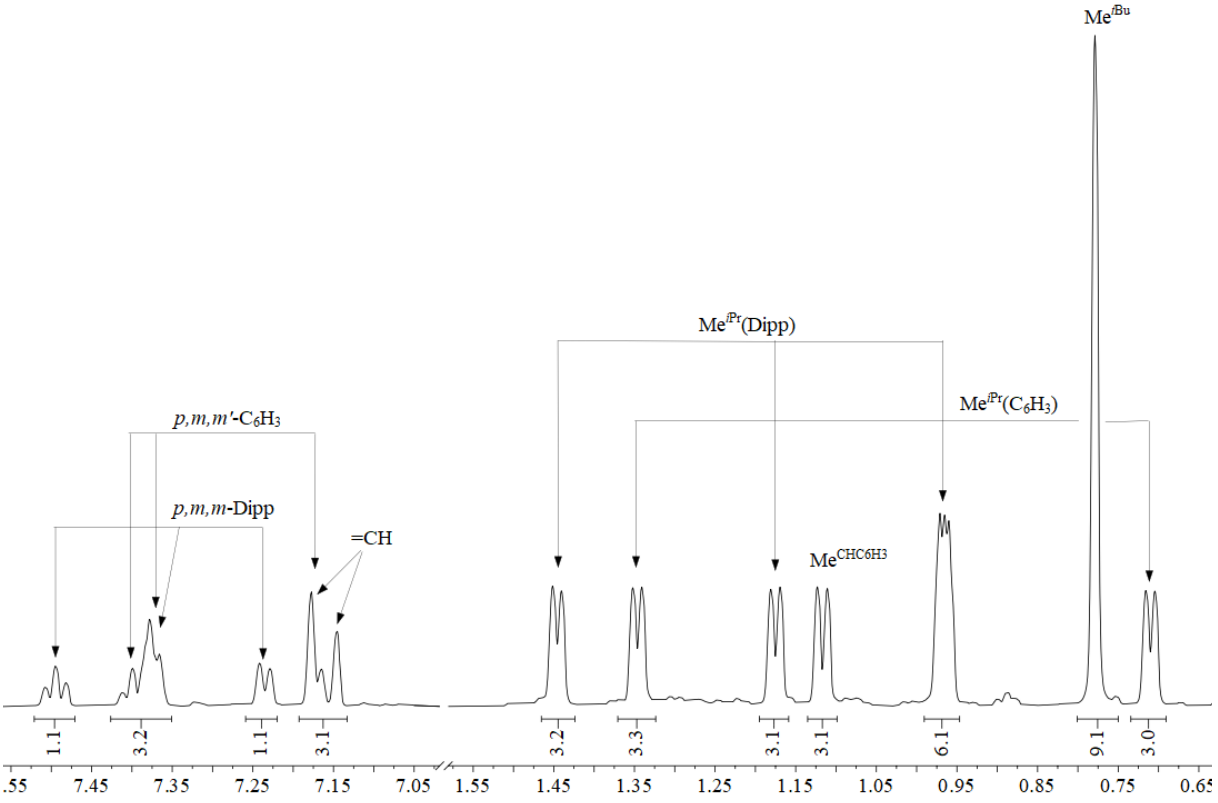

Figure S48b. ^{1}H NMR (600 MHz, 299 K, CD_2Cl_2) spectrum of compound **4c$_{ax}$**

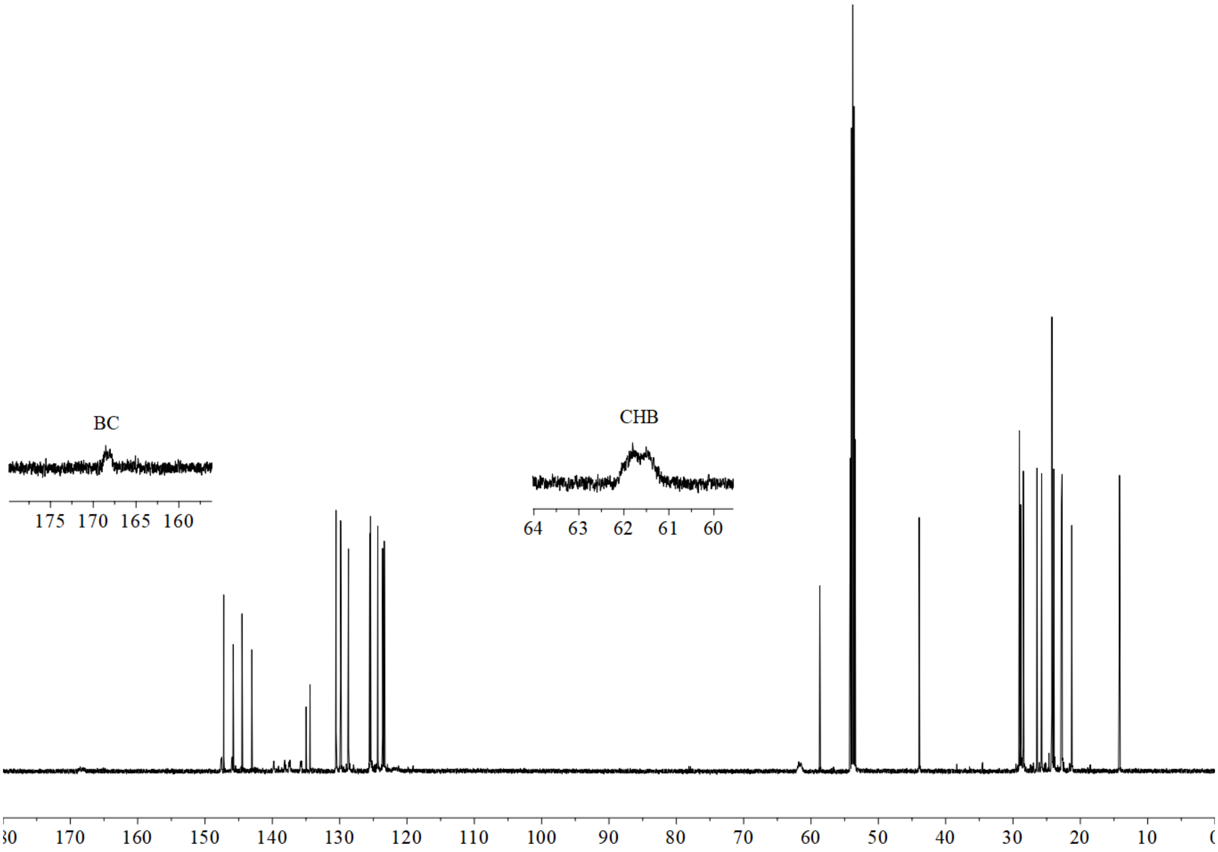

Figure S49a. $^{13}C\{^{1}H\}$ NMR (151 MHz, 299 K, CD_2Cl_2) spectrum of compound **4c$_{ax}$**

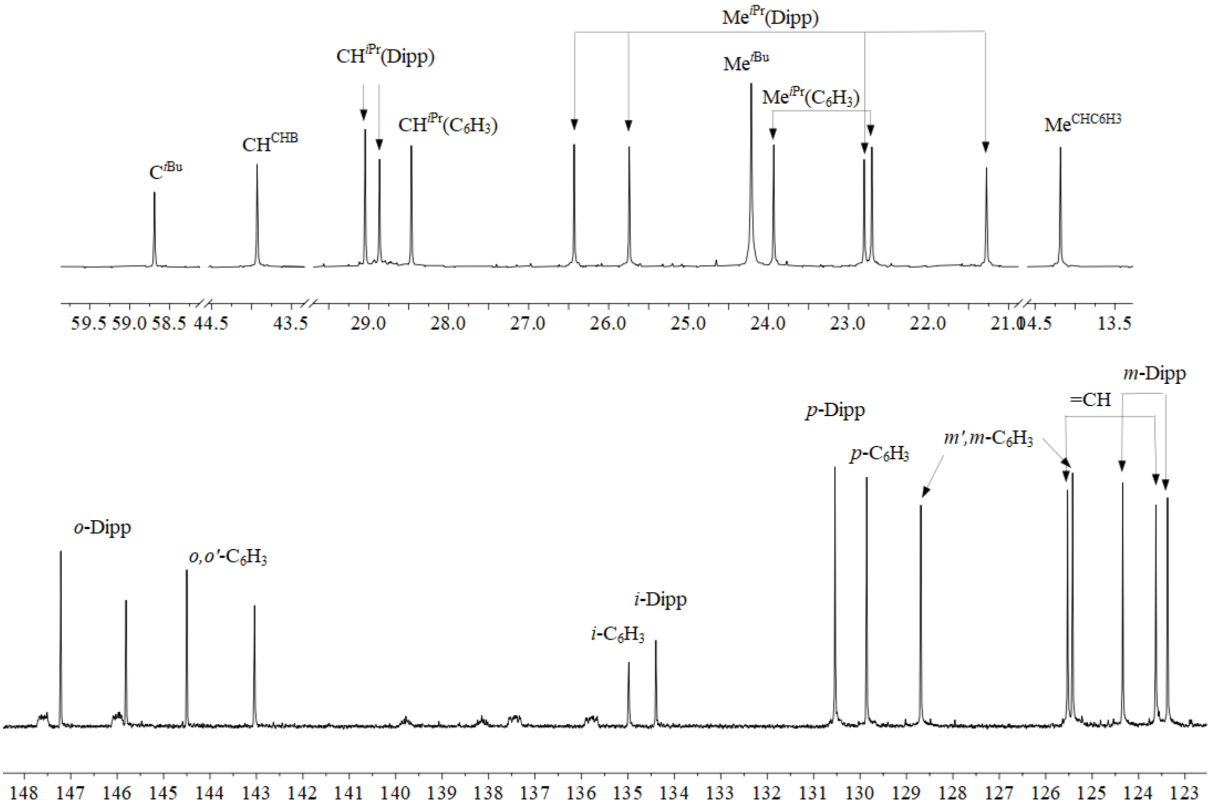

Figure S49b. $^{13}C\{^1H\}$ NMR (151 MHz, 299 K, CD_2Cl_2) spectrum of compound $\mathbf{4c_{ax}}$

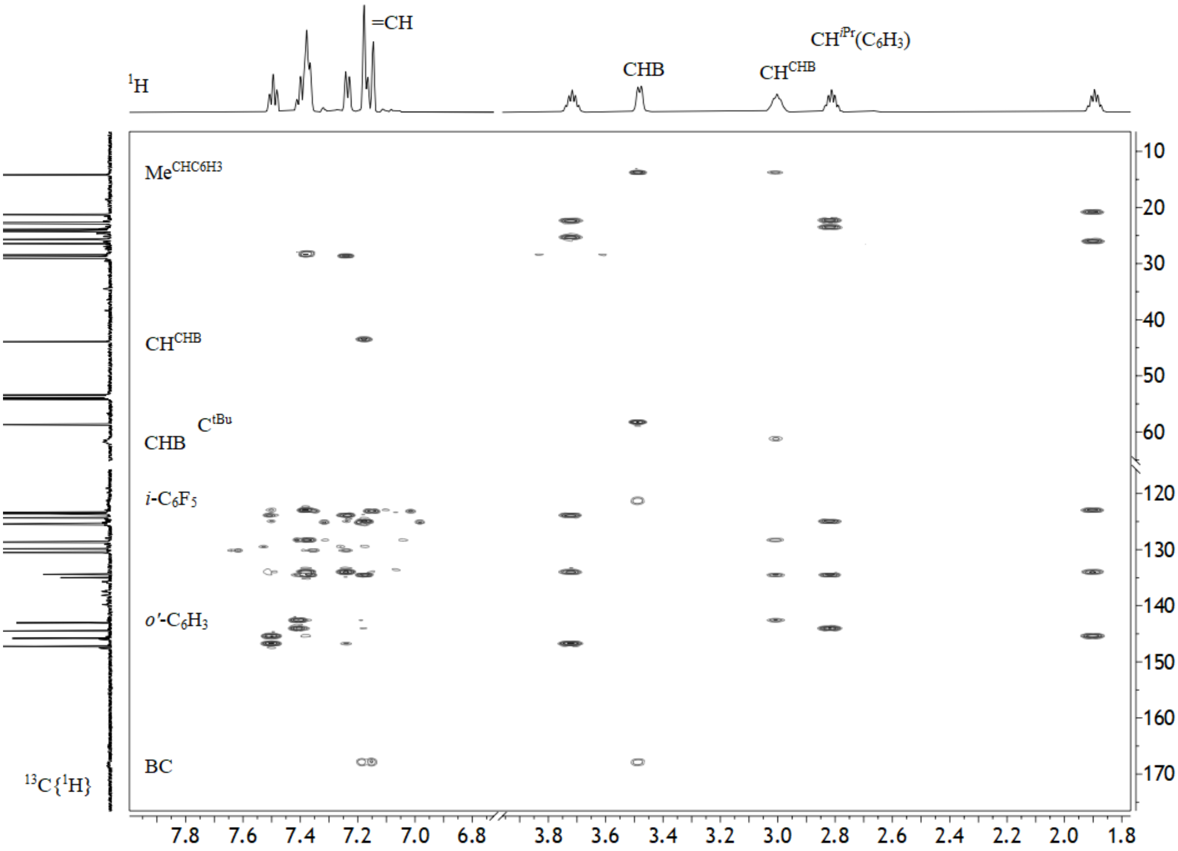

Figure S50. $^1H,^{13}C$ gHMBC (600/151 MHz, 299 K, CD_2Cl_2) spectrum of compound $\mathbf{4c_{ax}}$

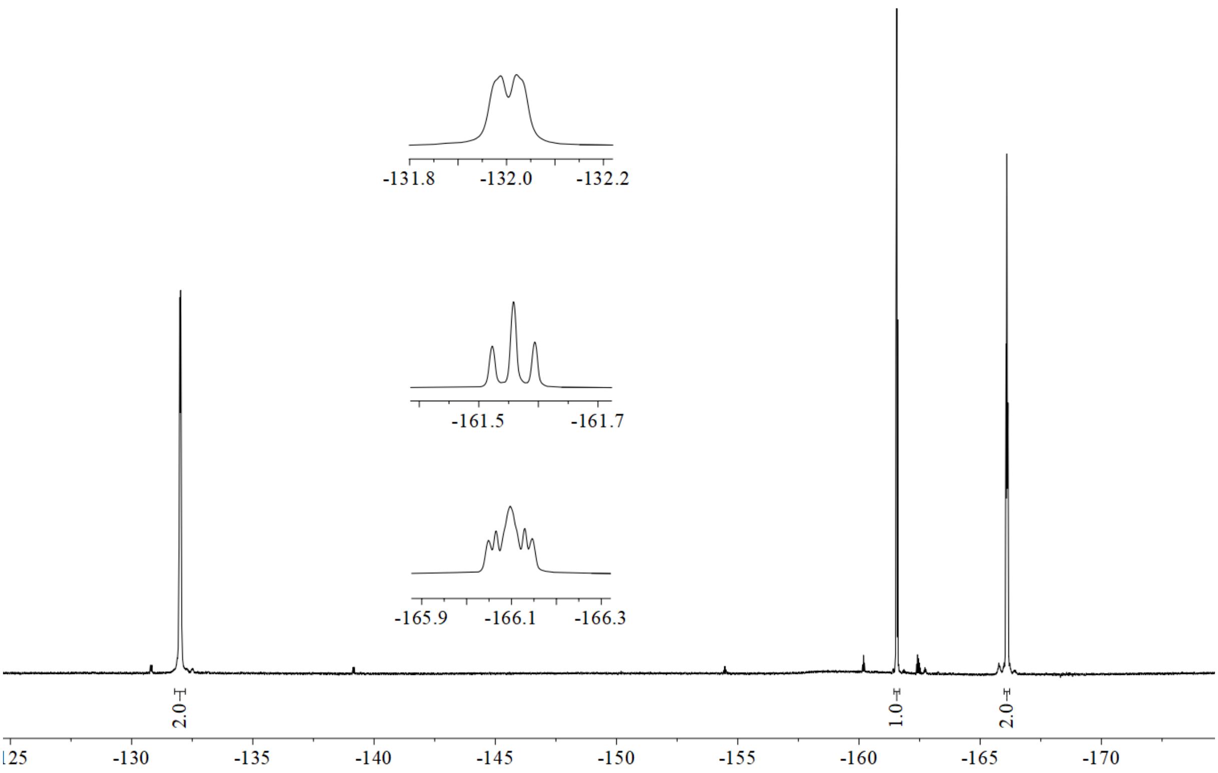

Figure S51. ^{19}F NMR (564 MHz, 299 K, CD_2Cl_2) spectrum of compound **4c$_{ax}$**

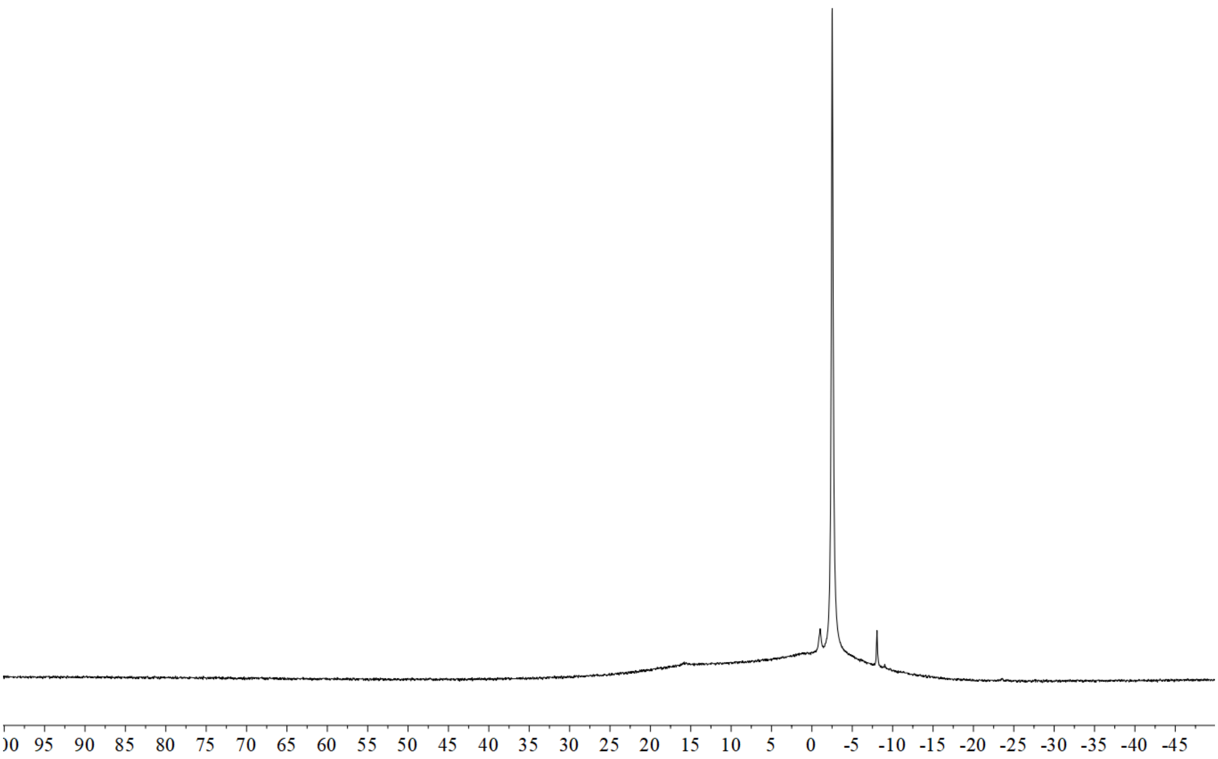

Figure S52. ^{11}B{^{1}H} NMR (192 MHz, 299 K, CD_2Cl_2) spectrum of compound **4c$_{ax}$**

X-ray crystal structure analysis of compound 4c$_{ax}$ (ERK10356: A colorless, plate-like specimen of $C_{37}H_{43}BF_5N_3O$, approximate dimensions 0.084 mm x 0.100 mm x 0.188 mm,

was used for the X-ray crystallographic analysis. The X-ray intensity data were measured on a single crystal diffractometer Bruker D8 Venture Photon III system equipped with a micro focus tube Mo ImS (MoKα, λ = 0.71073 Å) and a MX mirror monochromator. A total of 784 frames were collected. The total exposure time was 6.50 hours. The frames were integrated with the Bruker SAINT software package using a narrow-frame algorithm. The integration of the data using a triclinic unit cell yielded a total of 48227 reflections to a maximum θ angle of 25.35° (0.83 Å resolution), of which 6791 were independent (average redundancy 7.102, completeness = 99.8%, R_{int} = 9.63%, R_{sig} = 5.22%) and 4794 (70.59%) were greater than $2\sigma(F^2)$. The final cell constants of a = 9.2846(4) Å, b = 10.7049(5) Å, c = 19.0761(8) Å, α = 94.642(2)°, β = 99.478(2)°, γ = 93.573(2)°, volume = 1858.44(14) $Å^3$, are based upon the refinement of the XYZ-centroids of 4736 reflections above 20 σ(I) with 4.461° < 2θ < 51.94°. Data were corrected for absorption effects using the Multi-Scan method (SADABS). The ratio of minimum to maximum apparent transmission was 0.917. The calculated minimum and maximum transmission coefficients (based on crystal size) are 0.9840 and 0.9930. The structure was solved and refined using the Bruker SHELXTL Software Package, using the space group *P*-1, with Z = 2 or the formula unit, $C_{37}H_{43}BF_5N_3O$. The final anisotropic full-matrix least-squares refinement on F^2 with 434 variables converged at R1 = 4.60%, for the observed data and wR2 = 12.94% for all data. The goodness-of-fit was 1.030. The largest peak in the final difference electron density synthesis was 0.241 $e^-/Å^3$ and the largest hole was -0.255 $e^-/Å^3$ with an RMS deviation of 0.048 $e^-/Å^3$. On the basis of the final model, the calculated density was 1.164 g/cm^3 and F(000), 688 e^-. CCDC Nr.: 2182533.

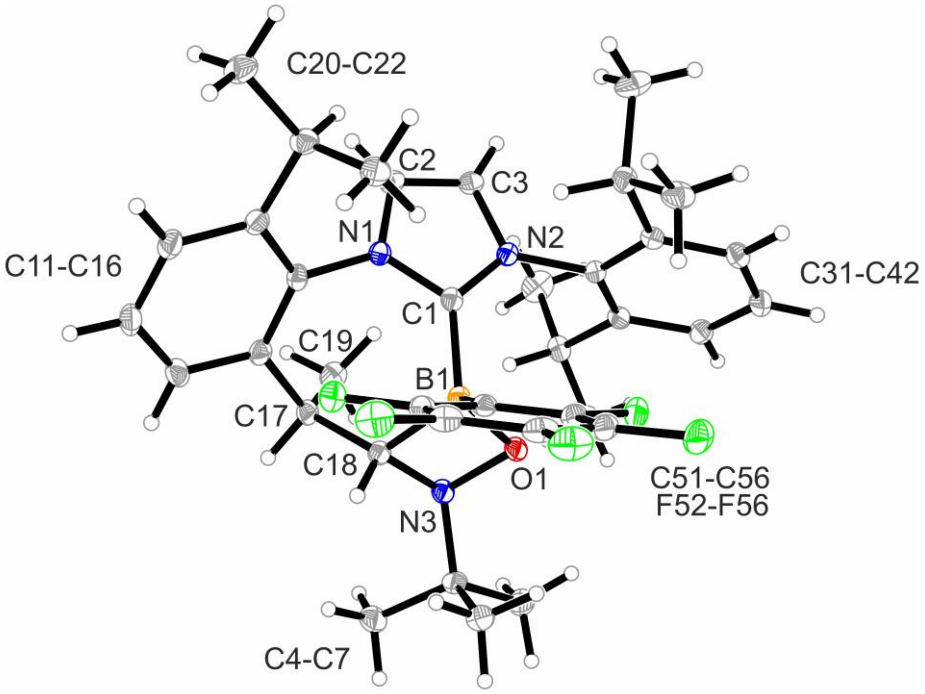

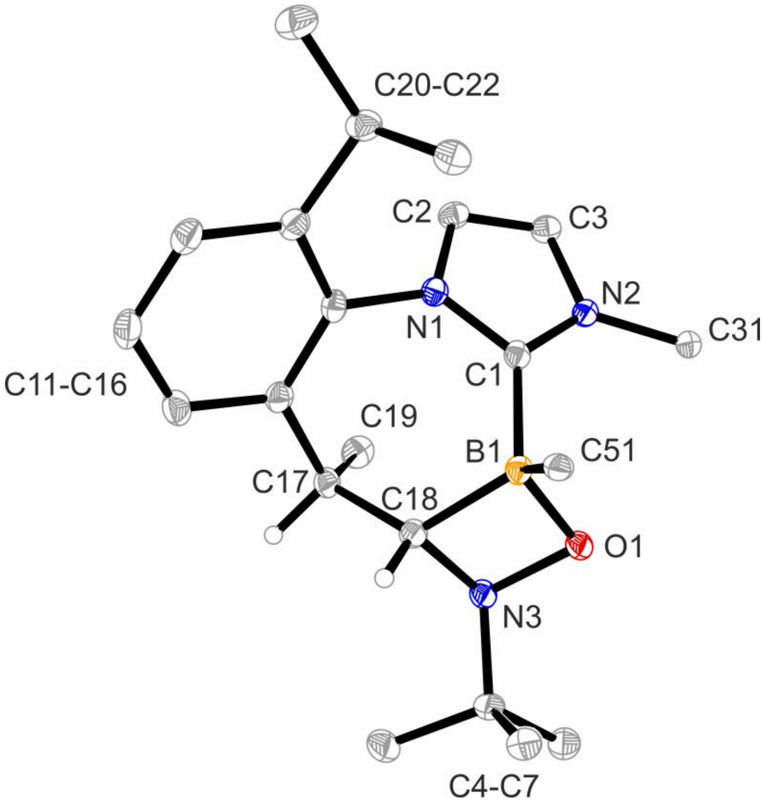

Figure S53. Crystal structure of compound **4c$_{ax}$** (thermal ellipsoids: 30% probability)

9. Synthesis and characterization of compound 9

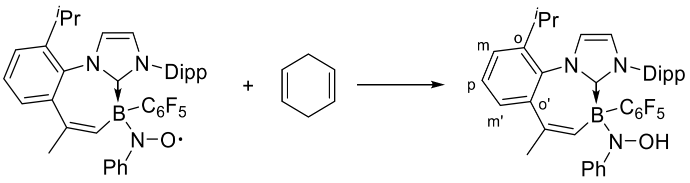

5a **9**

In an NMR tube, compound **5a** (13.4 mg, 0.02 mmol, 1.0 equiv.) was dissolved in C_6D_6 (0.5 mL) and then cyclohexadiene (1 drop, excess) was added to the solution. The NMR tube was shaken for 5 min and kept at r.t. for 3 days. The crystalline solid formed was isolated and washed with pentane (3 × 0.5 mL). Compound **9** (11 mg, 82%) was obtained as a light-yellow crystalline solid after dried in vacuo. Some of the crystals were suitable for the X-ray single crystal structure analysis of compound **9**.

Elemental analysis (%) calc. for $C_{39}H_{39}BF_5N_3O$: C, 69.75; H, 5.85; N, 6.26. Found: C, 69.31; H, 5.77; N, 6.11.

NMR data of compound **9** were obtained from a solution of the obtained crystalline solid in CD_2Cl_2 at r.t. [Dipp: 2,6-diisopropylphenyl]

^{1}**H NMR** (600 MHz, 299 K, CD_2Cl_2) δ 7.53 (t, $^3J_{HH}$ = 7.8 Hz, 1H, *p*-Dipp), [7.38, 7.25](each dd, $^3J_{HH}$ = 7.8 Hz, $^3J_{HH}$ = 1.2 Hz, each 1H, *m*-Dipp), [7.27 (*m'*-), 7.12 (*m*-)](each dd, $^3J_{HH}$ = 7.8 Hz, $^4J_{HH}$ = 1.2 Hz, each 1H, C_6H_3), 7.22 (t, $^3J_{HH}$ = 7.8 Hz, 1H, *p*-C_6H_3), [7.17, 6.94] (each d, $^3J_{HH}$ = 1.8 Hz, each 1H, =CH), [6.94 (m, 2H, *m*-), 6.57 (m, 2H, *o*-), 6.56 (m, 1H, *p*-)](Ph), 6.65 (s, 1H, HCB), 3.36 (s, 1H, OH), {3.05 (sept, $^3J_{HH}$ = 6.7 Hz, 1H), [1.39, 1.14](each d, $^3J_{HH}$ = 6.6 Hz, each 3H)}(*i*Pr^{C6H3}), {2.84 (sept, $^3J_{HH}$ = 6.7 Hz, 1H), [1.32, 1.27](each d, $^3J_{HH}$ = 6.7 Hz, each 3H)}(*i*Pr^{Dipp}), {2.82 (sept, $^3J_{HH}$ = 6.8 Hz, 1H), [1.17, 1.00](each d, $^3J_{HH}$ = 6.8 Hz, each 3H)}(*i*Pr^{Dipp}), 2.17 (d, *J* = 1.1 Hz, 3H, Me$^{C=}$).

^{13}**C{^{1}H} NMR** (151 MHz, 299 K, CD_2Cl_2) δ 169.4 (br, BC), [153.8 (*i*-), 127.9 (*m*-), 118.4 (*p*-), 117.4 (*o*-)](Ph), [147.6, 146.1](*o*-Dipp), 144.8 (br, HCB), [142.6 (*o*-), 139.5 (*o'*-)](C_6H_3), 134.2 (*i*-Dipp), 133.7 (m, =C^{Me}), 132.2 (*i*-C_6H_3), 130.9 (*p*-Dipp), 128.4 (*p*-C_6H_3), [125.1 (*m*-), 124.05 (*m'*-)](C_6H_3), [124.2, 124.08](*m*-Dipp), [123.4, 123.0](=CH), [28.9 (m, CH), 26.4 (Me), 22.8 (Me)](*i*Pr^{Dipp}), [28.8 (CH), 26.1 (Me), 21.9 (Me)](*i*Pr^{Dipp}), [28.5 (CH), 26.2 (Me), 23.5 (Me)](*i*Pr^{C6H3}), 25.8 (Me$^{C=}$), [C_6F_5 not listed].

^{11}**B{^{1}H} NMR** (192 MHz, 299 K, CD_2Cl_2) δ -8.5 ($\nu_{1/2}$ ≈ 40 Hz).

^{19}**F NMR** (564 MHz, 299 K, CD_2Cl_2) δ [-128.7, -130.5](each m, each 1F, *o*-C_6F_5), -162.0 (t, $^3J_{FF}$ = 20.3 Hz, *p*-C_6F_5), [-165.4, -166.1](each m, each 1F, *m*-C_6F_5), [$\Delta\delta^{19}F_{m,p}$ = 3.4, 4.1].

^{1}**H,**^{13}**C gHMBC** (600 MHz/151 MHz, 299 K, CD_2Cl_2)[selected traces] δ ^{1}H/^{13}C [6.65/139.5, 133.7, 25.8](HCB/*o'*-C_6H_3, =C^{Me}, Me$^{C=}$), [2.17/144.8, 139.5, 133.7](Me$^{C=}$/HCB, *o'*-C_6H_3, =C^{Me}).

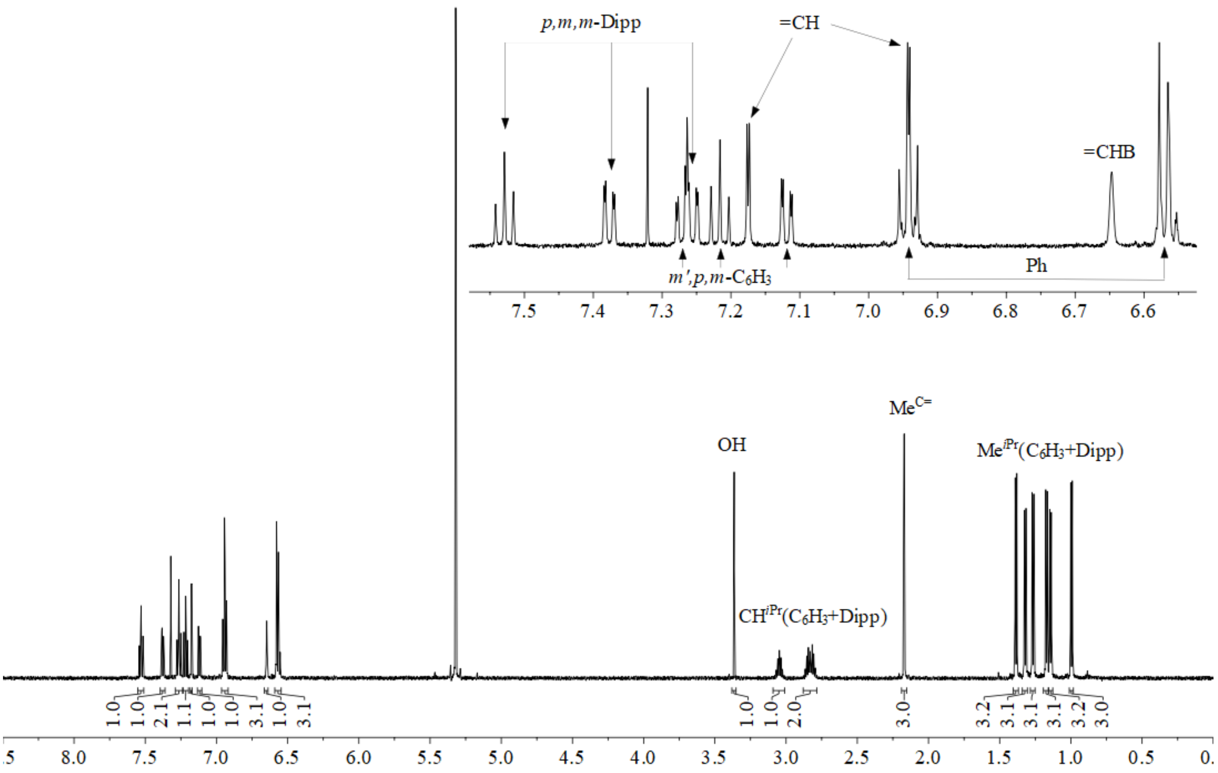

Figure S54. ^{1}H NMR (600 MHz, 299 K, CD_2Cl_2) spectrum of compound **9**

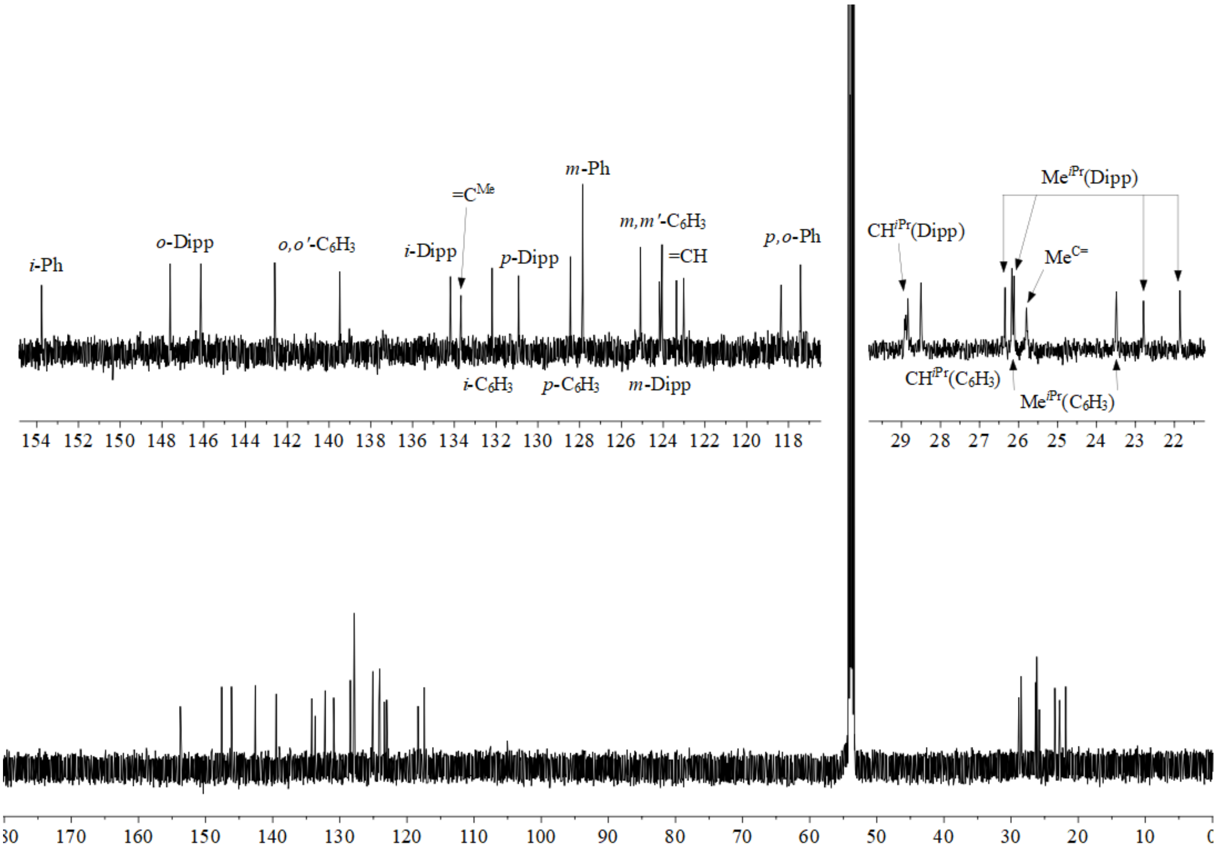

Figure S55. ^{13}C{^{1}H} NMR (151 MHz, 299 K, CD_2Cl_2) spectrum of compound **9**

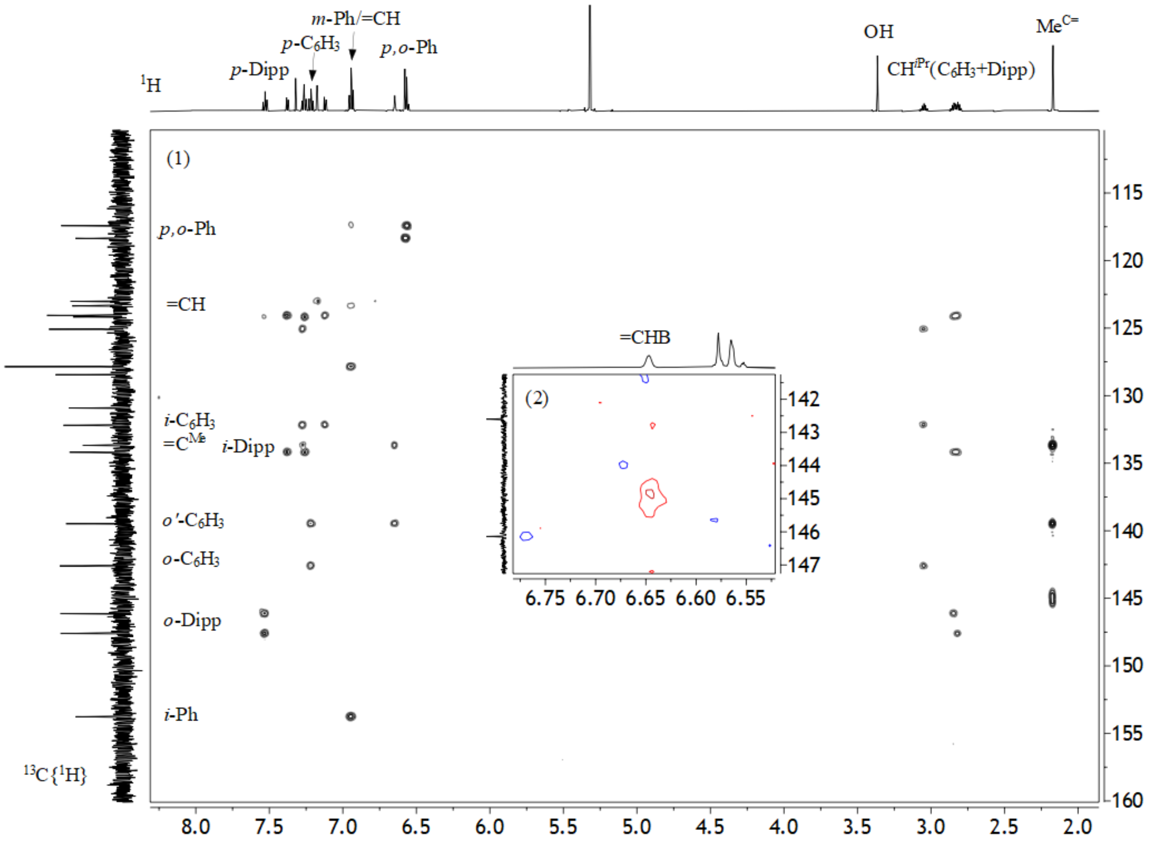

Figure S56. (1) $^{1}H,^{13}C$ gHSQC and (2) $^{1}H,^{13}C$ gHMBC NMR (600/151 MHz, 299 K, CD_2Cl_2) spectra of compound **9** [selected area]

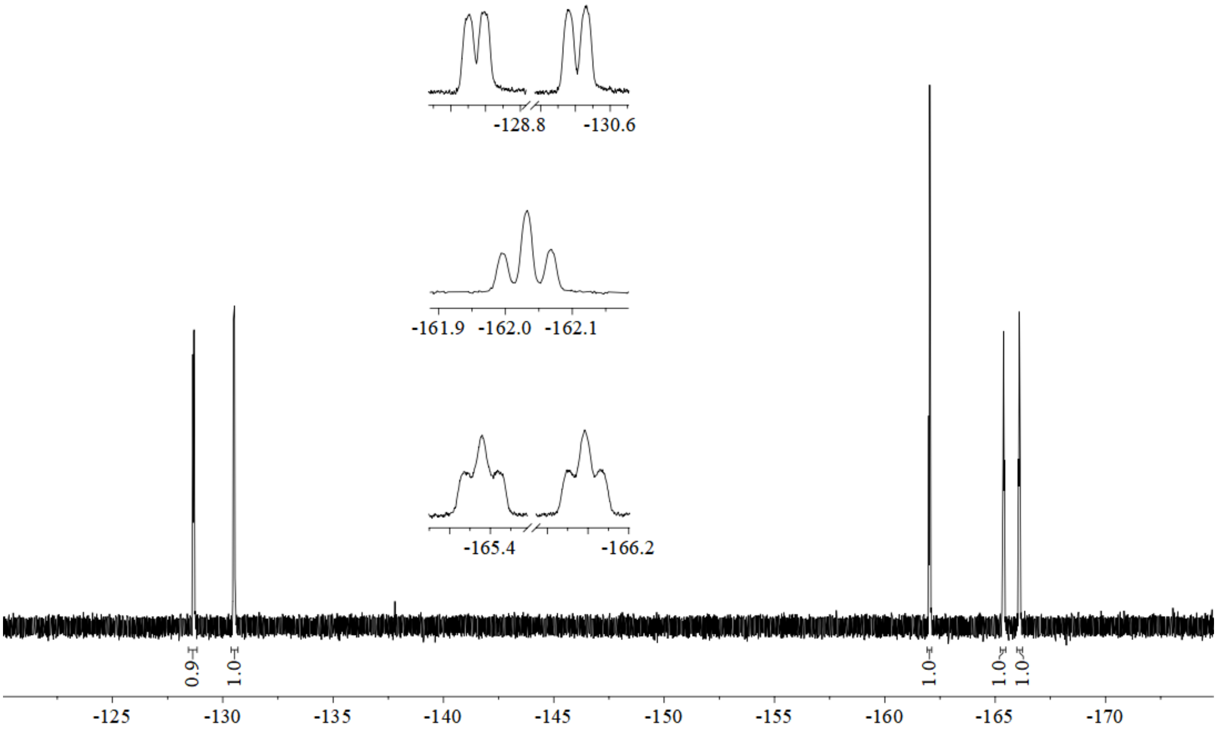

Figure S57. ^{19}F NMR (564 MHz, 299 K, CD_2Cl_2) spectrum of compound **9**

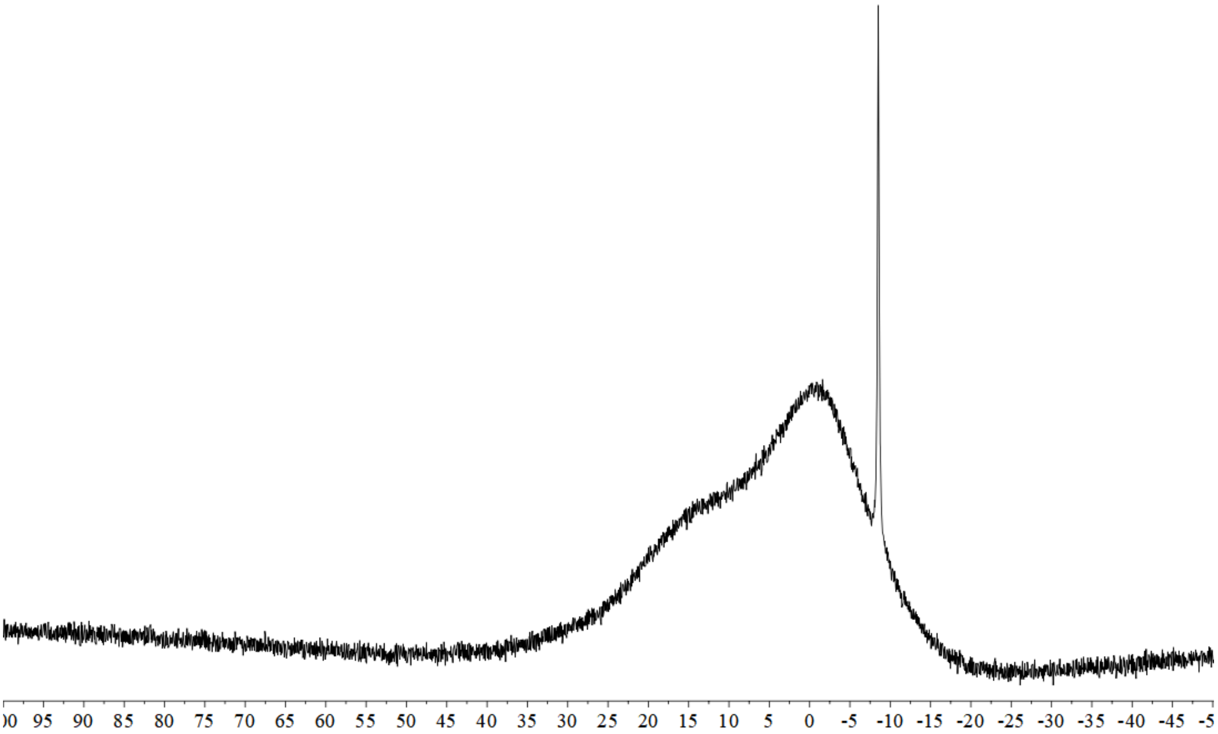

Figure S58. $^{11}B\{^{1}H\}$ NMR (192 MHz, 299 K, CD_2Cl_2) spectrum of compound **9**

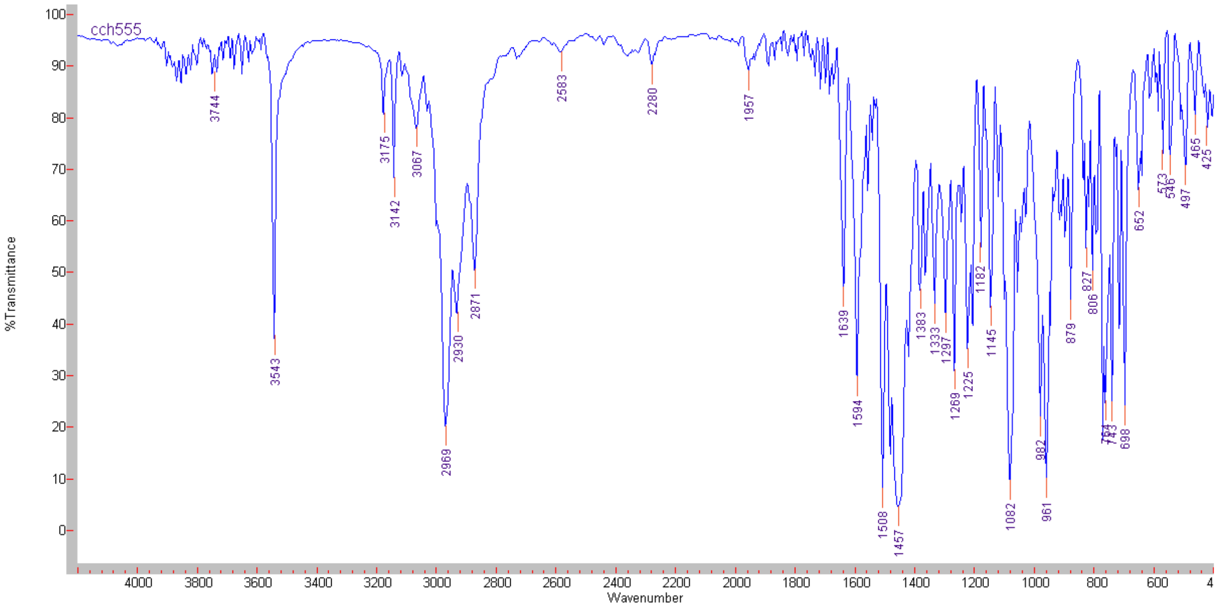

Figure S59. FT-IR spectrum of compound **9** (KBr)

X-ray crystal structure analysis of compound 9 (erk10033): A colorless prism-like specimen of $C_{39}H_{39}BF_5N_3O$, approximate dimensions 0.131 mm x 0.168 mm x 0.416 mm, was used for the X-ray crystallographic analysis. The X-ray intensity data were measured on a Bruker D8 Venture Bruker D8 Venture Photon III Diffractometer system equipped with a micro focus tube MoKα (MoKα, λ = 0.71073 Å) and a MX mirror monochromator. A total of 714 frames were collected. The total exposure time was 6.94 hours. The frames were

integrated with the Bruker SAINT software package using a narrow-frame algorithm. The integration of the data using a monoclinic unit cell yielded a total of 87549 reflections to a maximum θ angle of 26.81° (0.79 Å resolution), of which 7108 were independent (average redundancy 12.317, completeness = 99.6%, R_{int} = 7.49%, R_{sig} = 2.84%) and 6054 (85.17%) were greater than 2σ(F^2). The final cell constants of a = 16.5547(5) Å, b = 10.2914(4) Å, c = 20.8535(8) Å, β = 110.1980(10)°, volume = 3334.4(2) $Å^3$, are based upon the refinement of the XYZ-centroids of 9980 reflections above 20 σ(I) with 4.471° < 2θ < 53.41°. Data were corrected for absorption effects using the multi-scan method (SADABS). The ratio of minimum to maximum apparent transmission was 0.948. The calculated minimum and maximum transmission coefficients (based on crystal size) are 0.9600 and 0.9870. The structure was solved and refined using the Bruker SHELXTL Software Package, using the space group $P2_1/c$, with Z = 4 for the formula unit, $C_{39}H_{39}BF_5N_3O$. The final anisotropic full-matrix least-squares refinement on F^2 with 453 variables converged at R1 = 4.80%, for the observed data and wR2 = 10.25% for all data. The goodness-of-fit was 1.092. The largest peak in the final difference electron density synthesis was 0.329 $e^-/Å^3$ and the largest hole was -0.235 $e^-/Å^3$ with an RMS deviation of 0.047 $e^-/Å^3$. On the basis of the final model, the calculated density was 1.338 g/cm^3 and F(000), 1408 e^-. The hydrogen at O1 atom was refined freely. CCDC Nr.: 2157865.

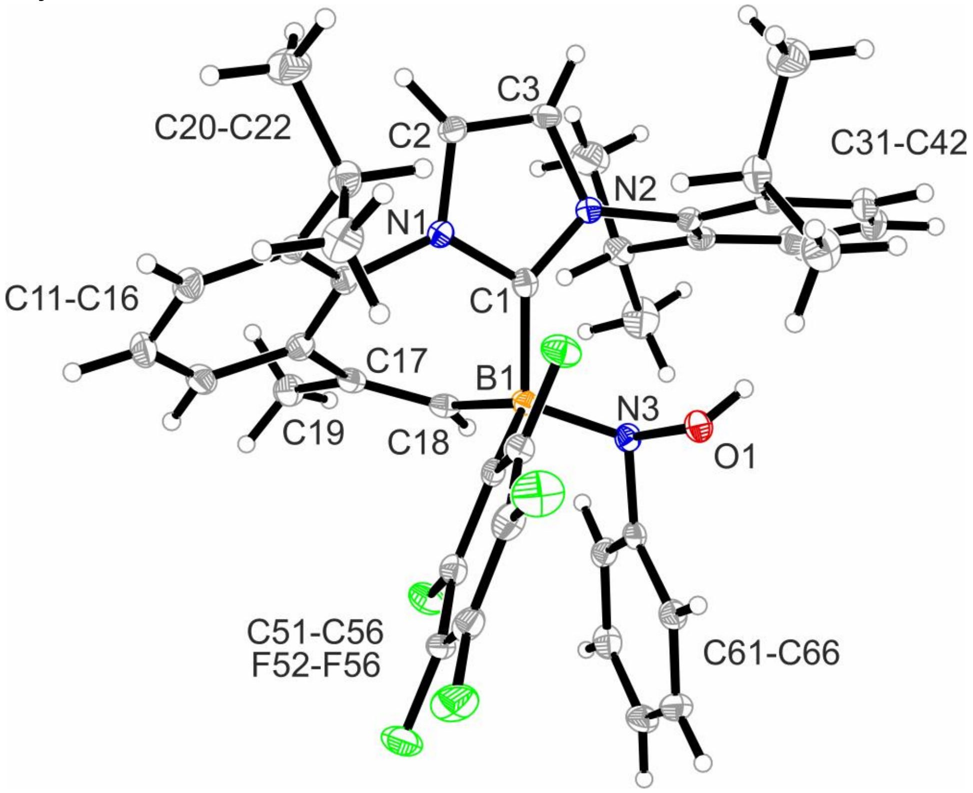

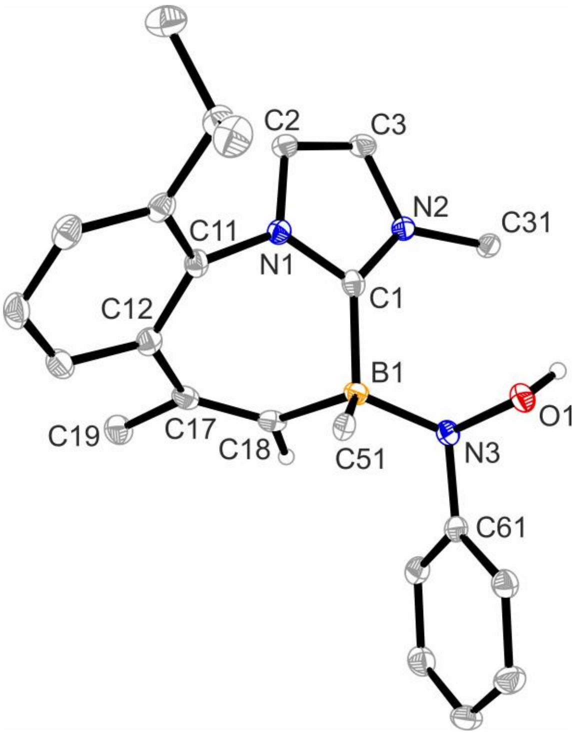

Figure S60. Crystal structure of compound **9** (thermal ellipsoids: 50% probability)

10. Generation of compound 5a from compound 9

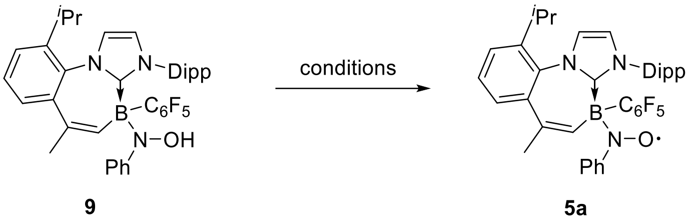

1st Experiment: In a Young NMR tube, compound **9** (6.7 mg, 0.01 mmol, 1.0 equiv.) was suspended in C_6D_6 (0.5 mL). The NMR tube was exposed to air and then rotated at r.t. for 48 hours. The mixture was directly used for ^{1}H NMR experiment (Figure 61(3)).

2nd Experiment: In a Young NMR tube, compound **9** (6.7 mg, 0.01 mmol, 1.0 equiv.) and PhNO (2.2 mg, 0.02 mmol, 2.0 equiv.) were mixed and C_6D_6 (0.5 mL) was added. The NMR tube was rotated at r.t. for 48 hours and the mixture was directly used for ^{1}H NMR experiment (Figure S61(4)).

[*Comment: crystalline compound **9** has extremely poor solubility in C_6D_6, which would cause very slow reaction rate at r.t.*]

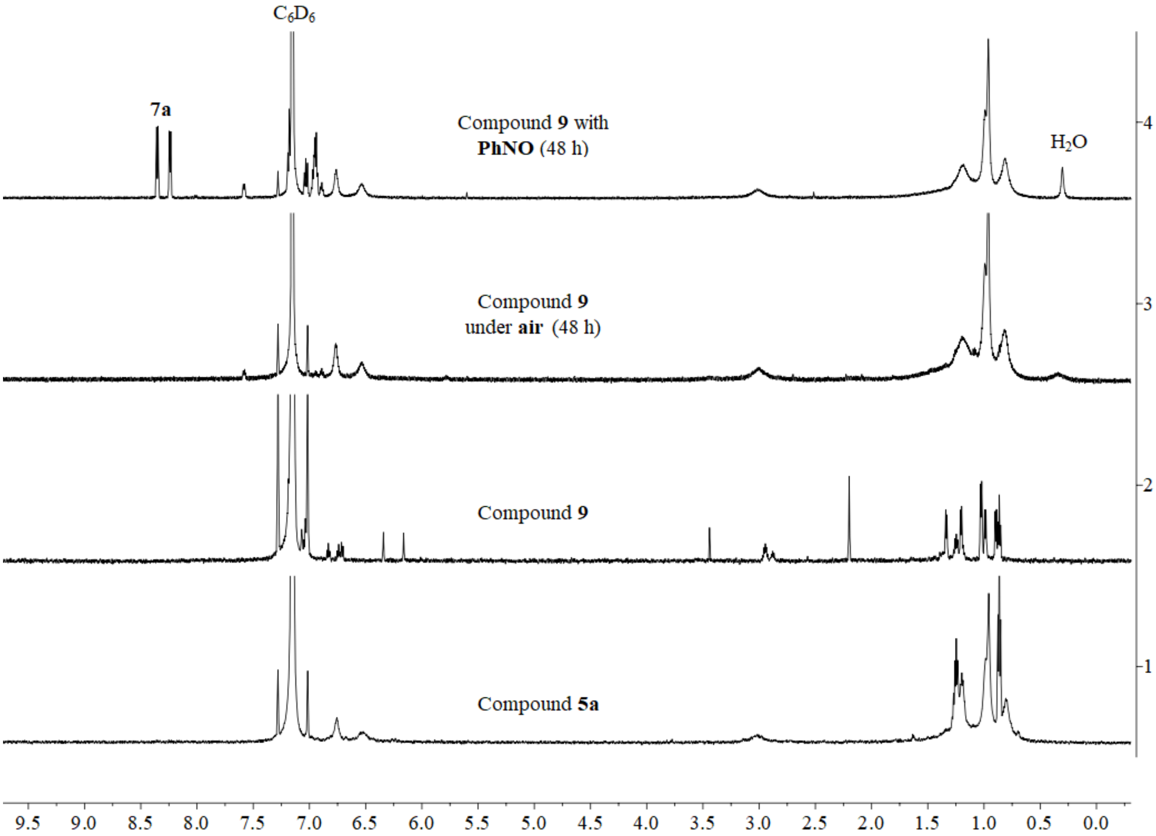

Figure S61. (1,2,3,4) ^{1}H NMR (600 MHz, 299 K, C_6D_6) spectra of (1) compound **5a**, (2) compound **9** (3) **1st Experiment**, and (4) **2nd Experiment**

11. Synthesis and characterization of compound 13

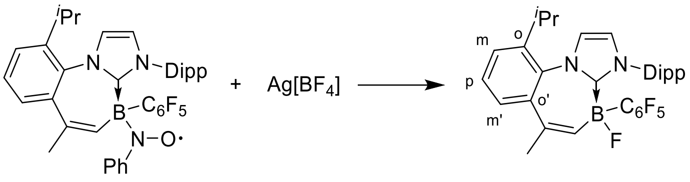

5a **13**

Compound **5a** (33.5 mg, 0.05 mmol, 1.0 equiv.) and Ag[BF_4] (11.0 mg, 0.055 mmol, 1.1 equiv.) were mixed and CH_2Cl_2 (1.0 mL) was added. The mixture was stirred at r.t. for 30 min. Then the resulting suspension was purified by flash chromatography (SiO_2, eluent CH_2Cl_2/pentane 1:1). Compound **13** (20 mg, 69%) was obtained as a white solid after drying in vacuo. Crystals suitable for the X-ray single crystal structure analysis of compound **13** were obtained from a solution of the white solid in pentane.

Elemental analysis (%) calc. for $C_{39}H_{38}B_2F_9N_3O$: C, 68.05; H, 5.71; N, 4.81. Found: C, 67.65; H, 5.75; N, 4.92.

NMR data of compound **13** was obtained from a solution of the obtained white solid in CD_2Cl_2 at r.t. [Dipp: 2,6-diisopropylphenyl]

^{1}H NMR (600 MHz, 299 K, CD_2Cl_2) δ 7.47 (t, $^3J_{HH}$ = 7.8 Hz, 1H, *p*-Dipp), [7.29, 7.27](each ddm, $^3J_{HH}$ = 7.8 Hz, $^4J_{HH}$ = 1.4 Hz, each 1H, *m*-Dipp), [7.23, 6.95](each d, $^3J_{HH}$ = 1.8 Hz, each 1H, =CH), [7.23 (m, 2H, *p*-, *m'*-), 7.20 (m, 1H, *m*-)](C_6H_3), 6.41 (d, *J* = 10.5 Hz, HCB), {3.07 (sept, $^3J_{HH}$ = 6.8 Hz, 1H), [1.42, 1.22](each d, $^3J_{HH}$ = 6.8 Hz, each 3H)}($i\mathrm{Pr}^{C6H3}$), {2.82 (sept, $^3J_{HH}$ = 6.8 Hz, 1H), [1.23, 1.10](each d, $^3J_{HH}$ = 6.8 Hz, each 3H)}($i\mathrm{Pr}^{Dipp}$), {2.40 (sept, $^3J_{HH}$ = 6.8 Hz, 1H), [1.35, 1.12](each d, $^3J_{HH}$ = 6.8 Hz, each 3H)}($i\mathrm{Pr}^{Dipp}$), 2.13 (d, *J* = 1.2 Hz, 3H, $\mathrm{Me}^{C=}$).

^{13}C{^{1}H} NMR (151 MHz, 299 K, CD_2Cl_2) δ 168.2 (br, BC), 150.5 (br, CHB), [146.5 (d, *J* = 2.0 Hz), 145.9 (d, *J* = 1.4 Hz)](*o*-Dipp), [143.4 (*o*-), 139.2 (*o'*-)](*o*-C_6H_3), 133.9 (d, *J* = 0.6 Hz, *i*-Dipp), 133.0 (d, *J* = 12.4 Hz, =C), 131.4 (*i*-C_6H_3), 130.3 (*p*-Dipp), 128.6 (*p*-C_6H_3), [125.1 (*m*-), 124.6 (m'-)](C_6H_3), [124.1, 124.0](*m*-Dipp), [123.8, 122.8](=CH), [28.8 (CH), 26.0 (Me), 23.85 Me)]($i\mathrm{Pr}^{C6H3}$), [28.7 (m, CH), 24.8 (Me), 23.83 (Me)]($i\mathrm{Pr}^{Dipp}$), [28.7 (m, CH), 23.4 (Me), 25.62 (Me)]($i\mathrm{Pr}^{Dipp}$), 25.59 ($\mathrm{Me}^{C=}$), [C_6F_5 not listed].

^{11}B NMR (192 MHz, 299 K, CD_2Cl_2) δ -2.6 (d, J_{FB} ≈ 45 Hz).

^{11}B{^{1}H} NMR (192 MHz, 299 K, CD_2Cl_2) δ -2.6 (d, J_{FB} ≈ 45 Hz).

^{19}F NMR (564 MHz, 299 K, CD_2Cl_2) δ -132.8 (m, 2F, *o*-C_6F_5), -160.5 (t, $^3J_{FF}$ = 20.0 Hz, *p*-C_6F_5), -165.6 (m, 2F, *m*-C_6F_5) [$\Delta\delta^{19}\mathrm{F}m,p$ = 5.1], 205.7 (br m, J_{BF} ≈ 45 Hz, 1F, BF).

^{19}F{^{11}B} NMR (564 MHz, 299 K, CD_2Cl_2) δ -132.8 (m, 2F, *o*-C_6F_5), -160.5 (t, $^3J_{FF}$ = 20.0 Hz, *p*-C_6F_5), -165.6 (m, 2F, *m*-C_6F_5) [$\Delta\delta^{19}\mathrm{F}m,p$ = 5.1], 205.7 (d, *J* = 8.6 Hz, 1F, BF).

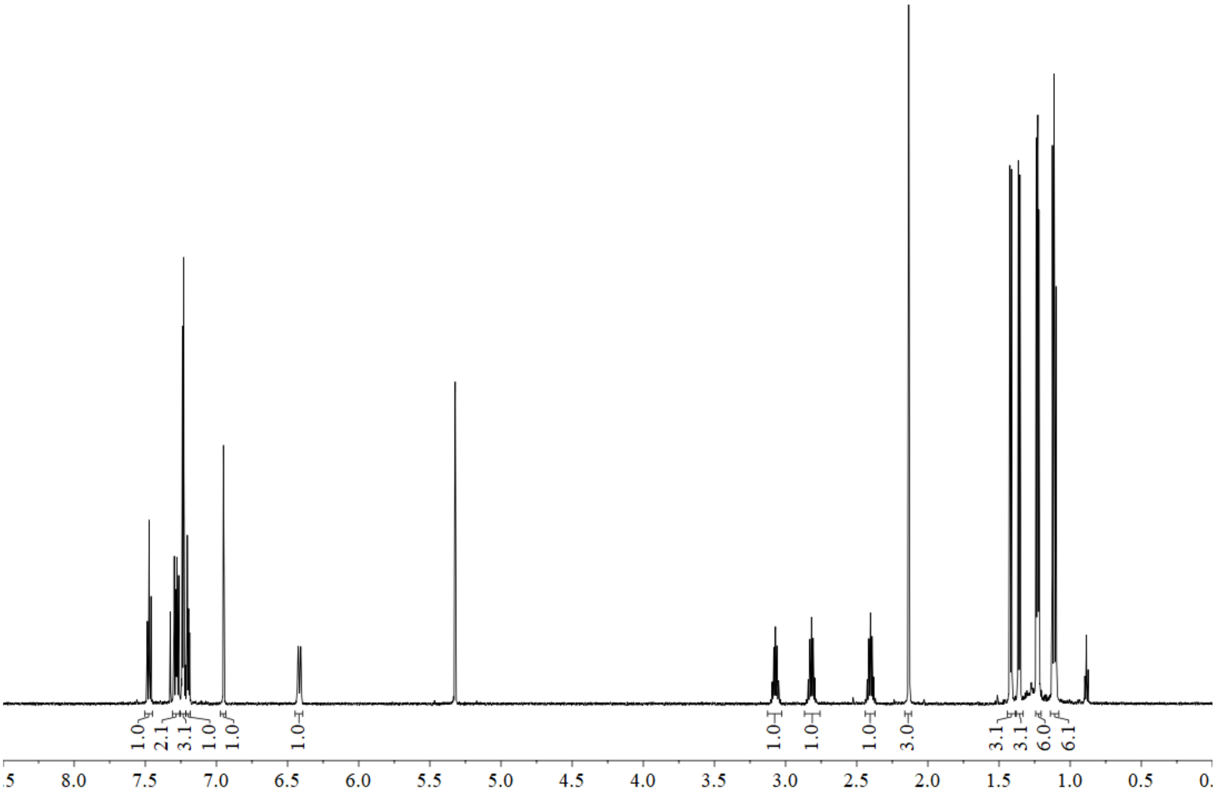

Figure S62a. ¹H NMR (600 MHz, 299 K, CD_2Cl_2) spectrum of compound **13**

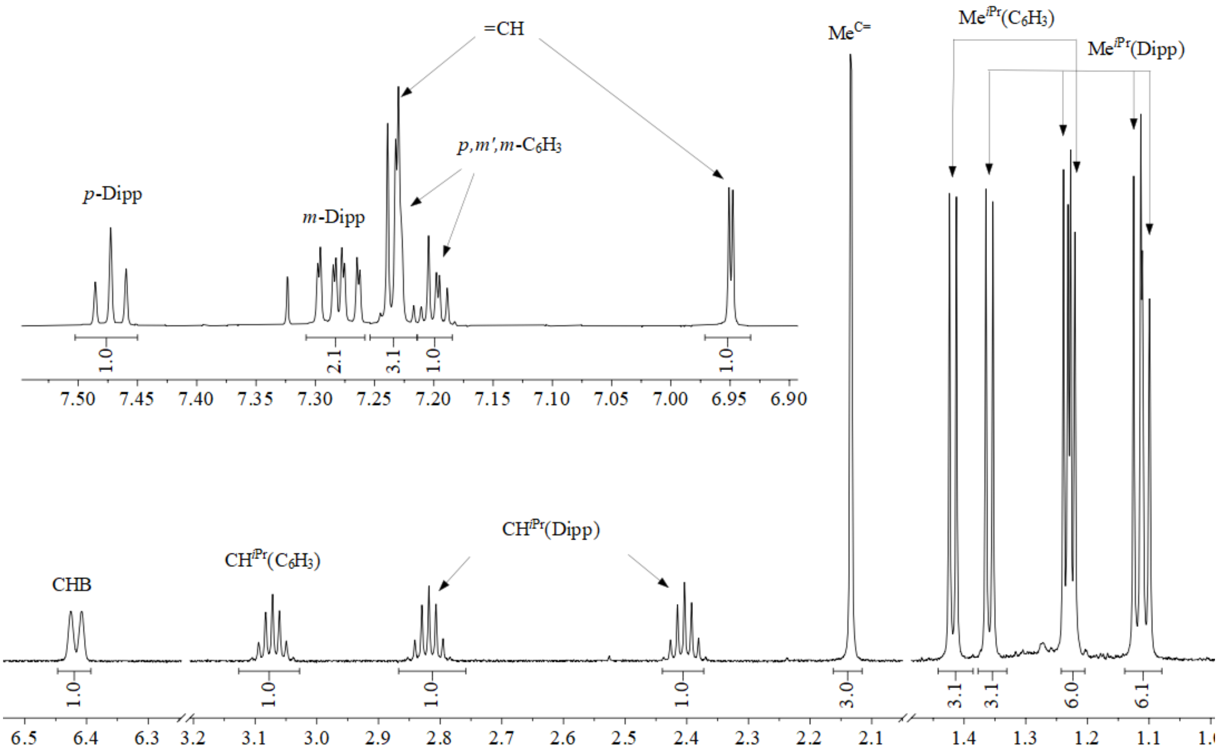

Figure S62b. ¹H NMR (600 MHz, 299 K, CD_2Cl_2) spectrum of compound **13**

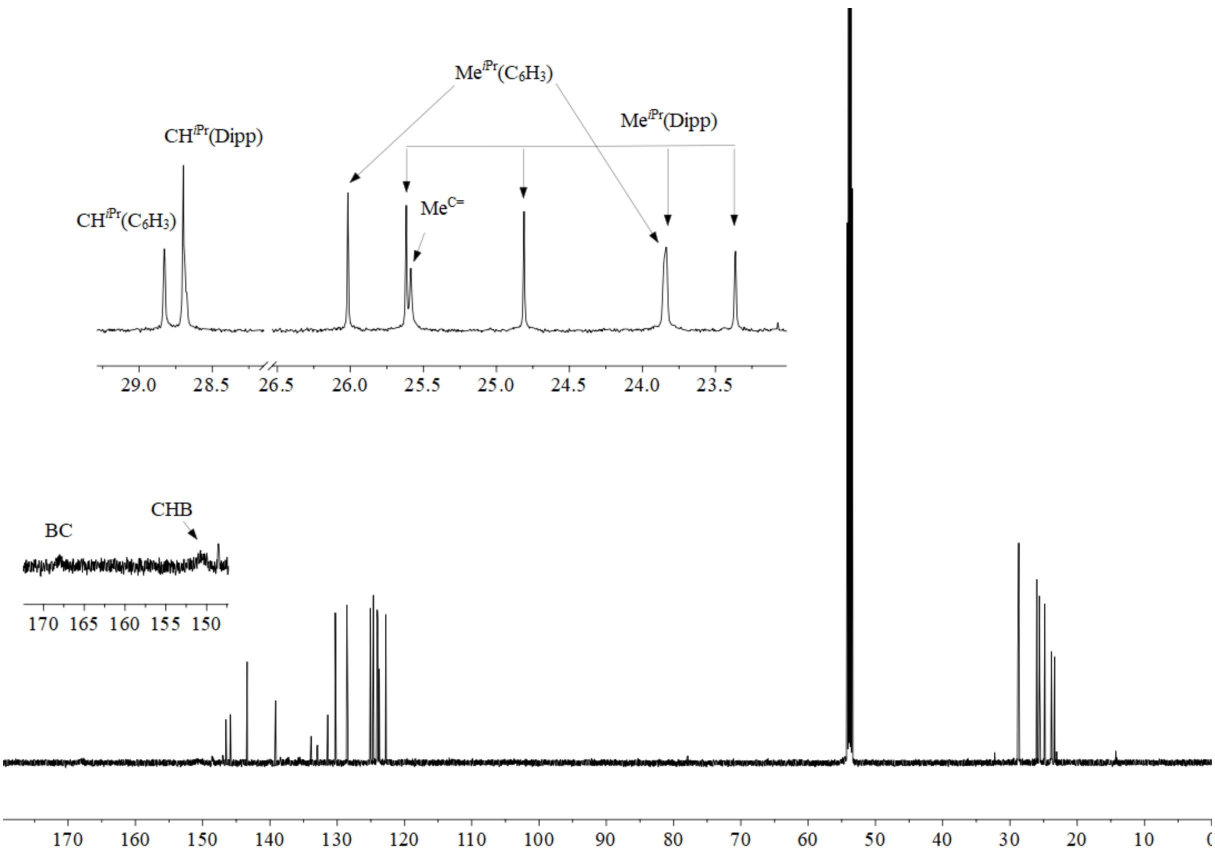

Figure S63a. $^{13}C\{^1H\}$ NMR (151 MHz, 299 K, CD_2Cl_2) spectrum of compound **13**

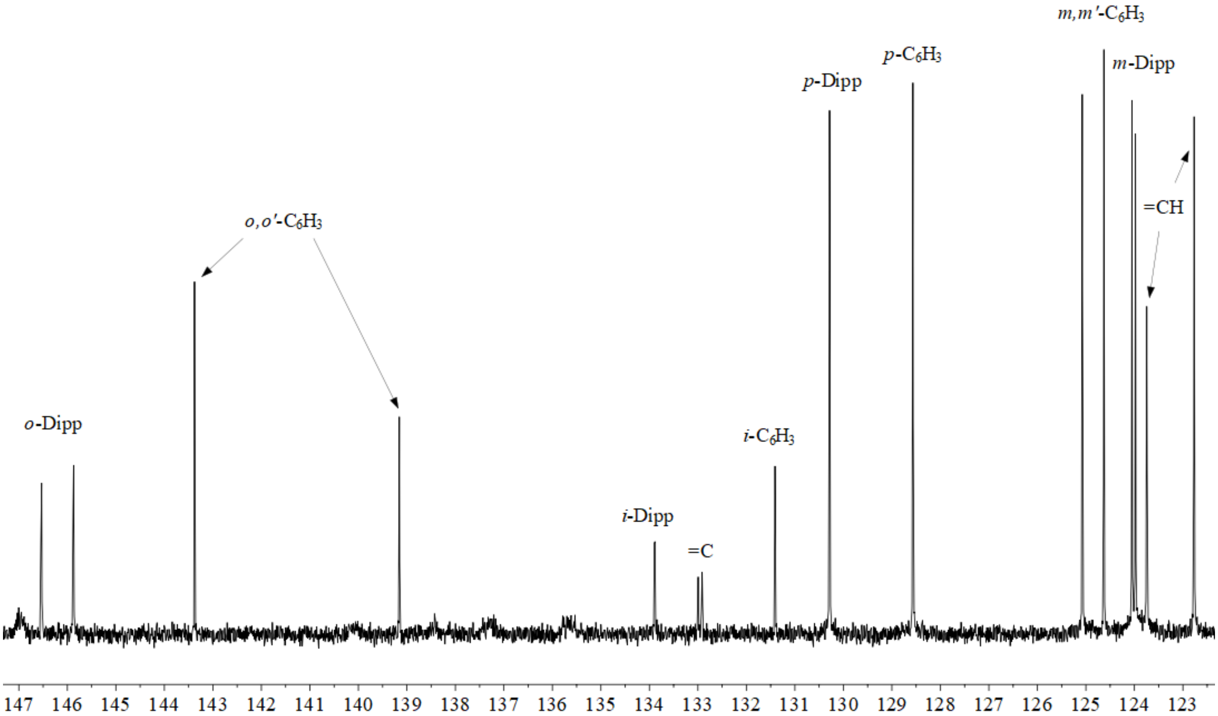

Figure S63b. $^{13}C\{^1H\}$ NMR (151 MHz, 299 K, CD_2Cl_2) spectrum of compound **13**

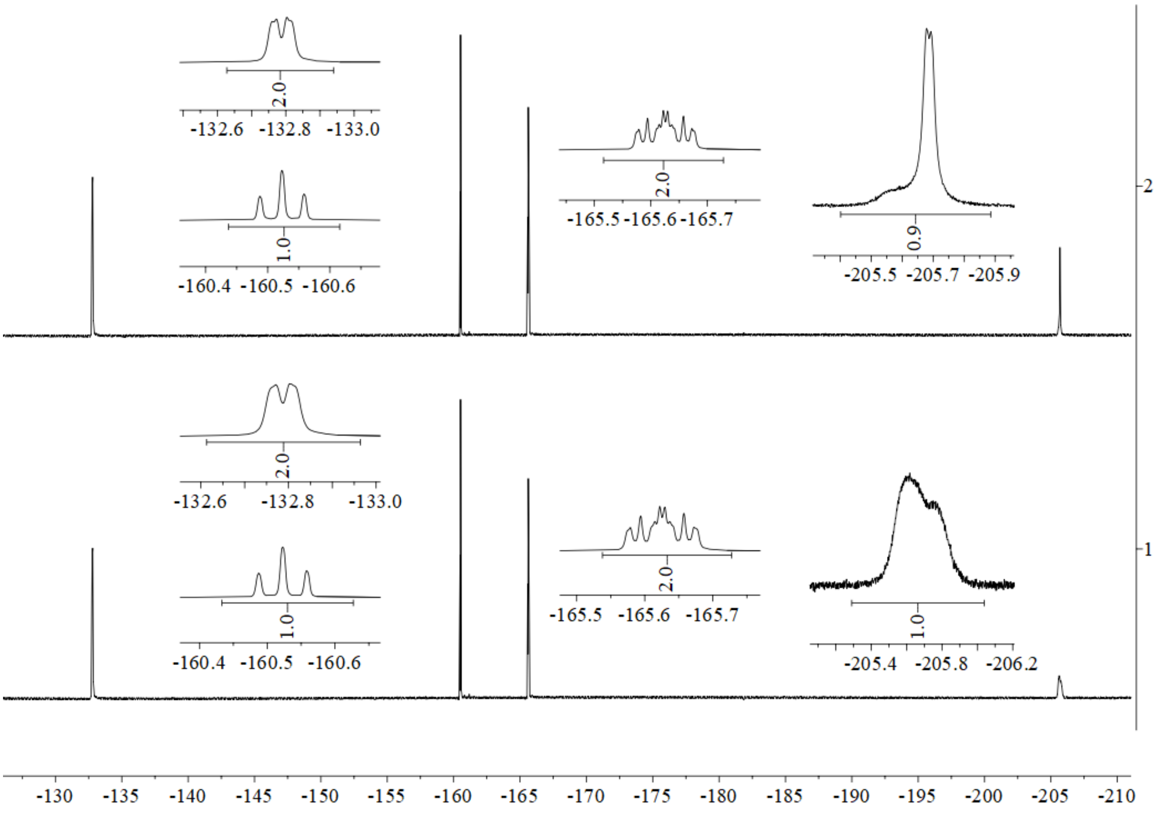

Figure S64. (1) ^{19}F and (2) $^{19}F\{^{11}B\}$ NMR (564 MHz, 299 K, CD_2Cl_2) spectra of compound **13**

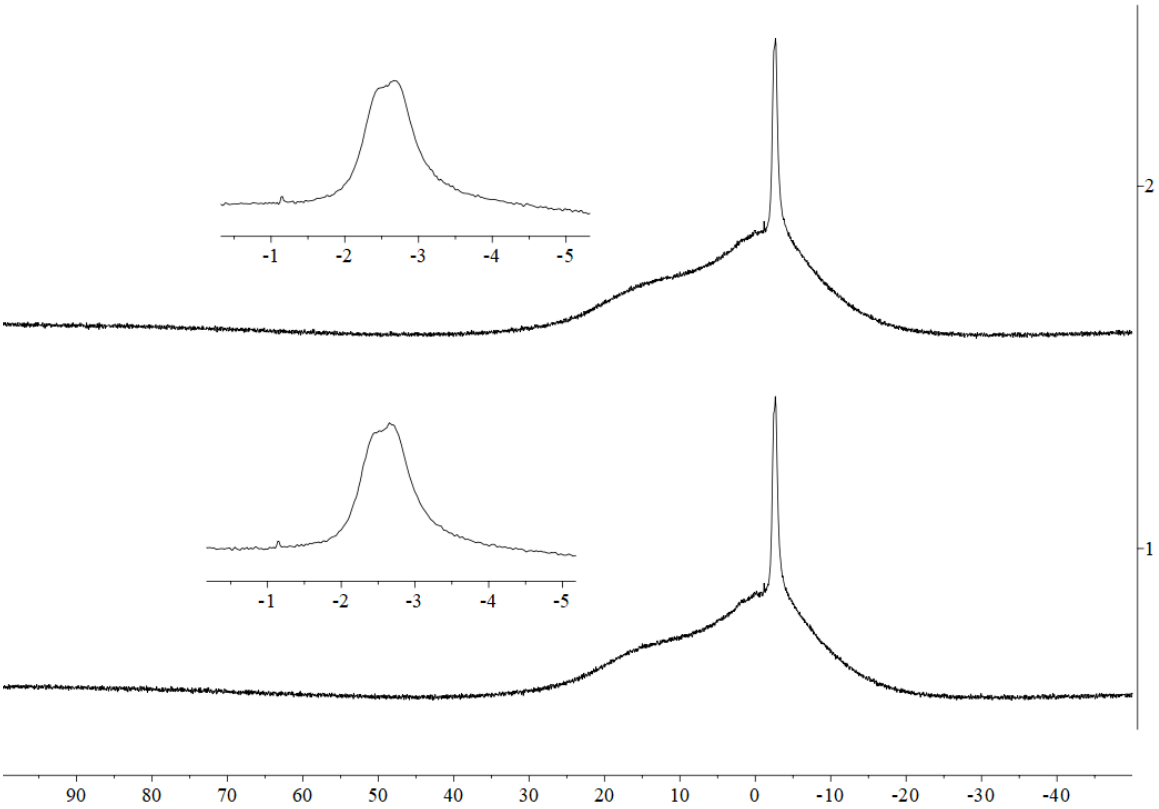

Figure S65. (1,2) ^{11}B and $^{11}B\{^{1}H\}$ NMR (192 MHz, 299 K, CD_2Cl_2) spectra of compound **13**

X-ray crystal structure analysis of compound 13 (erk10270): A colorless, prism-like specimen of $C_{33}H_{33}BF_6N_2$, approximate dimensions 0.079 mm x 0.136 mm x 0.330 mm, was used for the X-ray crystallographic analysis. The X-ray intensity data were measured on a single crystal diffractometer Bruker D8 Venture Photon III system equipped with a micro focus tube Mo ImS (MoKα, λ = 0.71073 Å) and a MX mirror monochromator. A total of 595 frames were collected. The total exposure time was 3.31 hours. The frames were integrated with the Bruker SAINT software package using a narrow-frame algorithm. The integration of the data using a monoclinic unit cell yielded a total of 70471 reflections to a maximum θ angle of 27.48° (0.77 Å resolution), of which 6727 were independent (average redundancy 10.476, completeness = 99.5%, R_{int} = 7.47%, R_{sig} = 3.43%) and 5412 (80.45%) were greater than $2\sigma(F^2)$. The final cell constants of a = 10.2547(2) Å, b = 17.4775(4) Å, c = 16.6351(4) Å, β = 98.4970(10)°, volume = 2948.73(11) $Å^3$, are based upon the refinement of the XYZ-centroids of 9918 reflections above 20 σ(I) with 4.661° < 2θ < 54.69°. Data were corrected for absorption effects using the Multi-Scan method (SADABS). The ratio of minimum to maximum apparent transmission was 0.959. The calculated minimum and maximum transmission coefficients (based on crystal size) are 0.9670 and 0.9920. The structure was solved and refined using the Bruker SHELXTL Software Package, using the space group $P2_1/n$, with Z = 4 for the formula unit, $C_{33}H_{33}BF_6N_2$. The final anisotropic full-matrix least-squares refinement on F^2 with 386 variables converged at R1 = 3.85%, for the observed data and wR2 = 10.06% for all data. The goodness-of-fit was 1.037. The largest peak in the final difference electron density synthesis was 0.305 $e^-/Å^3$ and the largest hole was -0.190 $e^-/Å^3$ with an RMS deviation of 0.042 $e^-/Å^3$. On the basis of the final model, the calculated density was 1.312 g/cm^3 and F(000), 1216 e^-. CCDC Nr.: 2157866.

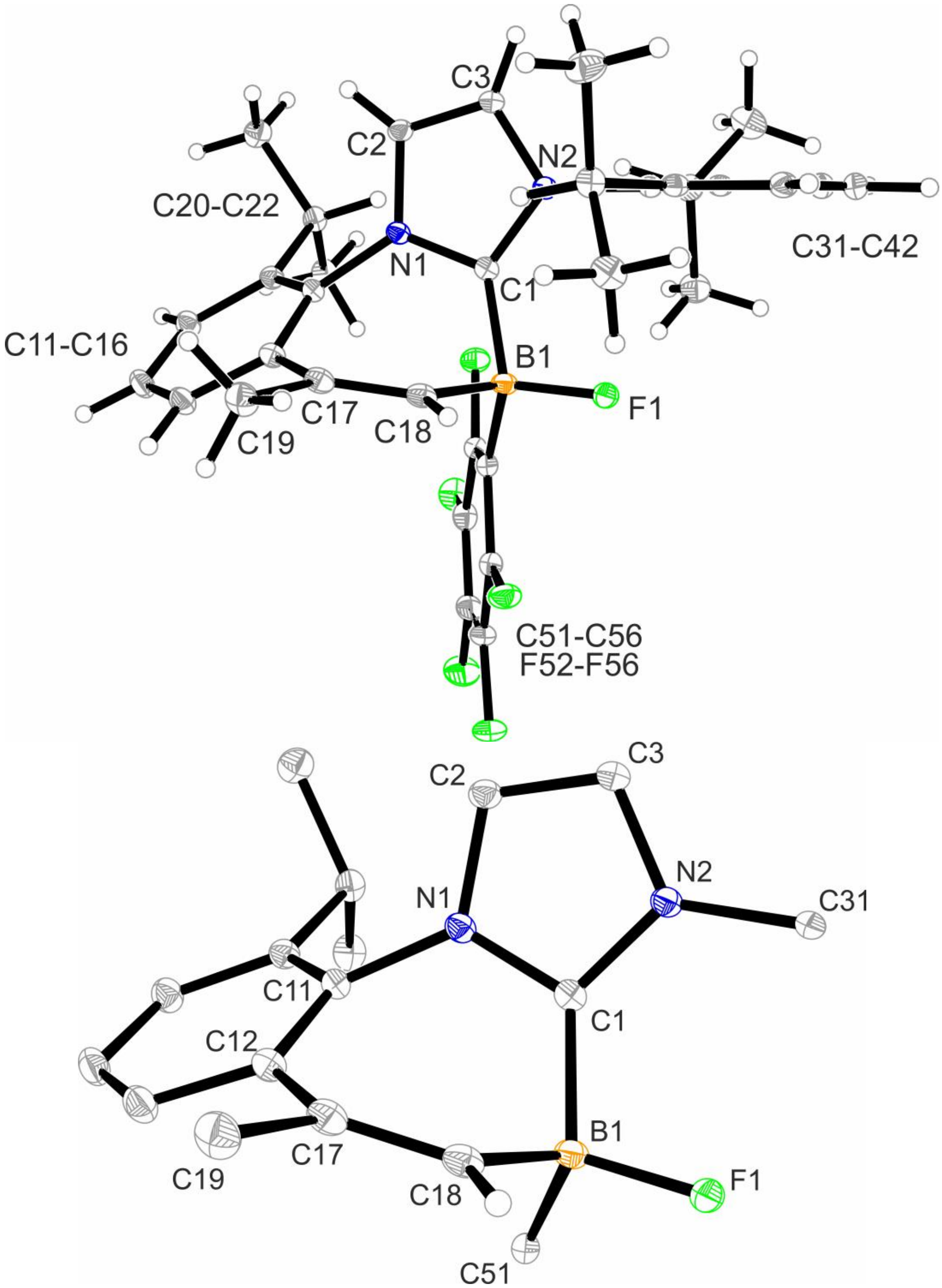

Figure S66. Crystal structure of compound **13** (thermal ellipsoids: 30% probability)

12. Initial studies on (bpy)Cu^I/[B]NO• catalysed oxidation of alkyl alcohols

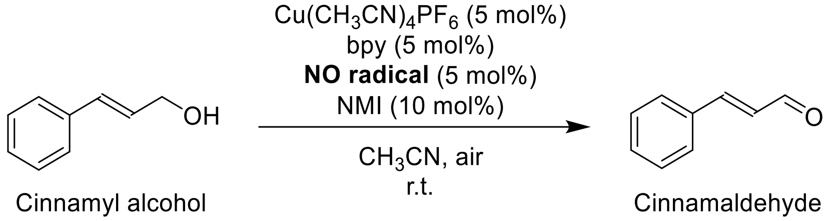

Entry	Conditions[a]	Yield[b]
1	without NO radical	< 1.0 %
2	with TEMPO, 6 h	96.5 %
3	with [B]NO• **5a**, 4 days	6.0 %
4	with [B]NO• **6a**, 15 h	62.3 %
5	with [B]NO• **6a**, 39 h	72.0 %
6	with [B]NO• **6a**, 4 days	83.1 %

NO radical
TEMPO; compound **5a**/**6a**

bpy 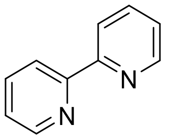

NMI 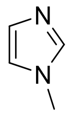

[a]General conditions: alcohol (0.2 mmol), Cu^I (0.01 mmol), bpy (0.01 mmol), NO radical (0.01 mmol), NMI (0.02 mmol), CH_3CN (1.0 mL). [b]Yield was determined by ^1H NMR using CH_2Br_2 as internal standard.

General procedure:

Cinnamyl alcohol (26.8 mg, 0.2 mmol), $Cu(CH_3CN)_4PF_6$ (3.7 mg, 0.01 mmol), bpy (1.6 mg, 0.01 mmol), NO radical (0.01 mmol) and NMI (2.0 μL, 0.02 mmol) were mixed and CH_3CN (1.0 mL) was added. The mixture was stirred under air at r.t. and monitored by TLC. After certain reaction time, the reaction mixture was diluted with CH_2Cl_2 and filter through a pad of SiO_2. Then the solvent was removed in vacuo and the obtained crude product was dissolved in $CDCl_3$ [with CH_2Br_2 (14.0 μL, 0.02 mmol) as internal standard] to characterize the resulting solution by ^1H NMR experiment.

[*Comment for entry 3: part of compound **5a** was recovered from the reaction mixture (ca. 4 mg crystals - ca. 60 % based on the initial amount of **5a**)*]

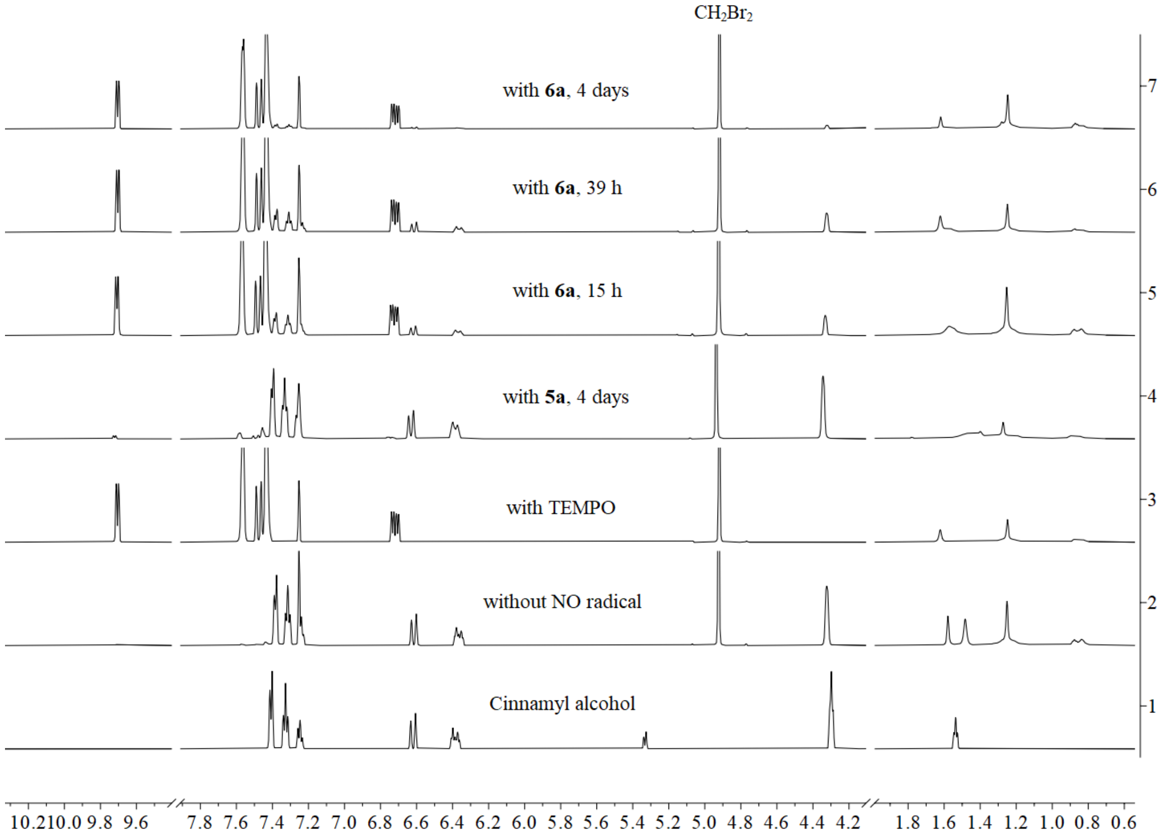

Figure S67. (1,2,3,4,5,6,7) ^{1}H NMR (599 MHz, 299 K, $CDCl_3$) spectra of (1) cinnamyl alcohol and (2-7) the obtained crude product (entries 1-6)

References

1. Bruker AXS (2019) *APEX3 Version 2019.1-0*, *SAINT Version 8.40A* and *SADABS Bruker AXS area detector scaling and absorption correction Version 2016/2*, Bruker AXS Inc., Madison, Wisconsin, USA.

2. G. M. Sheldrick, SHELXT – Integrated space-group and crystal-structure determination, *Acta Cryst.*, 2015, **A71**, 3.

3. G. M. Sheldrick, *Crystal structure refinement with SHELXL, Acta Cryst.*, 2015, **C71 (1)**, 3.

4. Bruker AXS (1998) *XP – Interactive molecular graphics, Version 5.1*, Bruker AXS Inc., Madison, Wisconsin, USA.

5. C. Chen, C. G. Daniliuc, G. Kehr, and G. Erker, Formation and Cycloaddition Reactions of a Reactive Boraalkene Stabilized Internally by N-Heterocyclic Carbene. *Angew. Chem. Int. Ed.* 2021, **60**, 19905.

6. J. R. Hwu, A. R. Das, C. W. Yang, J.-J. Huang, and M.-H. Hsu, 1,2-Eliminations in a Novel Reductive Coupling of Nitroarenes to Give Azoxy Arenes by Sodium Bis(trimethylsilyl)amide. *Org. Lett*. 2005, **7**, 3211.

7. a) K. Nakahara, S. Iwasa, J. Iriyama, Y. Morioka, M. Suguro, M. Satoh, and E. J. Cairns, Electrochemical and spectroscopic measurements for stable nitroxyl radicals. *Electrochimica Acta* 2006, **52**, 921; b) J. B. Gerken, and S. S. Stahl, High-Potential Electrocatalytic O_2 Reduction with Nitroxyl/NO_x Mediators: Implications for Fuel Cells and Aerobic Oxidation Catalysis. *ACS Cent. Sci*. 2015, **1**, 234; c) E. Tretyakov, A. Keerthi, M. Baumgarten, S. Veber, M. Fedin, D. Gorbunov, I. Shundrina, and N. Gritan, The Design of Radical Stacks: Nitronyl-Nitroxide-Substituted Heteropentacenes. *ChemistryOpen* 2017, **6**, 642.

8. T. Suga, Y.-J. Pu, K. Oyaizu, and H. Nishide, Electron-Transfer Kinetics of Nitroxide Radicals as an Electrode-Active Material. *Bull. Chem. Soc. Jpn*. 2004, **77**, 2203.

9. S. Stoll, and A. Schweiger, EasySpin, a comprehensive software package for spectral simulation and analysis in EPR. *J. Magn. Reson*. 2006, **178**, 42.

10. MATLAB Release 2018b. (MathWorks, Inc., 2018).

11. M. Brustolon, Electron Paramagnetic Resonance: A Practitioner's Toolkit. John Wiley & Sons (2009).

12. J. Durant, and M. S. Jahan, EPR power saturation techniques and spectral differentiation are used to isolate and simulate radical species in UHMWPE. Nucl. Instruments Methods Phys. Res. Sect. B Beam Interact. with Mater. Atoms 2005, **236**, 160.

13. S. C. Drew, C. L. Masters, and K. J. Barnham, Alzheimer's Aβ peptides with disease-associated NTerminal modifications: Influence of isomerisation, truncation and mutation on Cu^{2+} coordination. PLoS One 5, (2010).

14. W. E. Antholine, D. H. W. Kastrau, G. C. M. Steffens, G. Buse, W. G. Zumft, and P. M. H. Kroneck, A comparative EPR investigation of the multicopper proteins nitrous-oxide reductase and cytochrome c oxidase. *Eur. J. Biochem*. 1992, **209**, 875.

15. J. A. Weil, and J. R. Bolton, Electron paramagnetic resonance: elementary theory and practical applications. John Wiley & Sons (2007).

16. S. Grimme, J. Antony, S. Ehrlich, and H. Krieg, A consistent and accurate ab initio parametrization of density functional dispersion correction (DFT-D) for the 94 elements H-Pu. *J. Chem. Phys*. 2010, **132**, 154104.

17. F. Weigend, and R. Ahlrichs, Balanced basis sets of split valence, triple zeta valence and quadruple zeta valence quality for H to Rn: Design and assessment of accuracy. *Phys. Chem. Chem. Phys.* 2005, **7**, 3297.

18. A. Allouche, Software News and Updates Gabedit — A Graphical User Interface for Computational Chemistry Softwares. *J. Comput. Chem*. 2012, **32**, 174.

19. C. J. S. M. Simpson, P. T. Griffiths, and M. Towrie, The dynamics of photolysis of alkyl nitrites from a dielectric surface. A comparison with gas phase results. *Chem. Phys. Lett.* 1995, **234**, 20.

20. R. Beringer and J. G. Castle, Jr. Magnetic Resonance Absorption in Nitric Oxide. *Phys. Rev.* 1950, **78**, 581.
